# Kinetic CRAC uncovers a role for Nab3 in determining gene expression profiles during stress

**DOI:** 10.1038/s41467-017-00025-5

**Published:** 2017-04-11

**Authors:** Rob van Nues, Gabriele Schweikert, Erica de Leau, Alina Selega, Andrew Langford, Ryan Franklin, Ira Iosub, Peter Wadsworth, Guido Sanguinetti, Sander Granneman

**Affiliations:** 1 Centre for Synthetic and Systems Biology (SynthSys), University of Edinburgh, Edinburgh, EH9 3BF UK; 2 School of Informatics, University of Edinburgh, Edinburgh, EH8 9AB UK; 3 UVO3 Ltd, Unit 25 Stephenson Road, St Ives, Cambridgeshire PE27 3WJ UK; 4 Institute of Cell Biology, University of Edinburgh, Edinburgh, EH9 3FF UK; 5 Institute for Molecular Plant Sciences, University of Edinburgh, Edinburgh, EH9 3BF UK

## Abstract

RNA-binding proteins play a key role in shaping gene expression profiles during stress, however, little is known about the dynamic nature of these interactions and how this influences the kinetics of gene expression. To address this, we developed kinetic cross-linking and analysis of cDNAs (χCRAC), an ultraviolet cross-linking method that enabled us to quantitatively measure the dynamics of protein–RNA interactions in vivo on a minute time-scale. Here, using χCRAC we measure the global RNA-binding dynamics of the yeast transcription termination factor Nab3 in response to glucose starvation. These measurements reveal rapid changes in protein–RNA interactions within 1 min following stress imposition. Changes in Nab3 binding are largely independent of alterations in transcription rate during the early stages of stress response, indicating orthogonal transcriptional control mechanisms. We also uncover a function for Nab3 in dampening expression of stress-responsive genes. χCRAC has the potential to greatly enhance our understanding of in vivo dynamics of protein–RNA interactions.

## Abstract (web summary)

Protein RNA interactions are dynamic and regulated in response to environmental changes. Here the authors describe ‘kinetic CRAC’, an approach that allows time resolved analyses of protein RNA interactions with minute time point resolution and apply it to gain insight into the function of the RNA-binding protein Nab3.

## Introduction

RNA-binding proteins (RBPs) control almost all aspects of gene expression, including the stability of the RNA, its structure, the rate at which the RNA is processed, how efficiently it is translated and its subcellular localization. Not surprisingly, because of these important functions, RBPs are often found associated with many diverse genetic and somatic diseases, including muscular disorders, autoimmune diseases, and cancer^1^. RBPs also play a very important role in adapting to dynamic environments, such as those encountered by microbes when exposed to stress. Survival under stress is contingent on the ability to rapidly reprogram gene expression and, while this ability has been largely attributed to the activity of transcription factors, it is becoming increasingly clear that RBPs also play a primary role in shaping gene expression response profiles by modulating RNA processing and decay^2^. RBPs involved in RNA decay are believed to play an important role during the first few minutes of the adaptation response during which major transcriptional reprogramming events happen^3, 4^. However, direct measurement of protein–RNA interactions during these early stages has so far proved elusive. Consequently, little is known about the contribution of individual RNA decay factors during rapid rewiring of the gene expression program in response to environmental changes.

In recent years, ultraviolet (UV) cross-linking and immunoprecipitation (CLIP) followed by deep sequencing has emerged as the main technology to map protein–RNA interactions in vivo^5^. UV-irradiation is used to forge covalent bonds (cross-links) between proteins and directly bound RNAs. Proteins of interest are then purified under stringent conditions and high-throughput sequencing of the cross-linked RNA enables mapping of the interaction sites. A number of CLIP-related techniques have been developed over the years, such as CRAC (cross-linking and analysis of cDNAs), iCLIP, and PAR-CLIP (photoactivatable ribonucleoside-enhanced crosslinking and immunoprecipitation)^6–8^. For CRAC the protein of interest is fused to a tandem affinity purification tag (HTP; His_6_-TEV-ProtA) to enable purification of cross-linked RNAs under completely denaturing conditions^6^. Recent advancements have enhanced the efficiency of the library preparation, increased the data complexity and improved the resolution of RNA-binding site detection^7–11^. Despite such advances, current protocols are ill-suited to quantitatively measure dynamic changes in protein–RNA interactions. Using current commercially available UV-irradiation equipment, the cross-linking step can take up to 30 min to reach the desired dose (depending on organism and wavelength)^12–14^. This limitation rules out measurements of the early stress responses, which can happen on the minute time scale^3, 4^. In addition, due to prolonged UV-irradiation, cells are exposed to major additional stresses, such as DNA damage, which can confound the results and insert a bias toward RNA transcripts that are specific for the irradiation conditions.

To tackle these problems, we have improved the original CRAC protocol and developed a UV-irradiation device that cross-links proteins to RNA in vivo in seconds. These advancements enabled us to perform quantitative time-resolved in vivo measurements of direct protein–RNA interactions at 1-min time-point resolution. We refer to this method as kinetic CRAC (χCRAC).

We have applied χCRAC to glucose-deprived *Saccharomyces cerevisiae* to investigate the dynamic interactions of the RBP Nab3 during the adaptation process. Nab3 is a component of the Nrd1-Nab3-Sen1 (NNS) transcription termination complex that is involved in degradation of diverse classes of lnc-RNAs, such as cryptic unstable transcripts (CUTs), Nrd1 unterminated transcripts, and various messenger RNAs (mRNAs) and in the maturation of snoRNAs^15, 16^. Depriving yeast of glucose results in significant changes in mRNA levels and a transcriptome-wide redistribution of NNS components^17–20^. χCRAC accurately detected these widespread changes in Nab3 binding; importantly, the high temporal resolution enabled us to document transient changes in cross-linking of Nab3 to many transcripts, indicating a potentially pervasive importance of termination factors in the early stages of stress response. We also uncover a role for Nab3 in regulating the expression kinetics of stress-responsive genes and found that Nab3 is required for suppression of retrotransposon transcription during late stages of the glucose deprivation response. This suggests that Nab3 could play an important role in maintaining genome integrity during stress.

## Results

### Very fast protein–RNA cross-linking in vivo

**Fig. 1:**
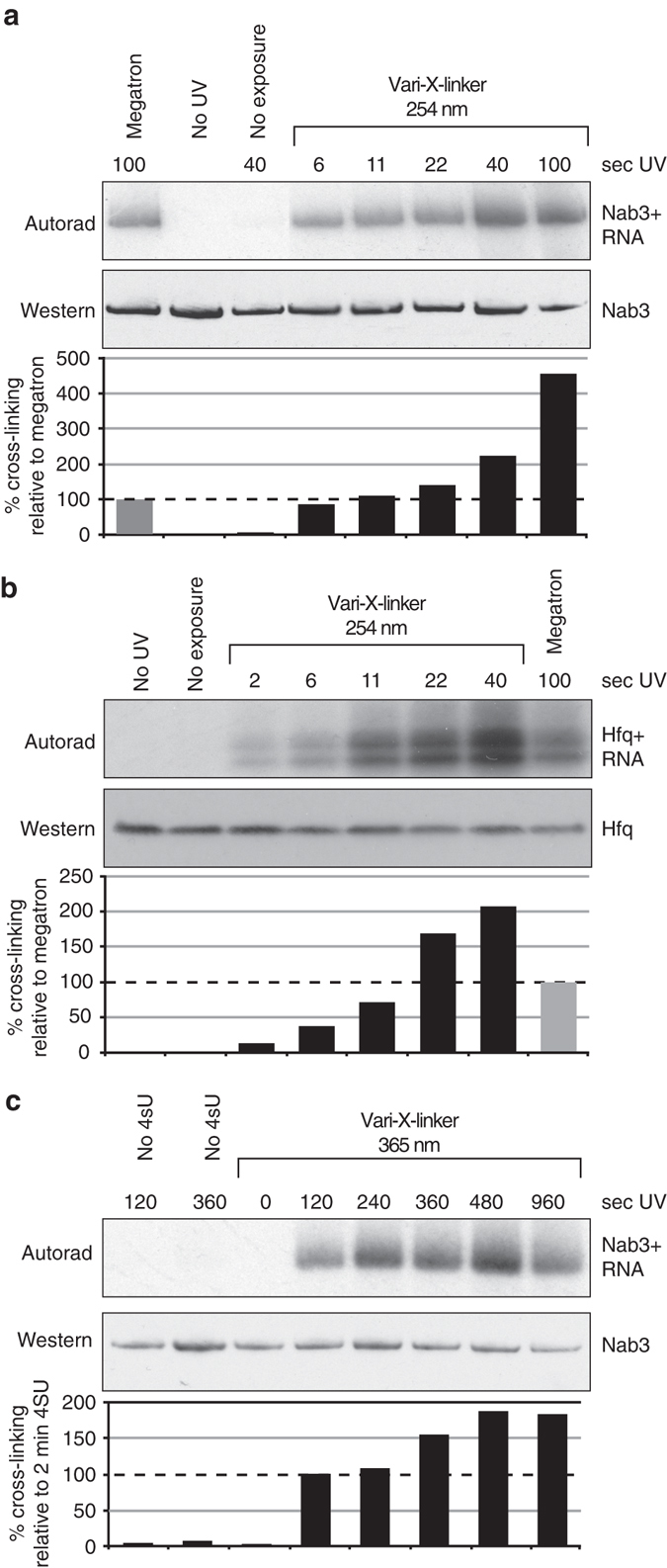
TheVari-X-linker cross-links proteins to RNA in seconds. **a** The Vari-X-linker standard lamps are ~10× more efficient in cross-linking proteins to RNA in vivo compared to the Megatron unit. Cells were UV irradiated in the Megatron for 100 s. Cross-linking in the Vari-X-linker was performed at the indicated times (seconds). The western blot shows that comparable amounts of Nab3 protein was purified during the CRAC experiments. The autoradiogram shows the ^32^P-labeled RNA cross-linked to Nab3 in each sample. These scans were used to quantify the level of cross-linking relative to the Megatron by normalizing the autoradiogram signal to the protein levels. **b** As in **a** but now monitoring the cross-linking of the *E. coli* Hfq protein. **c** Results of PAR-CLIP experiments performed using variable 365 nm UV-irradiation times, indicated in seconds. For experimental details, see the Methods section

To establish χCRAC, we developed a UV-irradiation apparatus (Vari-X-linker) to improve the in vivo protein–RNA cross-linking efficiency (Supplementary Fig. 1; see Methods for a more detailed description of the apparatus). To test the effectiveness of the Vari-X-linker, we performed CRAC experiments on yeast strains expressing HTP-tagged (His_6_-TEV-ProtA) Nab3 that were UV-irradiated in the Vari-X-linker or in the Megatron, which (to the best of our knowledge) is currently the most efficient UV cross-linker on the market for cross-linking cell cultures^12^. Our tests with the Vari-X-linker’s 254 nm lamps showed that it can cross-link yeast proteins to RNA in seconds, up to tenfold more efficiently than the Megatron (Fig. 1a). Combined with a cell filtration device that we developed, it is possible to cross-link cells and harvest 1 L of cells in ~1 min. We also tested the Vari-X-linker standard lamps on an *E. coli* strain expressing a His6-TEV-FLAG-tagged Hfq protein^21^, which showed a sevenfold improvement in cross-linking time (Fig. 1b). The Vari-X-linker can also be used with 365 nm lamps (350 W) to perform PAR-CLIP experiments, providing a high cross-linking efficiency after 2 min of UV-irradiation at considerably lower 4-thio-Uracil concentrations and shorter labeling times (see Methods for more details) (Fig. 1c).

Thus, we can perform time-resolved (PAR-)CLIP/CRAC experiments on very short time-scales, enabling the measurement of dynamic protein–RNA interactions in living cells with high temporal resolution.

**Fig. 2:**
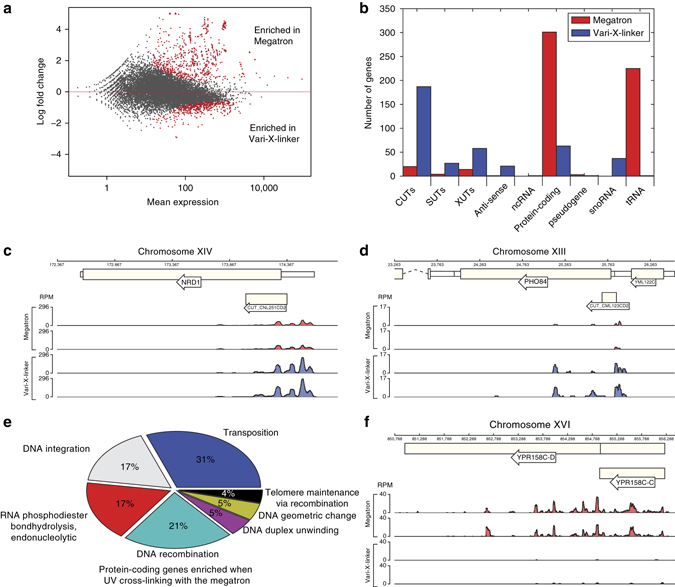
TheVari-X-linker allows better detection of Nab3 binding to short-lived RNA species and reduces the induction of the DNA damage response. **a** DESeq2 differential expression analysis of Megatron (two replicates) and the Vari-X-linker Nab3 CRAC data (four replicates). The *red dots* in the plot indicate the transcripts that differentially cross-linked in data from the two different UV cross-linkers. Transcripts with positive log2-fold change values are enriched in the Megatron data, whereas transcripts with negative log2-fold change values are enriched in the Vari-X-linker data. **b** Feature analyses of differentially cross-linked transcripts. The bar plot shows the number of genes (*y*-axis) in each genomic feature (*x*-axis) that were found to be significantly enriched (adjusted *p*-value < = 0.05) in the Megatron (*red bars*) and the Vari-X-linker (*blue bars*) data. SUTs: Stable Uncharacterized Transcripts. XUTs: Xrn1 Unstable Transcripts. CUTs: Cryptic Unstable Transcripts. ncRNA: non-coding RNA. **c**,**d** Genome browser examples of CUTs that show higher Nab3 binding in the Vari-X-linker data. The *y*-axis shows reads per million (RPM). **e** Pie chart showing the significantly enriched GO-terms (FDR < 0.01) in the ~300 protein-coding transcripts enriched in the Megatron data. **f** Genome browser graph of Nab3 cross-linking to transcripts originating from retrotransposable elements *YPR158C-C* and *YPR158C-D*. The *y*-axis shows reads per million (RPM)

Differential expression analysis of Nab3 CRAC data generated using the Megatron and the Vari-X-linker revealed significant differences between the two UV-irradiation conditions (Fig. 2a, DESeq2^22^; adjusted *p*-values < = 0.05). The Vari-X-linker data were more highly enriched for short-lived lncRNA species (Stable Uncharacterized Transcripts (SUTs), Xrn1 Unstable Transcripts (XUTs), Cryptic Unstable Transcripts (CUTs), and anti-sense transcripts) (Fig. 2b). This suggests that very short UV-irradiation times significantly improve the recovery of these unstable lncRNAs, as shown for two known CUTs that originate from the Nrd1 and Pho84 genes (Fig. 2c,d)^23, 24^. The ~300 protein-coding genes enriched in the Megatron data (Fig. 2b) were highly enriched for genes that are upregulated during DNA damage (Fig. 2e,f; FDR < 0.01). Although the steady state levels of retrotransposons did not significantly change during the 100 s UV-irradiation in the Megatron (Supplementary Fig. 2), the DESeq2 analyses revealed significantly higher cross-linking of Nab3 to these transcripts, suggesting that Nab3 actively targets these transcripts during long UV-irradiation times (Fig. 2e,f). Additionally, we also detected a significant enrichment of almost all transfer RNAs (tRNAs) in the Megatron data, which we believe reflects Nab3-dependent degradation of tRNAs that accumulate in the nucleus during the DNA damage response^25^. While these data suggest a role for the NNS complex in regulating DNA damage response and suppressing retrotransposon transcription (see below), it also illustrates that long UV-irradiation times increase the likelihood of detecting alterations in transcription that are the result of the activation of the DNA damage response.

### Monitoring in vivo dynamics of protein–RNA interactions

**Fig. 3:**
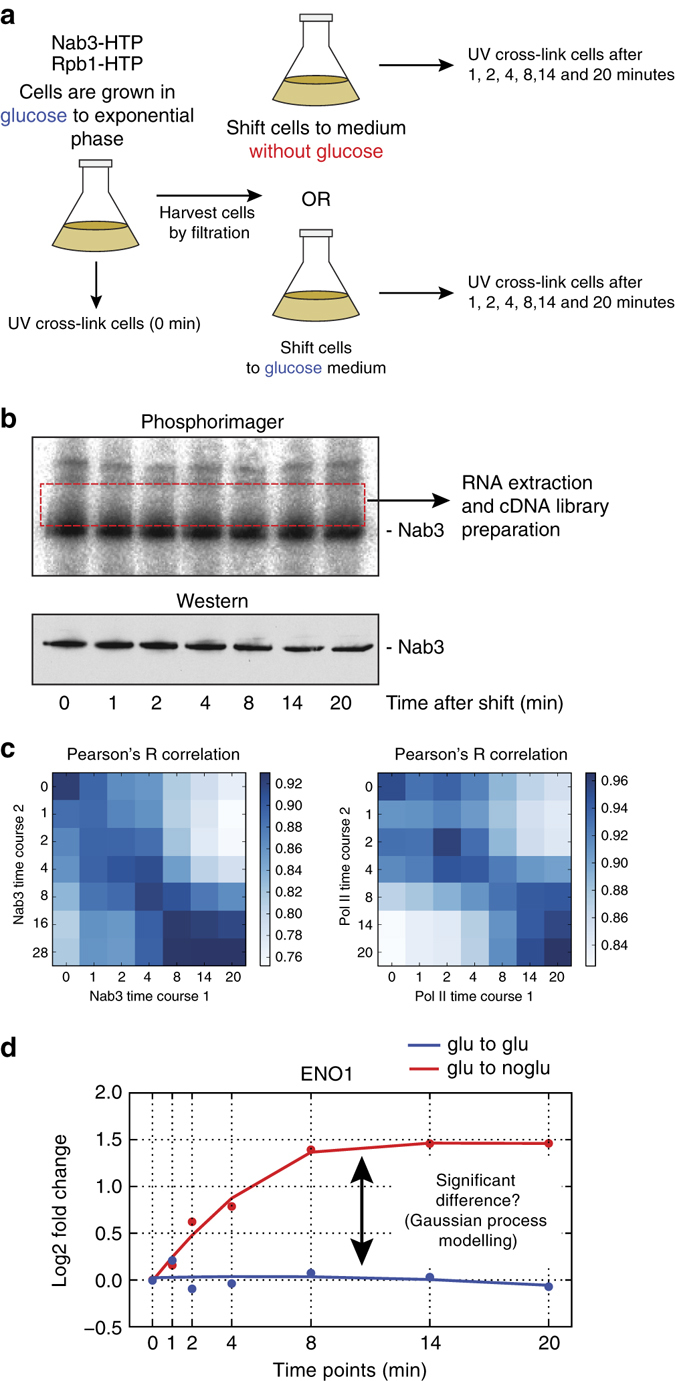
Time-resolved cross-linking analyses during glucose deprivation. **a** Outline of experimental set-up. Cells are grown in glucose medium to exponential phase. A fraction is cross-linked and harvested (*t* = 0 sample). The rest is rapidly harvested by filtration and transferred into medium lacking glucose or medium with glucose (control experiment). Subsequently, the cells were UV irradiated at the indicated times. **b** Nab3 cross-linking to RNA during glucose deprivation. Shown is a result of a typical χCRAC experiment. After resolving purified protein with RNAse digested radiolabeled cross-linked RNA on NuPAGE gels, the cross-linked RNA is detected by autoradiography. Western blotting was performed to ensure that comparable amounts of protein was recovered in each time-point. A cDNA library was subsequently prepared from RNA extracted from a single membrane slice containing RNA from all time-points. **c** Early time-points are highly correlated. The heat map shows a Pearson’s R correlation analysis of each individual time-point from Pol II and Nab3 replicate χCRAC experiments. The darker the *blue color*, the higher the Pearson’s correlation. Pearson correlations were calculated from log2 transformed FPKM (fragments per kilobase transcript per million reads) values. **d** A Gaussian process model was used to select genes that show significantly different cross-linking profiles between the control (glucose to glucose) and treated (glucose to no glucose) experiment. The example shows the *ENO1* Pol II cross-linking profiles from a control (*blue*) and treated (*red*) experiment. The *x*-axis shows the time-points (minutes) at which samples were taken during the time-course. All data were normalized to the 0 time-point. The *y*-axis shows the log2-fold changes in FPKMs

Yeast cells deprived of glucose redistribute NNS components over the transcriptome^17^. Therefore, to test the feasibility of our χCRAC method, we measured changes in Nab3 cross-linking during glucose deprivation. Because Nab3 co-transcriptionally binds RNA, we also performed χCRAC on RNA polymerase II using a strain expressing an HTP-tagged Rpo21 subunit^26^. This enabled us to determine how well Nab3 binding correlates with changes in Pol II transcription. To measure changes in steady-state RNA levels, we performed RNA-Seq on ribosomal RNA-depleted total RNA. We devised a simple experimental set-up that would enable us to rapidly shift cells to a new medium (Fig. 3a). Cells were grown to exponential phase in glucose medium after which a fraction of the cells were harvested (*t* = 0 time-point). The rest was rapidly harvested by filtration and transferred to a flask with medium lacking glucose. After the shift, cells were cross-linked at various time-points. To control for changes in gene expression caused by the filtration process, we performed experiments where filtered cells were transferred back to glucose containing medium. To accurately quantify differences in cross-linking between time-points, we made several improvements to the original CRAC protocol^6^ to reduce sequence representation biases (Supplementary Fig. 3) and to improve the preparation of complementary DNA (cDNA) libraries (see Methods). After resolving the purified protein–RNA complexes on SDS-polyacrylamide gel electrophoresis (PAGE) gels, they were transferred to nitrocellulose (Fig. 3b). Western blotting was performed to assess the efficiency of protein recovery after the nickel purification steps (Fig. 3b). RNA from each sample was ligated to 5′ adapters with unique barcodes (Supplementary Table 2). To reduce technical noise, cross-linked RNAs from all the time-points were pooled by extracting RNA from a single membrane slice containing the radioactive signal just above the main bands (Fig. 3b, red dashed rectangle) from which a single cDNA library was generated.

Statistical analyses of biological replicates revealed that χCRAC generates highly reproducible results (Fig. 3c, Supplementary Fig. 4). The early (1–4) min time-points were also highly correlated (Fig. 3c), followed by a sharp drop in correlation coefficients, suggesting that major changes in cross-linking profiles take place shortly after the first 4 min of glucose deprivation.

To identify transcripts that showed significant differential cross-linking profiles between the control (glucose to glucose) and treated (glucose to no glucose), we fitted a Gaussian process (GP) regression model to both time series^27, 28^ to compute the likelihood that the control and treated originated from different profiles (see Methods; Fig. 3d).

Finally, to validate our findings we used the anchor-away system^29^. By tagging Nab3 with the FKBP12-rapamycin-binding (FRB) domain in the anchor-away strain, we were able to rapidly and effectively deplete Nab3 from the nucleus by adding rapamycin to the glucose medium 1 h before shifting the cells to medium lacking glucose (see Methods, Supplementary note 1, Supplementary Figs. 5 and 6).

### χCRAC provides insights into transcription kinetics

**Fig. 4:**
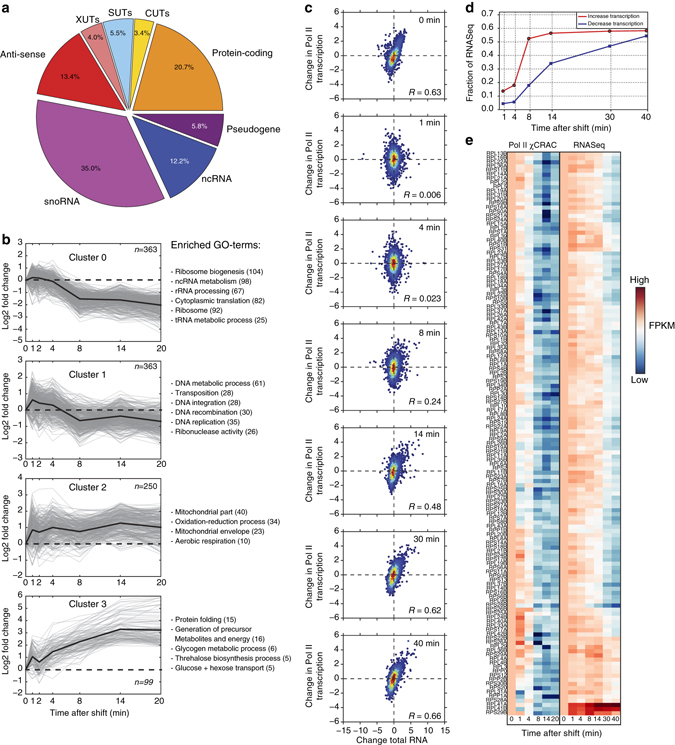
RNA polymerase II χCRAC shows rapid changes in Pol II transcription during glucose deprivation. **a** The pie chart shows what percentage of each RNA class showed changes in Pol II transcription during glucose deprivation. **b** Pol II cross-linking profiles for protein-coding genes were generated by K-means clustering, performed using STEM^60^. Only mRNA profiles were selected that showed a maximum fold-change of at least 1.5 and had a mean pairwise correlation over two biological replicates of 0.7. The *gray* lines indicate profiles from individual genes. The *dark black lines* show the average profile for each cluster. Enriched gene ontology (GO) terms are indicated on the *right* side of each graph. The *y*-axis shows the log2-fold change of each time-point relative to time-point 0 (glucose sample). The *x*-axis shows the time-points that were analyzed (minutes) during the glucose starvation time-course. **c** Comparison of changes in Pol II transcription (*y*-axis) to changes in total RNA levels (*x*-axis) for several time-points. *Red* and *blue* colored dots indicate high and low data point density, respectively. To compare the data sets, we Z-normalized the fragments per kilobase transcript per million reads (FPKM) values. *R*-values indicate Pearson correlations. **d** RNA degradation is a rate limiting step during the glucose deprivation response. For each time-point, we calculated what fraction of genes that showed an increase or decrease in transcription (>=2-fold) also showed a similar change in the RNASeq data (*y*-axis). The *x*-axis shows the time-points (minutes) after induction of glucose starvation that were analyzed. The *red line* shows the results for the transcriptionally upregulated genes. The *blue line* shows the results for transcriptionally downregulated genes. **e** Transcription of most r-protein genes is shut down within 4–8 min, but total RNA levels only decrease many minutes later. The *x*-axis shows the time-points (minutes) after induction of glucose starvation that were analyzed. The heat map shows a side-by-side comparison of Pol II χCRAC and RNASeq r-protein data from a glucose starvation time-course. The higher the FPKM, the redder the color. The lower the FPKM the darker blue the color. Note that the RNASeq data has two longer time-points (30 and 40 min)

GP analyses identified 2431 Pol II transcripts that showed significant changes in Pol II cross-linking profiles after the shift to medium lacking glucose (Bayes Factor > 10 supporting different response dynamics (see Methods)). The largest changes were observed in snoRNAs, protein-coding genes and anti-sense RNAs (Fig. 4a). To determine how well our data agrees with previous transcriptome-wide studies, we analyzed the Pol II cross-linking profiles for protein-coding genes in more detail. For the majority of protein-coding genes the changes in profiles could be summarized into 4 K-means clusters (Fig. 4b). Genes in all four clusters were enriched for distinct GO-terms. Cluster 0 is highly enriched for genes from the Ribiregulon (ribosomal proteins (r-proteins) and ribosome assembly factors) that are downregulated during glucose deprivation. Cluster 1 is enriched for genes involved in DNA metabolism and transposition. Clusters 2 and 3 contain many stress responsive genes and genes involved in respiration that are known to be upregulated during glucose starvation^18, 20^. These results are in excellent agreement with previous studies^18, 20^, demonstrating that Pol II χCRAC can accurately measure changes in gene expression at high temporal resolution.

Another reason for performing Pol II χCRAC studies, was to determine whether the data could potentially be used to develop statistical models for estimating RNA half-lives or to generate mechanistic models for RNA transcription and processing during stress. As a first step in this direction, we asked how well the Pol II mRNA χCRAC data (Fig. 4c, *y*-axis) correlated with changes in the levels of total mRNA as measured by RNASeq at each time point (Fig. 4c, *x*-axis). Only at the 0 (glucose) and late 30 and 40-min (no glucose) time-points a highly positive correlation between changes in total RNA levels and changes in Pol II transcription (Pearson’s *R* = 0.63 to 0.68; *p*-values < 0.01) was observed. These results suggest that it takes about 30–40 min to adjust total RNA levels to mirror Pol II transcription levels. After about 14 min of glucose deprivation ~60% of the transcriptionally upregulated genes also showed a comparable increase in total RNA levels. This percentage only marginally increased at later time-points, (Fig. 4d, red line). This indicates that transcription regulation plays a dominant role during the first 8 min of the glucose deprivation response. In contrast, many genes with decreasing transcription levels only showed a similar decrease in total RNA levels during late stages of the adaptation response, suggesting that the adjustment of steady-state RNA levels for these genes is relatively slow (Fig. 4c and blue line in Fig. 4d). This especially was the case for r-protein coding genes: We observed that transcription of most r-proteins was reduced to basal level already 8 min after the shift, whereas total RNA levels of most r-protein transcripts decreased more slowly (Fig. 4e). The average mRNA half-life of r-protein coding transcripts during rapid glucose removal was estimated to be around 16 min^30^, which is consistent with the slow decrease in total mRNA levels that we observed during the adaptation response. Interestingly, although both *RPL41A* and *RPL41B* were downregulated on the transcriptional level, total mRNA level of these transcripts increased during glucose starvation (Fig. 4e).

Collectively, our data indicate that during glucose deprivation the bulk of the transcriptional changes take place within the first 8 min and that degradation of transcripts from downregulated genes could be a rate-limiting step during the adaptation process.

### Nab3-RNA interaction dynamics during glucose starvation

**Fig. 5:**
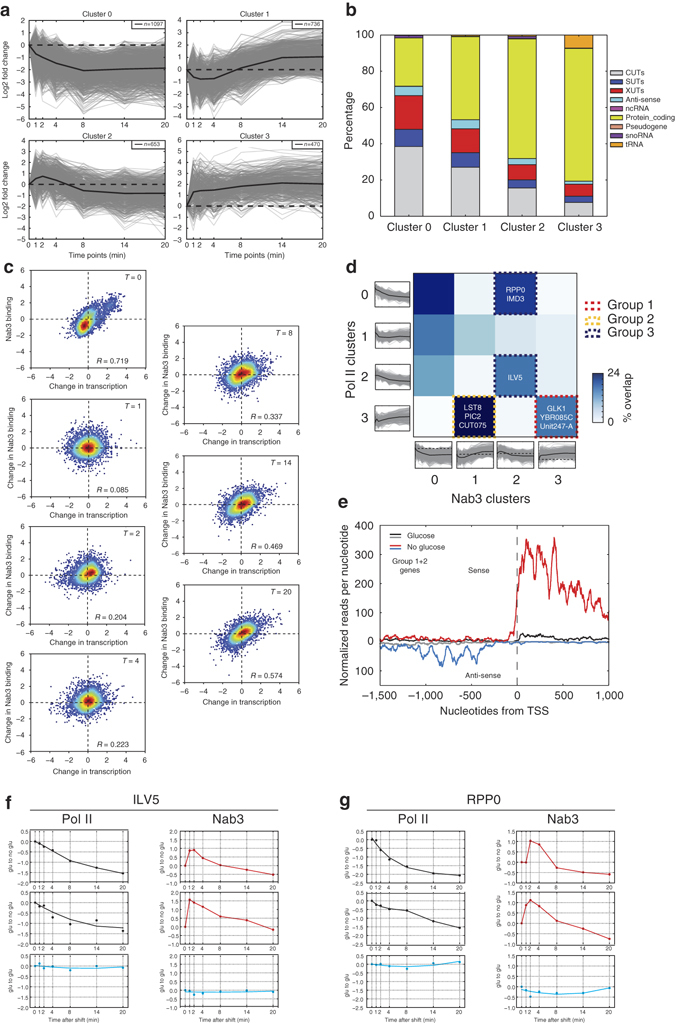
Dynamic binding of Nab3 to many transcripts during glucose deprivation. **a** Clusters of all Nab3 cross-linking profiles generated by K-means clustering, performed using STEM^60^. Only profiles were selected that showed a maximum fold-change of at least 1.5 and had a mean pairwise correlation over two biological replicates of 0.7. The *y*-axis shows the log2-fold change of each time-point relative to time-point 0 (glucose sample). The *x*-axis shows the time-points (minutes) after the shift to medium lacking glucose that were analyzed. **b** Bar chart indicating the percentage of different RNA classes in each cluster. **c** Scatter plots comparing the Nab3 binding (*x*-axis) to Pol II transcription (*y*-axis) for the indicated time-points (minutes) after inducing glucose starvation. To compare the Nab3 and Pol II time-point 0 data we Z-normalized the FPKM values. For time-points 1 to 20 we divided the FPKM values at each time-point by the time-point zero data, which were then Z-normalized. **d** The heat map shows what fraction of the genes belonging in each Pol II K-means cluster (*y*-axis) were also found in each Nab3 cluster (*x*-axis). Dashed lines indicate groups of genes with specific Nab3 and Pol II cross-linking profiles. **e** Shown is the cumulative read density of genes belonging to groups 1 and 2 around the annotated TSS. The *black* and *gray* lines show the sense and anti-sense read densities, respectively, for the glucose data. The *red* and *blue* lines show the sense and anti-sense read densities, respectively, for the glucose-deprived cells (14 min after the shift). **f**,**g** Examples of genes (*ILV5* and *RPP0*) showing a decrease in Pol II transcription and transient cross-linking of Nab3. Biological replicates of the glucose to no glucose (*black* and *red* lines) are shown. The *blue lines* show data from glucose to glucose control experiments. *y*-axis shows fold-change relative to time-point 0

We detected differential cross-linking of Nab3 to over 4100 transcripts (~37% of all features) during glucose deprivation. Using K-means clustering, we divided the Nab3 cross-linking profiles of the differentially bound transcripts into four clusters (Fig. 5a). Interestingly, both clusters 2 and 3 show a very rapid increase in Nab3 cross-linking during the first few minutes of the adaptation response. Clusters 1 and 2 also indicated transient changes in Nab3 cross-linking during the first 8 min of glucose deprivation. These data suggest that Nab3 binding very rapidly changes during glucose deprivation and is dynamic. Cluster 0 contains transcripts that generally show a decrease in Nab3 binding. About three quarters of the transcripts in this cluster are ncRNAs (XUTs, CUTs, SUTs, anti-sense RNAs, and snoRNAs), suggesting that Nab3 binding to this class of transcripts decreases during glucose starvation (Fig. 5b). Cluster 3 genes showed an increase in Nab3 binding during the time-course. These generally were underrepresented in ncRNAs, but contained the largest group of protein-coding genes and a number of tRNAs. The vast majority of the reads mapping to the 3′ end of these tRNAs contained CCA trinucleotides, suggesting that they are mature transcripts.

We next asked how well these changes in Nab3 cross-linking correlated with changes in Pol II transcription (Fig. 5c). The Nab3 and Pol II glucose χCRAC data (*t* = 0) were highly positively correlated, which is consistent with the co-transcriptional binding of Nab3 to the nascent transcript^31, 32^. However, 1 min after the shift to medium lacking glucose, the Nab3 and Pol II data decoupled, suggesting rapid changes in Nab3 binding that were independent of alterations in Pol II transcription. Thus, during early stages of glucose deprivation, binding of Nab3 to nascent transcripts might be regulated by additional factors, providing an additional level of control, which is largely orthogonal to transcriptional regulation. At later time-points, however, the correlation between the data sets improved, indicating that the cells have started to adjust to their new environment. Consistent with this, comparison of Nab3 and Pol II χCRAC profiles revealed that at later time-points Nab3-binding profiles generally followed Pol II transcription (Fig. 5d, groups 2–3). Notably, group 1 contained many genes involved in the heat-shock response, transmembrane sugar transporters and glycolysis/gluconeogenesis. Group 2 is enriched for genes involved in the oxidative stress response and starch/sucrose metabolism, hinting at a role for Nab3 in regulating the oxidative stress response.

As many yeast promoters are intrinsically bi-directional^24, 33^, the induction of the group 1 and 2 genes during glucose starvation frequently resulted in the appearance of divergent anti-sense transcripts (Fig. 5e), which are also bound by Nab3. Two examples are *CUT075* (Fig. 5d, group 2) and *Unit247/CUT246* (Fig. 5d, group 1) that are readily detected upon the induction of the heat-shock proteins *SSA2* and *HSP78* (both group 3 genes; Supplementary Fig. 7). *Unit247/CUT246* and *CUT075* are anti-sense to *POM33* and *YAP6*, respectively. Interestingly, our Nab3-depletion data suggests that Nab3 prematurely terminates these transcripts, preventing the polymerases from reaching the 5′ ends of *POM33* and *YAP6*, which could result in silencing of these genes^15^.

For a number of the downregulated genes we observed a transient increase in Nab3 cross-linking and a reduction in Pol II transcription (Fig. 5d, group 3). Two examples are shown in Fig. 5f,g. Transcription of the *ILV5* and *RPP0* genes decreased almost linearly during glucose starvation (Fig. 5f,g, top two graphs), however, Nab3 cross-linking reproducibly increased about 2–3-fold during the first 4 to 8 min (Fig. 5f,g, top two graphs). These examples demonstrate that χCRAC can detect rapid changes in protein–RNA interactions at very high temporal resolution.

### *In cis* changes in Nab3 binding during glucose deprivation

**Fig. 6:**
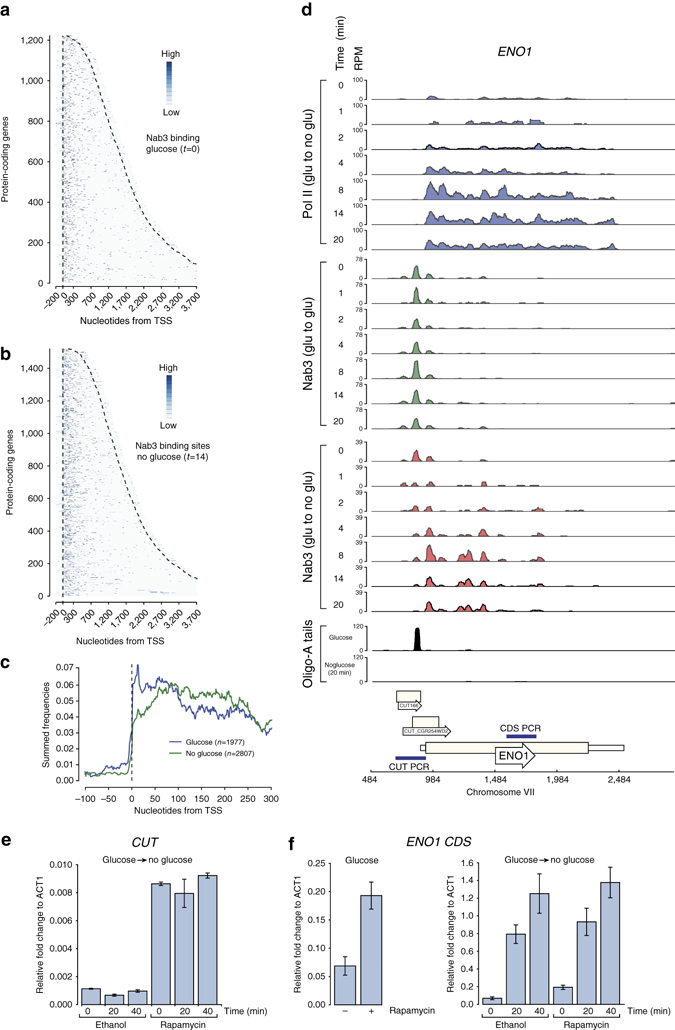
Nab3 binds to different sites in protein-coding transcripts during glucose deprivation. **a** The heat map displays the distribution of Nab3-binding sites across protein-coding genes (*y*-axis) that were aligned by the TSS (*x*-axis) and sorted by length. The *dashed lines* indicate the TSS and 3′-end, respectively. Shown is the glucose data (*t* = 0). **b** Same as in **a** but now for the *t* = 14 no glucose time-point. **c** Distribution of Nab3-binding sites around the TSS. For each Nab3 protein-coding target, the distribution frequency of the binding sites was plotted around the TSS (*x*-axis). These frequencies were subsequently summed (*y*-axis) to generate this distribution plot. The *blue line* indicates the data from the glucose experiment (*t* = 0). The *green line* shows the data from the no glucose *t* = 14 time-point. **d** Genome browser images showing the results of the Pol II control χCRAC experiment (*top panel*; *blue*), Nab3 control χCRAC experiment (*green*), the Nab3 glucose to no glucose χCRAC experiment (*red*) and the total amount of oligo-A tailed reads for the *ENO1* gene. The time-points (minutes) at which samples were harvested after shifting the cells to medium lacking glucose is indicated on the *left* side of each track. **e**,**f** qRT-PCR analyses of *ENO1* and upstream *CUT* levels. Cells were grown in glucose, treated with rapamycin or ethanol for 1 h and subsequently rapidly shifted to medium lacking glucose. RNA was extracted from cells before (0) and 20, 40 min after the shift. The qRT-PCR data were normalized to the levels of *ACT1*, as both the mRNA levels and the Pol II cross-linking profiles for this gene did not significantly change during the time-course (Supplementary Fig. 9a). *ENO1* mRNA levels were quantified using RT-PCR oligonucleotides that amplify a region that is located downstream of the main Nab3 cross-linking sites (see **d**, *bottom track*). To detect the upstream CUT in **e**, we used oligonucleotides that amplify the CUT region, including the Nab3-binding sites upstream of the *ENO1* TSS. The *left bar* in **f** shows the effect of Nab3 depletion on *ENO1* mRNA levels in cells grown in glucose. The *right bar* plot in **f** shows the results for the whole time-course. Error bars indicate s.d. from three to four experimental replicates

Nab3-binding profiles within transcripts also changed for many genes during glucose starvation (Fig. 6). As anticipated, the majority of the Nab3 cross-linking peaks identified in the glucose data (*t* = 0) clustered near the 5′end of protein-coding genes where Nab3 is known to act^31, 32, 34^ (Fig. 6a). However, 14 min after the shift to medium lacking glucose, the binding pattern of Nab3 appeared to spread more into the coding sequence (Fig. 6b). The Nab3 peak distribution plot in Fig. 6c confirmed that the Nab3-binding site distribution in the no-glucose data was significantly different from the glucose data (two sample Kolmogorov–Smirnov test; *p*-value < 10^−5^). A striking example was the enolase (*ENO1*) gene, an enzyme involved in gluconeogenesis and glycolysis (Fig. 6d), which is strongly upregulated during glucose deprivation (Fig. 6d, top panel). In the glucose to glucose Nab3 control data (Fig. 6d, Nab3 (glu to glu)) we mainly observed three Nab3 peaks in the 5′UTR of *ENO1* that overlapped with two CUTs. In the no glucose data (Fig. 6d, Nab3 glu to noglu) the main Nab3 peaks were located further downstream in the coding sequence. This transition happens very quickly: already after the first few minutes of glucose starvation we see a change in the intensity of Nab3 binding at various sites in *ENO1* (Fig. 6d).

These results demonstrate that χCRAC is also capable of detecting rapid *in cis* changes in protein–RNA interactions.

We hypothesized that this redistribution of Nab3 in *ENO1* could be linked to the use of alternative TSSs when cells are grown in glucose. Such a mechanism is sometimes employed to regulate expression of genes encoding metabolic proteins, such as *IMD2* ^35, 36^, under specific conditions. To test whether expression of *ENO1* is controlled by a similar mechanism, we analyzed Cap-binding protein (Cbp1) CRAC^37^, ChIP-Seq^38^ and TIF-Seq^39^ data to identify transcription start sites and transcript isoforms, respectively. All data sets show that transcription can initiate upstream of the *ENO1* TATA box (Supplementary Fig. 8a–c).The high ChIP signal near the CUT TSS indicates that CUT transcription is regulated by TFIID. All the available data indicate that CUT transcription is driven by a different promoter. Relative to the orthologous *ENO2*, formation of transcription initiation complexes at the *ENO1* promoter appears to be inefficient, and we speculate that this is partly the result of transcriptional interference from the upstream CUT (Supplementary Fig. 8c). Transcription of this CUT probably terminates between the *ENO1* TATA box and TSS as high levels of Nab3 cross-linking was detected in this region (Supplementary Fig. 8a). Indeed, analysis of reads containing non-encoded oligo-A tails, which are a hallmark for NNS-exosome degradation^40^, revealed many degradation intermediates that overlapped with the Nab3 cross-linking sites (Fig. 6d panel Oligo-A tails), but mainly in cells grown in glucose. Nab3 depletion resulted in a ~8-fold increase in the CUT levels, confirming that Nab3 binding triggers the degradation of the CUT. However, overall the expression levels did not change during the time-course (Fig. 6e). Relative to *ACT1*, *ENO1* mRNA levels in glucose were low and Nab3-depleted cells showed a modest increase in *ENO1* quantitative reverse transcription-PCR (qRT-PCR) signal (Fig. 6f), possibly because CUT transcripts no longer terminate at the Nab3-binding sites, and less termination in the *ENO1* coding sequence (Fig. 6f, left plot). However, Nab3 depletion did not significantly affect gene expression levels of *ENO1* during glucose starvation (Fig. 6f).

We speculate that the upstream CUT helps to suppress transcription initiation at the *ENO1* transcription start site (TSS) when cells are grown in glucose (also see Discussion and Supplementary note 2).

### Nab3 dampens the expression of stress-responsive genes

**Fig. 7:**
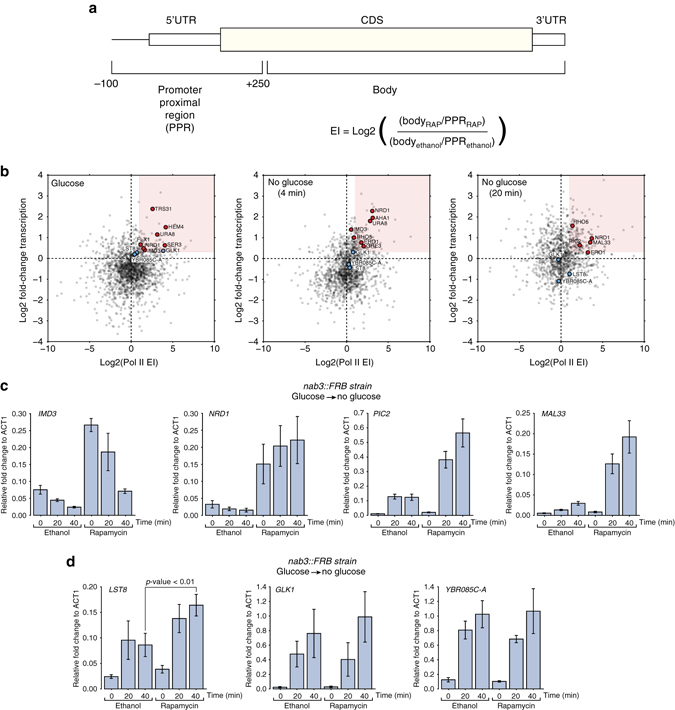
Nab3 regulates the timing of expression of stress-responsive genes. **a** Schematic representation of how escape factors (EI) were calculated. For more details, see the Methods section. **b** Nab3 targets different transcripts during glucose deprivation. The scatter plot shows the comparison of escape indices (EIs) and changes in Pol II transcription for protein-coding genes before the shift (0) and 4 and 18 min after the shift to medium lacking glucose. The *red square* indicates genes that showed at least a 1.5-fold increase in transcription and an EI of at least 2. The *red dots* indicate genes that could potentially be attenuated by Nab3. The *blue dots* indicate genes that, based on the EI, are less likely to be regulated by Nab3. **c** Quantitative RT-PCR analyses of *IMD3*, *NRD1, PIC2,* and *MAL33* transcripts during a glucose starvation experiment. Cells were grown in glucose to exponential phase, treated with rapamycin or ethanol for 1 h and subsequently rapidly shifted to medium lacking glucose (but supplemented with rapamycin). RNA was extracted from cells before (0) and 20, 40 min after the shift to medium lacking glucose. **d** Same as in **c** but now for genes that based on the calculated EI are less likely to be regulated by Nab3. Error bars indicate s.d. from three to four experimental replicates. The *p*-value was calculated using an Welch’s *t*-test on the data from the 40-min time-points

Our data analyses (Fig. 5b,d) revealed that almost a quarter of the protein-coding transcripts are differentially bound by Nab3 during glucose starvation. We hypothesized that Nab3 could play a role in regulating the kinetics of these genes during stress. To test this model, we again employed the anchor-away method to deplete Nab3 from the nucleus and asked how this globally affected Pol II transcription. To identify Nab3-regulated protein-coding genes we calculated Pol II escape indices (EI^41^; Fig. 7a) that are a measure of changes in Pol II distribution over the gene upon rapamycin treatment. We assumed that genes that are tightly controlled by Nab3 would show high read densities near Nab3-binding sites in the promoter proximal region as a result of Pol II pausing. Upon Nab3 depletion, we expected that these “pileups” would largely dissolve, leading to an increased Pol II density over the body of the gene (see example in Supplementary Fig. 6b). Thus, genes with an EI > 1 are potentially regulated by Nab3. To reduce noise, we only considered highly expressed genes with an EI > = 2 that showed at least a 1.5 increase in Pol II transcription upon rapamycin treatment (see Methods for more details; Fig. 7b, top-right red quadrant).

Five of the 14 genes selected from the glucose data (Supplementary Table 4) were previously shown to be regulated by NNS, including *SER3*, *IMD3*, *NRD1,* and *URA8* and four others (*FLX1, HEM4, TRS31,* and *SEN2*) were picked up as a result of snoRNA read-through. We identified a number of new genes that are regulated by Nab3-dependent attenuation during glucose deprivation (Fig. 7b; middle and right plot). These include the aldolase *GRE3,* the mitochondrial copper transporter *PIC2* (Fig. 5 group 2), the maltose fermentation regulatory protein *MAL33* and the small GTPase *RHO5*, all of which were upregulated during glucose starvation. Comparison of the Pol II χCRAC profiles of the Nab3-depleted and control (ethanol treated) data for these genes showed a clear accumulation of Pol II around the Nab3 cross-linking sites in the ethanol-treated cells and little transcription downstream, indicative of Pol II pausing and termination (Supplementary Fig. 9b–e). In the rapamycin-treated cells more Pol II could be detected in the body of these genes. Importantly, rapamycin treatment of the anchor-away strain expressing Nab3 without the FRB domain did not noticeably affect the Pol II transcription profiles of these genes (Supplementary Figs 6b,10), demonstrating that the observed changes in Pol II distribution is a direct result of Nab3-FRB depletion from the nucleus.

To substantiate those results, we performed qRT-PCRs on total RNA isolated from cells treated with rapamycin or the solvent (ethanol), focusing on *MAL33* and *PIC2* (Fig. 5d; group 2) (Fig. 7c). As positive controls we analyzed the levels of *IMD3* (Fig. 5d; group 3) and *NRD1*, two genes known to be regulated by Nab3-dependent attenuation^16, 23^. As negative controls we selected three genes (*LST8, GLK1,* and *YBR085C-A*; blue dots in Fig. 7b) that showed both a high increase in Nab3 binding and Pol II transcription in glucose-deprived cells (Fig. 5d groups 1 and 2), however, based on the calculated EIs were less likely to be affected by Nab3 binding. Although nuclear depletion of Nab3 resulted in only a modest increase in Pol II cross-linking of *IMD3, NRD1*, *PIC2.* and *MAL33* (generally less than twofold), total RNA levels increased quite dramatically (Fig. 7c). This suggests that in the absence of Nab3 a substantially higher number of polymerases reach the 3′ end of these genes and are terminated by the canonical cleavage and polyadenylation machinery. After 1 h of rapamycin-treatment total mRNA levels of *IMD3* and *NRD1* increased about fivefold in glucose, consistent with a role for Nab3 in terminating transcription of these genes^16, 23^. In Nab3-depleted cells, *IMD3* transcription and total RNA levels cells were generally higher throughout the time-course, suggesting that Nab3 is important for repressing *IMD3* expression in glucose and during glucose starvation (Supplementary Fig. 9b; Fig. 7c). In contrast, *PIC2* and *MAL33* mRNA levels only increased in glucose-deprived cells treated with rapamycin (Fig. 7c). Thus, *PIC2* and *MAL33* are clear examples of stress-specific Nab3 targets. Except for *LST8* (Welch’s *t*-test; *p*-value < 0.01), nuclear depletion of Nab3 did not significantly alter total mRNA levels of the control group genes under normal or stress-conditions (Fig. 7d, Supplementary Fig. 9f).

**Fig. 8:**
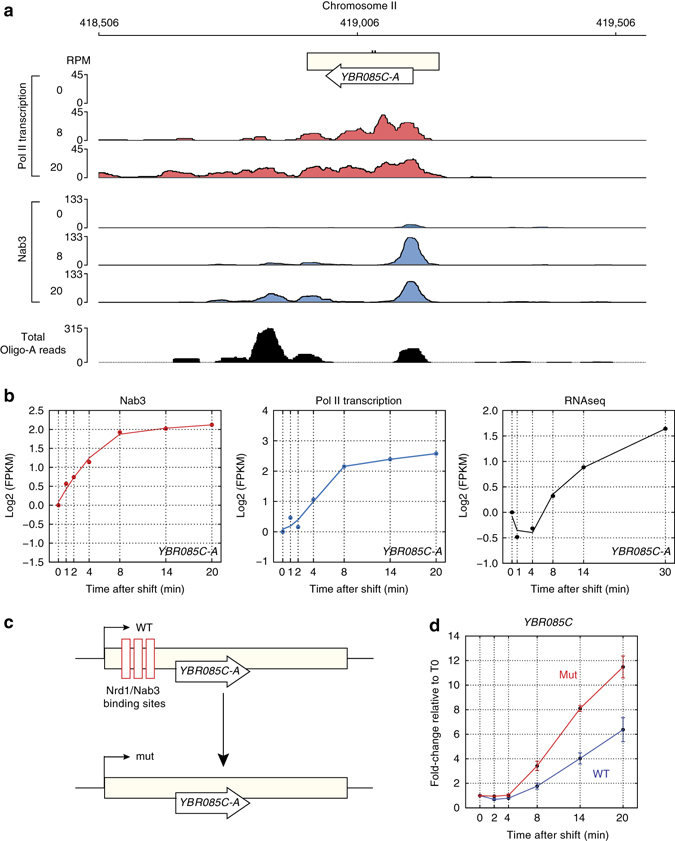
Nab3 induces changes in *YBR085C-A* expression kinetics. **a** Genome browser image showing the Pol II (*red*) and Nab3 (*green*) χCRAC data for the *YBR085C-A* region from cells harvested before (0) or 8 and 20 min after the shift to medium lacking glucose. The *bottom panel* shows the total number of reads with short oligo-A tails mapped to this region. **b** The plots show the log2-transformed FPKMs (*y*-axis) for the *YBR085C-A* transcript from the Nab3 χCRAC, Pol II χCRAC, and RNASeq data. The *x*-axis indicates the time (in minutes) after the shift to medium lacking glucose. **c** Schematic representation of how the *YBR085C-A* Nrd1-Nab3 site mutant was generated. Nrd1 and Nab3 motifs that overlapped with the main Nab3 cross-linking sites in the 5′ end of *YBR085C-A* were mutated (without changing the amino-acid sequence). **d** Quantitative RT-PCR results on total RNA isolated from the wild-type (WT) and *YBR085C-A mutant* (mut) strain during a glucose deprivation time-course. The *y*-axis shows fold change in signal relative to the 0 (glucose) sample. Error bars indicate s.d. from three to four experimental replicates

The observation that Nab3-depletion did not affect *YBR085C-A* gene expression levels was surprising given that we detected a strong increase in Nab3 cross-linking near the 5′ end of the transcript (Fig. 8a,b) and identified hundreds of oligo-A-tailed reads in the sequencing data (Fig. 8a), strongly suggesting that the NNS terminates *YBR085C-A* transcription in glucose-deprived cells. We, therefore, engineered a strain in which the Nab3 and Nrd1 motifs in the 5′ region of *YBR085C-A* were mutated (without affecting the amino-acid sequence) (Fig. 8c). Quantitative RT-PCR analyses revealed that although the difference in mRNA levels between the mutant and the wild-type gene was always less than twofold during the 20-min time-course, the mutant was upregulated faster than the wild-type gene during glucose starvation, demonstrating a role for the NNS in regulating the kinetics of *YBR085C-A* expression (Fig. 8d). Thus, we predict that changes in transcription kinetics induced by NNS-dependent termination is more widespread than the Nab3 anchor away depletion data would suggest.

We conclude that Nab3 controls the induction kinetics as well as the maximum mRNA expression levels of stress-responsive genes during glucose deprivation**.**

### Nab3 suppresses retrotransposon transcription during stress

**Fig. 9:**
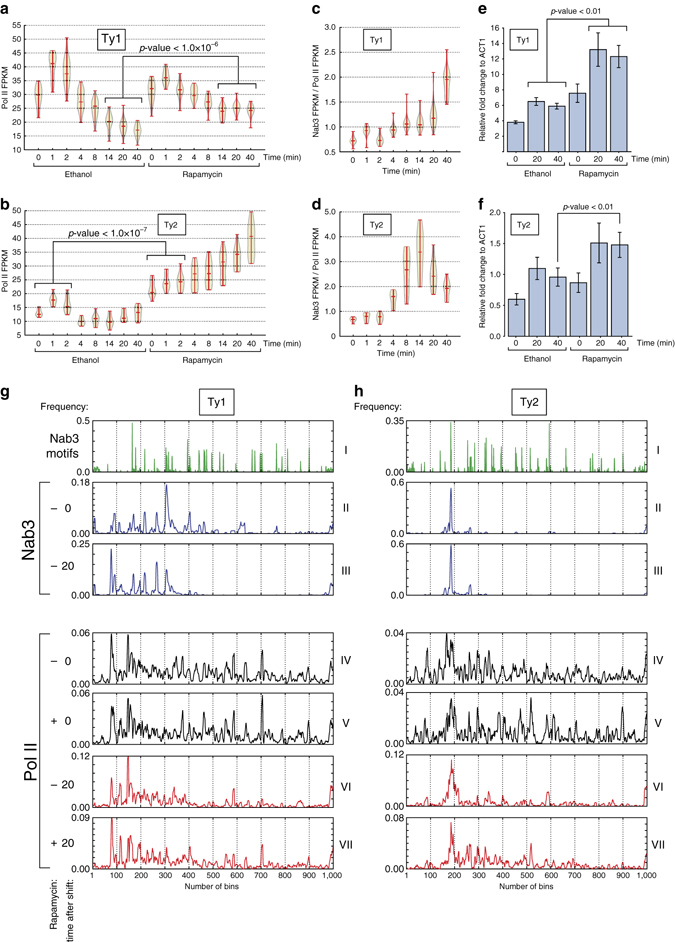
Nab3 regulates the expression of retrotransposons during glucose deprivation. **a**,**b** Violin plot showing the Pol II FPKMs for Ty1 and Ty2 retrotransposons from the *nab3::frb rpo21-HTP* χCRAC data generated in the presence of solvent (ethanol) or rapamycin. Shown are the averaged FPKMs from two biological replicates. Time (min) indicates the number of minutes in medium lacking glucose (but supplemented with rapamycin). The *p*-values were generated using Welch’s *t*-test. **c**,**d** Dynamic cross-linking of Nab3 to Ty1 and Ty2. The violin plot shows Ty1 and Ty2 FPKM distribution from a Nab3 χCRAC time-course experiment. The Nab3 χCRAC data were normalized to the average Pol II ethanol data shown in **a**,**b**. **e**,**f** Quantitative RT-PCR analysis of Ty1 and Ty2 retrotransposon transcript levels during a glucose starvation time-course. The *x*-axis shows the time (minutes) after the shift to medium lacking glucose at which samples were harvested. The *p*-values were generated using a Welch’s *t*-test. **g**,**h** Plots showing the distribution of Nab3 motifs (CUUG and UCUU; panel I), Nab3 cross-linking and Pol II cross-linking to Ty1 and Ty2 transcripts. To normalize for transcript lengths, each gene was divided into 1000 bins (*x*-axis). Roman numerals indicate the results from individual experiments. The *black plots* show the Pol II profiles for cells grown in glucose in the presence or absence of rapamycin. The *red plots* show the Pol II profiles 20 min after the shift to medium lacking glucose, in the presence or absence of rapamycin. For each Ty transcript we calculated the fraction of reads that mapped to each bin for each individual transcript. These were subsequently summed (*y*-axis) to generate these profiles

We showed that prolonged UV-exposure substantially increased Nab3 cross-linking to Ty retrotransposon transcripts (Fig. 2b,e, and f). To investigate whether Nab3 regulates Ty retrotransposon expression, we measured their transcription and total RNA levels in the Nab3 anchor-away strains treated with ethanol or rapamycin. Yeast expresses five different classes of Ty retrotransposons (Ty1 to Ty5)^42^. In line with transposon-abundance, very few reads mapped to Ty5, which was, therefore, not further considered. Consistent with our initial results (Fig. 4b), in the ethanol-treated cells we observed a transient increase in Pol II cross-linking to the highly abundant Ty1 and Ty2 retrotransposons (Fig. 9a,b). Rapamycin treatment did not affect Ty1 transcription kinetics during the first 8 min, however, at later time-points Pol II transcription was significantly higher (Fig. 9a; Welch’s *t*-test; *p*-value < 1.0×10^−6^). This suggests that Nab3 activity is required to suppress transcription of Ty1 transposable elements primarily during late stages of the glucose adaptation response. Consistent with this idea, Nab3 cross-linking to Ty1 was highest at the late time-points (Fig. 9c). Remarkably, Nab3 appears to control transcription of Ty2 retrotransposons more tightly; In Nab3-depleted cells Ty2 transcription was significantly higher in glucose medium (Welch’s *t*-test; *p*-value < 1.0×10^−7^) and continued to increase during the glucose deprivation response (Fig. 9b). Nab3 cross-linking to Ty2 transcripts was dynamic, peaking at 14 min after the medium shift (Fig. 9d). These data demonstrate how χCRAC can be used to measure alterations in Pol II transcription kinetics during changes in the environment or in mutant strains.

For the less-abundant Ty3 and Ty4 retrotransposons the pattern was noisy, however, we could detect an increase in Ty3 Pol II transcription during the last three time-points, indicating that Nab3 is also involved in regulating Ty3 expression (Supplementary Fig. 11). Quantitative RT-PCR analyses confirmed that Ty1 and Ty2 total RNA levels increased during the time-course (Fig. 9e,f), most significantly (Welch’s *t*-test; *p*-values < 0.01) at late stages of the adaptation response.

To ascertain why Nab3 appears to more tightly regulate Ty2 transcription, we next compared the Nab3 and Pol II cross-linking profiles over Ty1 and Ty2 retrotransposon genes in the Nab3-FRB anchor-away strain grown in glucose (*t* = 0) or deprived of glucose for 20 min, with and without rapamycin treatment (Fig. 9g,h). To normalize for the differences in Ty transcript lengths, we divided the reads over an equal number of bins (Fig. 9g,h, *x*-axis). Strikingly, Nab3 cross-linked primarily to a single region in Ty2 retrotransposons (Fig. 9h, panel II), whereas Nab3 cross-linking over Ty1 transcripts was more diffuse (Fig. 9g, panel II). Although Nab3 cross-linking to Ty1 and Ty2 transcripts increased over time during glucose deprivation (Fig. 9c,d), the cross-linking pattern did not dramatically change (Fig. 9g,h, compare panels II and III). Ty1 and Ty2 Pol II cross-linking profiles in cells grown in glucose were very similar, with the read densities roughly evenly distributed over the genes. Rapamycin treatment of cells in glucose only modestly increased the read density downstream of the main Nab3 peaks (Ty1 EI = 0.15; Ty2 EI = 0.37; Fig. 9g,h, compare panels IV and V). These data suggest that only a small fraction of Ty1 and Ty2 transcripts is terminated by Nab3 in glucose. However, 20 min after the shift to medium lacking glucose, Pol II cross-linking downstream of the Nab3 sites in both Ty1 and (in particular) Ty2 transcripts was substantially reduced (Fig. 9g,h, panel VI). Nab3 depletion by rapamycin treatment did not completely resolve the Pol II pileups near the Nab3 cross-linking sites (Fig. 9h, compare panels VI with VII), however, still a much higher fraction of reads was detected in downstream regions (Ty1 EI = 0.47, Ty2 EI = 1.0). These data support the notion that Nab3-dependent transcription termination is mostly active on retrotransposons during glucose starvation.

Collectively, these results demonstrate that Nab3 plays an important role in regulating the kinetics of retrotransposon gene expression during glucose deprivation.

## Discussion

The methodological advances underpinning the development of χCRAC enabled us to glimpse the highly dynamic reprogramming of RBP–RNA interactions in response to stress. Our data reproducibly show widespread relocation of the yeast transcriptional termination factor Nab3 within a minute of the imposition of stress. Given the central role of RBPs in all aspects of RNA life, it is plausible that other RBPs show similar dynamic behaviors. Because χCRAC is generally applicable we anticipate that its use will enable future studies to unearth novel mechanistic insights into the function of RBPs during stress conditions.

**Fig. 10:**
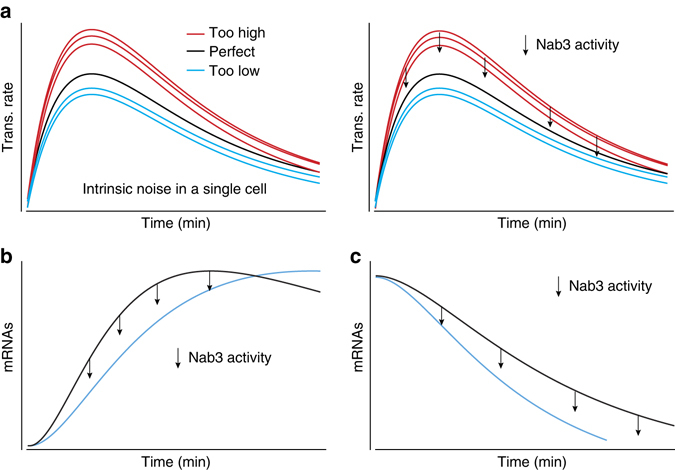
Models for how Nab3 could contribute to regulating gene expression during stress. **a** Shown is a schematic representation of typical gene expression profiles observed during stress responses. The *black lines* in the plots indicate the ideal gene expression profile. The *red* and *cyan* lines indicate variability in gene expression (either too high or too low). Here, Nab3-dependent transcription termination may function to prevent transcription from over-shooting. **b**,**c** Nab3 activity could also contribute to stress adaptation by either dampening the expression of a gene **b**, which would increase the response time, or its termination activity could contribute to rapidly shutting down expression of genes that are downregulated during stress **c**

Our results provide evidence that Nab3 “dampens” the induction of several stress-responsive genes during stress. What could be the benefit of this? One possibility is that Nab3 functions as part of a negative feedback loop that reduces noise in gene expression by preventing transcription levels from overshooting (Fig. 10a). Nab3 could also regulate the timing of expression of these stress-responsive genes, although both models are not mutually exclusive. Many of these stress-responsive genes are controlled by the same group of transcription factors. Although the benefits of expressing many stress-responsive genes simultaneously undoubtedly has advantages, in some cases it might be required that expression of certain genes is delayed (Fig. 10b) or only strongly induced when the stress signal has reached a certain amplitude. Such a dampening system would also reduce activation of gene expression due to false or noisy signals. In this respect, the role of Nab3 might be similar to that of the Set3c histone deacetylase, although the mechanism is different^43^. Deletion of Set3c induces the expression of certain genes much faster when cells are subjected to changes in carbon sources and, therefore, it was proposed that Set3c dictates the expression timing of these genes^43^.

We also uncovered a role of Nab3 in regulating the expression kinetics of Ty retrotransposons, which are upregulated during a variety of stress conditions^44^. Their expression needs to be carefully controlled as recombination between Ty elements can lead to chromosomal rearrangements, which are detrimental for gene expression and genome stability^45^. In Nab3-depleted cells, transcription of Ty1 and Ty2, in particular, is significantly upregulated at later stages of the glucose deprivation response and we observed a transient increase of Nab3 binding to Ty2 retrotransposons at late stages of the adaptation response. We propose that Nab3 activity contributes to stress adaptation by rapidly shutting down transcription of retrotransposons (Fig. 10c).

In many fungi, as in mammals, retrotransposon transcription is regulated by the RNAi machinery^46, 47^. *S. cerevisiae*, however, does not have RNAi components, and it is tempting to speculate that, in view of its poor conservation, the NNS complex, together with other factors, may have taken up the function of controlling the expression of retrotransposons.

We show that χCRAC can also measure rapid *in cis* changes in protein–RNA interactions *in vivo*. We demonstrate that Nab3 binding to the *ENO1* transcript changes within the first few minutes of the glucose starvation response (Fig. 6). Follow-up analyses revealed that Nab3 binds a CUT that initiates upstream of the *ENO1* TATA box and we predict that transcription of this CUT upstream of the *ENO1* promoter helps to suppress *ENO1* expression when cells are grown in glucose. Deciphering the mechanism of regulation of *ENO1* expression is not trivial as this gene is controlled by many different transcription factors (See Supplementary note 2 and Supplementary Fig. 12). However, it is worth mentioning here that the upstream CUT initiates from an element referred to as the upstream repressor element (URS), which is a binding site for many transcription factors, such as Reb1 and Tye7 (Sgc1), and mediates ~20-fold repression of *ENO1* in glucose^48^. Interestingly, this URS has directionality as reverting this element relieves inhibition of *ENO1* expression when cells are grown on glucose^49^ (Supplementary Fig. 12). This begs the question whether transcription of the CUT is also reversed in this mutant.

There are many biological scenarios where measurements of *in cis* changes, as observed in *ENO1*, could shed light on RBP–RNA interaction dynamics. A major area of interest is the assembly of large macromolecular RNP complexes, such as the ribosome and the spliceosome, which involves dynamic interactions between many proteins and RNAs. It is likely that some assembly factors contact different sites on their RNA substrates or may not occupy all of their binding sites simultaneously during the assembly process, as is the case for ribosomal proteins during ribosome assembly^50^. We envision that χCRAC could be used to perform high-resolution time-resolved analyses of dynamic changes of protein–RNA interactions in RNP particles during their assembly in vivo. Such studies would require the development of protocols to synchronize the cells in a way that the assembly could be monitored from start to finish.

Another major potential area of application is the study of the kinetics of RNA expression, which is a balance of RNA transcription and degradation. Most studies use indirect methods to estimate RNA decay rates, which can rely on Pol II mutants, metabolic labeling or drugs to inactivate transcription^51, 52^. Although these studies have provided a wealth of interesting results, the data generated by these indirect approaches are not always highly correlated^53^. In general, model-based studies assume that RNA decay can be summarized by a single mRNA half-life for each transcript, corresponding to a simple exponential decay process^52, 54^, despite the complexity of RNA degradation pathways^55^. The highly dynamic behavior of the termination factor Nab3 indeed challenges this assumption. Since RNA degradation involves the activity of many nucleases, dissecting the dynamics of individual proteins is likely to be crucial for understanding how the rate of RNA decay is determined. We envision that χCRAC analyses on individual nucleases would enable us to directly measure such interactions, providing invaluable data to constrain and refine our understanding of the kinetics of gene expression.

## Methods

### The Vari-X-linker

The Vari-X-linker incorporates a number of new features that enhance the effectiveness of UV cross-linking. The sample is presented in a controlled 1 cm thick layer contained in a specially constructed UV transparent bag and flanked by two beds of powerful 254 nm (400–550 W) or 365 nm lamps (350 W) that were assembled on trays for easy exchange of the lamps. As far as we are aware, the Megatron is currently the fastest cross-linker available on the market for cross-linking proteins to RNA in actively growing cells^12^. Despite this, it still requires about 100 s to get good cross-linking yields with this machine (or more depending on the protein), which is not fast enough to do time-resolved analyses with minute time-point resolution. Another problem we faced with the Megatron system is that it was not trivial to cross-link cells when the lamps were at full output. As a consequence, cells would not always receive the same level of 254 nm UV intensity during the 100 s of UV-irradiation, resulting in variation in cross-linking efficiencies between samples and noise in the data. It was also not possible to control the temperature inside of the unit. Although the Megatron works well for normal cross-linking studies, these issues made it very difficult to perform time-resolved analyses during short periods. To overcome this, we incorporated a shutter system to allow the lamps to be at full power with stable, repeatable output before exposing the cells. A fan cooling system was installed to minimize thermal shock to the sample. With the Vari-X-linker, the lamps can be left on throughout the experiment and cells will only be exposed when the shutters are opened (Supplementary Fig. 1; shutter release). Using a vacuum pump the cells can be quickly extracted from the UV chamber. The bag in the UV-chamber can be exchanged for a tray that allows for the cross-linking of small volumes or adherent cells in petri-dishes. Cross-linking of adherent cells could be improved by growing the cells on UV-transparent plastic. Using a vacuum pump the cells can be quickly extracted from the UV chamber. We also developed a new filtration device that enables harvesting of 1 L of cells in ~30 s. The Vari-X-linker and the filtration device can be purchased from UVO_3_ (www.vari-x-link.com; sales@uvo3.co.uk).

### Kinetic CRAC (χCRAC)

For an eight time-points time-course, 8 L of cells were grown in synthetic medium with glucose (SD-TRP) to exponential phase (OD_600_~0.5) at 30 °C. For time-point zero, 1 L of cells were cross-linked in the Vari-X-linker using the high-output 254 nm lamps for 12 s and then harvested by rapidly passing the cells through a 0.8 µm filter (Millipore) using a new vacuum filtration device (see above). The remaining 7 L of cells were harvested on filters and quickly resuspended in S-TRP (no glucose samples) or SD-TRP (glucose control samples) and maintained at 30 °C. For each time-point 1 L of cells were cross-linked in the Vari-X-linker and harvested by filtration as above. This yielded ~1 g of cells for each time-point.

For the PAR-CLIP experiments (Fig. 1c), for each condition 1 L of cells expressing Nab3-HTP were grown to exponential phase in SD-URA-TRP and incubated with 4-thio-Uracil for 5 min (final concentration = 20 µM). After labeling, the cells were rapidly harvested by filtration onto 0.8 µm membranes to remove the free 4-thio-Uracil and resuspended in SD-TRP before UV-irradiation at 365 nm. Removing the free 4-thio-Uracil greatly enhanced the cross-linking efficiency (data not shown).

Cells were lysed in 1 V/w of TN150 (50 mM Tris pH 8.0, 150 mM NaCl, 0.1% NP-40, 5 mM β-mercaptoethanol) and 3 V of Zirkonia beads (0.5 mm; Thistle Scientific) by vortexing the cells five times for 1 min, with a 1-min incubation on ice between each step. Three milliliter of lysis buffer was added and extracts were clarified by centrifugation (20 min at 4500×*g* and 20 min at 20,000×*g* at 4 °C). Extracts were incubated with 250 µl of equilibrated IgG Sepharose beads (GE Healthcare) for 2 h at 4 °C. Beads were washed three times 5 min with 10 ml of TN1000 (50 mM Tris pH 7.5, 0.1% Nonidet P-40, 5 mM β-mercaptoethanol, 1 M NaCl) and three times 5 min with TN150 (50 mM Tris pH 7.5, 0.1% Nonidet P-40, 5 mM β-mercaptoethanol, 150 mMNaCl).

For the Nab3 anchor-away nuclear depletion experiments^29^, cells were incubated with 1 µg/ml rapamycin (Sigma) for 1 h before the cells were shifted to medium lacking glucose (but supplemented with 1 µg/µl rapamycin).

For the Pol II (Rpo21-HTP) χCRAC experiments, cells were lysed in 1 V/w TMn150 (50 mM Tris-HCl pH 8.0, 10 mM MnCl_2_, 0.1% NP-40, 5 mM β-mercaptoethanol, 150 mM NaCl, Roche Midi protease inhibitors; 1 ml per gram of cells). Subsequently, 1 V/w of TMn150 was added containing 1U/ml of RQ1 RNase-free DNAse (Promega) and the suspension was incubated for half an hour on ice to degrade the chromatin.

For the Hfq cross-linking test (Fig. 1b), 0.5 L of bacteria were grown in Luria-Bertani medium (LB) to an OD_600_ of 0.4 and cross-linked in the Megatron and Vari-X-linker for the indicated times. To purify the *E. coli* Hfq-HTF, one gram of cells was lysed as described above. Extracts were incubated with 35 µl of anti-Flag magnetic beads (Sigma) for 2 h at 4 °C. Beads were washed three times 10 min with TN1000 and rinsed three times with TN150.

For the TEV cleavage step, beads (IgG or Flag) were resuspended in 600 µl of TN150 and incubated for 2 h with 10 µg of home-made GST-TEV protease at 18 °C. The TEV eluates were subsequently incubated with 0.1 unit of RNace-IT (Agilent) for 5 min at 37 °C after which 0.4 g of guanidine HCl (Sigma) was added to the TEV eluates to inactivate the RNAses. NaCl and Imidazole was added to a final concentration of 300 and 10 mM, respectively and the samples were incubated overnight with 50 µl of Nickel agarose beads (Qiagen) at 4 °C. Beads were transferred to a Snap Cap columns (Pierce), washed three times with 500 µl wash buffer I (50 mM Tris-pH 7.5, 6 M guanidium-HCl, 0.1% Nonidet P-40, 5 mM β-mercaptoethanol, 300 mM NaCl, 10 mM Imidazole) and three times with 1×PNK buffer (50 mM Tris pH 7.5, 0.1% Nonidet P-40, 5 mM β-mercaptoethanol, 10 mM MgCl_2_). Beads were subsequently incubated with 80 µl of 1xPNK buffer containing eight units of TSAP alkaline phosphatase (Promega) and 80 units of recombinant RNasin (Promega) for 1 h at 37 °C. After one 500 µl wash with wash buffer I and three 500 µl washes with 1xPNK buffer, the beads were resuspended in 80 µl of 3′ linker ligation mix (1xPNK buffer, App-PE 3′ adapter (see Supplementary Table 2; 0.6 µM final concentration), 10% PEG8000, 30 units of T4 RNA ligase 2 truncated K227Q (NEB), 60 units RNAsin (Promega)). The samples were incubated at 25 °C for 4–6 h. Following one 500 µl wash buffer I wash and three 1xPNK buffer washes, the beads were incubated with 60 µl of 5′end labeling mix (1xPNK buffer, 30µCi ^32^P-γATP (Perkin Elmer) and 30 units of T4 polynucleotide kinase (NEB)) for 40 min at 37 °C. ATP (Roche) was added to 1 mM final concentration, followed by another 20-min incubation at 37 °C. Beads were subsequently washed three times with 500 µl of wash buffer I and three times with 500 µl of 1xPNK buffer and incubated with 80 µl of 5′ linker ligation mix (1xPNK buffer, 10 mM ATP, 80 units RNAsin (Promega) 40 units of T4 RNA ligase 1 (NEB) and 5′ adapter (1.25 µM final concentration; see Supplementary Table 2) overnight at 16 °C. Beads were subsequently washed three times with 500 µl wash buffer I and three times with 500 µl of wash buffer II (50 mM Tris pH 7.5, 0.1% Nonidet P-40, 5 mM β-mercaptoethanol, 50 mM NaCl, 10 mM Imidazole). Proteins were eluted from the nickel beads using wash buffer II containing 250 mM Imidazole, TCA precipitated (20% final concentration) and resolved on 1 mm thick 4–12% NuPAGE gels (Thermo Fisher Scientific), transferred to nitrocellulose membranes and visualized by autoradiography. Bands corresponding to the size of the protein of interest, including a region ~ 1 cm above the band, were cut from the nitrocellulose membrane and pooled in a single 2 ml tube. Radiolabeled RNA was extracted by incubating the membrane slices with 200 µg of proteinase K in 800 µl of wash buffer II containing 1% SDS and 5 mM EDTA. The solution was transferred to a new tube and the RNA was subsequently phenol-chloroform-extracted and ethanol-precipitated. Reverse transcription with SuperScript III was performed as per the manufacturer’s procedures (Thermo Fisher Scientific) using the reverse transcription primer listed in Supplementary Table 2. The cDNAs were purified using the Zymo DNA Clean & Concentrator 5 kit and eluted into a final volume of 10 µl. Five microliter of cDNA was PCR amplified using Pfu polymerase (Promega) for 20–24 cycles (95 °C 30 s, 52 °C 30 s and 72 °C 1 min) using PCR oligonucleotides listed in Supplementary Table 2. PCR products were resolved on 2% Metaphor agarose gels (Lonza) and 160–300 bp fragments were gel purified using the miniElute kit (Qiagen) according to the manufacturer’s procedures. Paired-end sequencing (50 bp) was performed by Edinburgh Genomics using the IlluminaHiSeq 2500 and 4000 platforms. This improved the detection of high-confidence cross-linking induced mutations^6^. Following sequencing, samples were demultiplexed using the 5′ adapter barcode sequences and collapsed reads were mapped to the yeast genome. To improve T4 RNA ligation efficiencies, we added random nucleotides to adapter termini that ligate to the RNA (Supplementary Table 2). We found that there is a significant preference for specific donor–acceptor nucleotide combinations for both 5′ and 3′ T4 RNA ligase reactions (Supplementary Fig. 3).

The uncropped images for Figs. 1 and 3b are provided in Supplementary Figs. 13 and 14.

Procedures used for western, northern, qRT-PCR and a description of the yeast strains and media can be found in the Supplementary Methods.

### Processing of raw sequencing data

Sequencing was performed on IlluminaHiSeq 2500 and HiSeq 4000 machine by our Edinburgh Genomics facility. The complete pipeline for the processing of paired-end kinetic CRAC data is available on https://bitbucket.org/sgrann/kinetic_crac_pipeline. The entire pipeline can be run using a single script (CRAC_pipeline_PE.py) that divides the tasks over multiple processors. The pipeline performs the following steps: demultiplexing of raw fastq files by pyBarcodeFilter.py version 2.3.3 from the pyCRAC tool suite^56^ (version 1.2.2.6). Flexbar^57^ then trims the reads and removes 3′ adapters sequences (Supplementary Table 2). Reads are then collapsed using random barcode information provided in the in-read barcodes using the pyCRAC tool pyFastqDupicateRemover.py (see Supplementary Table 2 for adapter sequences). Reads are then aligned to the reference sequence (yeast genome R64 in our case) using novoalign (www.novocraft.com) version 2.0.7 and those that mapped to multiple genomic regions were randomly distributed over each possible location. PyReadCounters then makes read count and fragments per kilobase transcript per million reads (FPKM) tables for each annotated genomic features. Only genes for which cross-linking could be detected in all time-points were considered. Genomic feature files were obtained from ENSEMBL (version R64-1-1.75). Coordinates for anti-sense transcripts, CUTs, XUTs, SUTs, and retrotransposons^24, 33, 58, 59^ were obtained from the Saccharomyces Genome Database (sgd; yeastgenome.org).

### Identification of Nab3-binding sites and oligo-A reads

PyCalculateFDRs.py was used to find significantly enriched Nab3-binding peaks using default settings. Only peaks with at least five reads were considered and the minimum width of the peak interval was set to 20 nucleotides. Oligo-A reads were identified using blast and in-house perl and python scripts. PyBinCollector.py was used to generated the Nab3 and Pol II cross-linking distribution figures.

### K-means clustering

K-means clustering of cross-linking profiles was performed using the STEM clustering program^60^. Only profiles were selected that showed a fold-change in FPKM of at least 1.5 and had a mean pairwise correlation over two biological replicates of 0.7.

### Escape indices and selection of Nab3 attenuated genes

To calculate the Pol II transcription EI^41^, we first selected protein-coding genes that had a minimum coverage of 10 FPKM at the indicated time-points (0, 4 and 18 min after the induction of the glucose deprivation response). The EI was calculated by summing the nucleotide density in the promoter proximal region (PPR; −100 to + 250 from the 5′UTR) and dividing this number by the nucleotide density of the body region (+251 to 3′ end). Subsequently, we divided the values for the 4 and 18-min time-point by the values for the 0 time-point to calculate the EI. These data were then compared to changes in Pol II transcription, which was calculated by dividing the total normalized nucleotide density of the whole gene from the 18-min sample by the total normalized nucleotide density of the 0-minute sample. We then only selected those genes that showed an increase in transcription of at least 1.5, a coverage of at least 10 FPKM and EI of at least 2. From the resulting list of genes, only genes were selected that (a) had Nab3-binding sites near the 5′ end of the gene and (b) showed reproducible profiles in a replicate experiment.

### Data normalization

We scaled the FPKM values of all transcripts within each time point by a constant factor such that the sum of FPKMs for all time points and all experimental replicates is the same. This was done for all data sets that were analyzed simultaneously, e.g., Nab3 and Pol II data sets (see below). For all data sets, the same time points were used (on a few occasions, temporally close time points were deemed identical for experimental purposes). Finally, for all analyzed data sets, we divided each time series of each experimental replicate by its steady state value before the imposition of stress (at 0 min after the nutrient shift). This way, all time series start at the same normalized binding value of 1 a.u. before the nutrient shift and the other values for later time points are relative to the background binding signal. We only keep those transcripts for the analysis that have real values for all time points in all experimental replicates after all steps of the normalization procedure.

### Differential gene expression analyses

For the differential expression analyses we used DESeq2^22^ in which two Megatron data sets were compared to four Vari-X-linker Nab3 glucose data sets. Only differentially expressed genes were selected that had an adjusted *p*-value of 0.05 or lower.

### Testing for differential dynamic response

To determine whether the imposition of stress results in differential dynamics of RBP binding, we used a Bayesian non-parametric regression approach. Let *f*_*j*_(*t*) represent the binding response in condition *j* (stress or control) at time *t*, relative to time 0. Our main assumption is that this response, averaged over a population of cells, can be well modeled as a smooth function of time. To capture this assumption, we formulate a probabilistic model for the response function in terms of GPs (see e.g.^27^). A GP is an infinite-dimensional generalization of the multivariate Gaussian distribution, which provides a suitable prior distribution over a space of functions. Here we enforce the smoothness assumption by modeling correlations between function values at times *s* and *t* using a squared-exponential covariance function:

$${\rm{cov}}({f}_{j}(s),\,{f}_{j}(t))={a}^{2}\exp \left[-\frac{{(s-t)}^{2}}{2{\lambda }^{2}}\right]$$

This covariance depends on two hyperparameters *α, λ* that are fitted to the data as described below.

We assume that observations *y*_*j*_(*t*) of binding to a specific transcript in condition *j* at time *t* (i.e., FPKM from the CRAC experiment at time *t* in condition *j*) are obtained from the unobserved function *f*_*j*_(*t*) by addition of zero-mean Gaussian noise with standard deviation *σ*. These assumptions enable us to marginalize exactly the unobserved function values to obtain an estimate of the data evidence (or marginal likelihood).

$$p(y(0),\ldots ,y(T)|\alpha ,\lambda ,\sigma )$$

We then reformulate the testing question as a model selection problem. We consider two competing models:

- H0, all the time series (control and stress) can be explained as noise corrupted observations of a single underlying function *f*(*t*) describing the dynamics of the system (null hypothesis).
- H1, control and stress time series result from two distinct underlying dynamics, i.e., there are two functions *f*_c_(*t*) and *f*_*t*_(*t*), which generate the observations (alternative).

The ratio of the evidence under the two hypotheses (Bayes factor) quantifies the ratio of posterior probabilities of each model being correct, and hence provides a criterion for selecting one hypothesis over the other. We follow Kass and Raftery^28^ in adopting a Bayes factor greater or equal to 10 as strong evidence of one hypothesis over the other. The Bayes factor computation is performed independently for every transcript; it should be noted that, as this is a Bayesian method, no issues of multiple testing arise, since sampling variability is already accounted for in the marginalization process.

It should be noted that the evidence calculation can only be performed exactly when the covariance hyperparameters *α*, *λ* as well as the observation noise with standard deviation *σ* are known. Such parameters can also be assigned a prior distribution and marginalized for a fully Bayesian treatment; however, this greatly complicates the computational task of computing the evidence as exact marginalization is not possible. To avoid these additional overheads, we adopted an empirical Bayesian strategy and fixed the hyperparameters in a data-driven fashion. The scaling hyperparameter*α* ^2^ was defined as 50% of the variance of all binding time series, in control and treatment experimental replicates, for a given transcript. The length scale hyperparameter *λ*, which determines the number of units the data can be extrapolated away from, was globally set as 1 min. The variance of the observation noise *σ* ^2^ was chosen to be the variance of the binding time series of a given transcript in control conditions, i.e., under the glucose-to-glucose shift. Notice that the GP models under both hypotheses were provided with the same hyperparameter values, to avoid over fitting to the data.

### Code availability

The pyCRAC package^56^ used for the data analyses is available on https://bitbucket.org/sgrann/pycrac. The complete data analysis pipeline is available on https://bitbucket.org/sgrann/kinetic_crac_pipeline/. Other python and perl scripts used for isolating oligo-A tailed reads are available upon request.

### Data availability

Fastq and processed sequencing data that support the findings of this study have been deposited in the Gene Expression Omnibus (GEO) under the accession code GSE85545 (https://www.ncbi.nlm.nih.gov/geo/query/acc.cgi?&acc=GSE85545). The data that support the findings of this study are available from the corresponding author upon request.

## Electronic supplementary material

Supplementary Information Supplementary Figures, Supplementary Tables, Supplementary Notes, Supplementary Methods and Supplementary References

Peer Review File Reviewer reports and authors' response from the peer review of this Article at Nature Communications
