# Supplementary material for: Kinetic CRAC uncovers a role for Nab3 in determining gene expression profiles during stress
Source: Nat Commun. 2017 Apr 11;8:12. doi: 10.1038/s41467-017-00025-5 (PMC5432031; doi:10.1038/s41467-017-00025-5)
Supplement: Supplementary file 1 — Supplementary Figures, Supplementary Tables, Supplementary Notes, Supplementary Methods and Supplementary References [file 41467_2017_25_MOESM1_ESM.pdf]

Supplementary note 1

Rapid nuclear depletion of Nab3 using the anchor-away system

To rapidly deplete Nab3 from the nucleus we used the anchor-away system. Nab3 was fused to the FKBP12 rapamycin-binding domain (FRB) and depleted from the nucleus by rapamycin treatment, which forms a very stable complex with Nab3-FRB and the nuclear r-protein RPL13A-FKBP12 fusion associated with ribosome assembly intermediates, which are exported to the cytoplasm. Strains expressing Nab3-FRB grew comparable to the parental strain, but failed to grow in the presence of rapamycin (Supplementary Fig. 5a) A one-hour rapamycin treatment was sufficient to detect 3'-extended snR13 species, which are known to accumulate in NNS mutants[2] (Supplementary Fig. 5b). To measure changes in transcription profiles, we performed Pol II χCRAC on Nab3 depleted cells. This revealed an accumulation of 3'-extended CUTS and snoRNAs, both under normal (glucose) and stress (no glucose) conditions (Supplementary Fig. 6a; Nab3-FRB data). Importantly, when we incubated the parental strain with rapamycin for one hour, we did not observe these changes in snoRNA and CUT transcriptional profiles (Supplementary Fig. 6a; Nab3 data). *NRD1* transcript levels are auto-regulated by NNS-dependent attenuation of transcription[3]. In the rapamycin treated *nab3::FRB* cells, we observed a higher density of Pol II downstream of the Nab3 binding sites, suggesting NNS-dependent attenuation of *NRD1* is strongly diminished (Supplementary Fig. 6b; Nab3-FRB data). Increased Pol II transcription could also be detected downstream of the *snR13* gene in the Nab3 depleted cells, consistent with a defect in Nab3-dependent transcription termination (Supplementary Fig. 6c). Again, these changes in Pol II distribution were not observed in the rapamycin treated parental

strain (Nab3 panels), demonstrating that these changes are a direct result of Nab3 depletion.

We conclude that with our Nab3 depletion conditions we can faithfully reproduce previously published work.

Supplementary note 2

Nab3 and regulation of *ENO1* transcription initiation

As shown in a schematic overview of *ENO1* transcription-regulation (not to scale; Supplementary Fig. 12), two CUTs are detected upstream of the gene, which is activated by two upstream activating sequences (UAS1, UAS2) that bind multiple factors[4-6]. *ENO1* also contains an upstream repressor sequence element (URS) in the promoter, located between -226 and -125 bp from the TSS. This URS has a directionality as reverting this element relieves inhibition of *ENO1* expression when cells are grown on glucose[7]. Experimental evidence has been published for three URS-binding factors: i) Reb1, that associates with a region not essential for, but enhancing URS function[8] and binds the UAS2 as well[4,8]. Deletion of Reb1 had no major effect on *ENO1* expression[9,10]; ii) the BUF-complex, consisting of Rfa1 and Rfa2 – which participate in yeast DNA replication as ssDNA binding proteins – that recognizes the repressor-of-CAR1-like sequence (TaGCCaCCTC) at the 5' end of the region essential for URS activity[11,12]. Repression is possibly mediated by Ume6[13], which recruits histone deacetylase Rpd3p and chromatin-remodeling factor Isw2p[14]. Long-range DNA-looping via Ume6 is associated with transcriptional repression[15]. Although Ume6 had no influence, Isw2 was found to downregulate *ENO1* expression[9,10] and can repress

transcription of cryptic RNAs[16]. iii) The basic-domain helix-loop-helix (bHLH) protein Sgc1 (Tye7) binds to the E-box motif (CAnnTG) in the 3' region of the URS[17], possibly as a (hetero) dimer[18], and, depending on surrounding sequences, could bend DNA[19]. Sgc1, like Gcr1, Gcr2 and Rap1, down-regulates *ENO1*[9,10]. In cells expressing the dominant *SGC1-1* allele (E→Q, ten residues upstream of the bHLH DNA-binding/dimerization domain), *ENO1* mRNA levels become comparable to those of *ENO2* in wild-type cells[20] during growth in glucose.

As shown in Supplementary Fig. 8c and Fig. 6d, we observed increased Nab3 binding to RNAs overlapping the region spanning the TATA-box, a long pyrimidine-rich region including three Nab3 binding motifs (UCUU; CUUG) and up to the TSS when cells were grown on glucose, but significantly less so after removal of the carbon source. These products increased in number after depletion of Nab3 (Fig. 6e) and carry poly-A tails (Fig. 6d), indicative for poly-adenylation by the TRAMP-complex and needed for degradation by the exosome. The 5'UTR-derived products also overlap the two CUTs, that are only observed in the absence of exosome components[21,22]. *CUT166*, which initiates from the 5' end of the URS has also been detected by CRAC using Cbc1, a component of the cap-binding complex[23] and by transcript isoform sequencing (TIFseq)[24] (Supplementary Fig. 8).

The role of the URS and the associated protein factors suggest that these are the primary regulators of *ENO1* transcription by controlling productive transcription initiation from its TSS, ~40 nt upstream of the ATG. They appear to do this (possibly indirectly), by stimulation of *CUT166* transcription, but not by promoting alternative TSS choice. For cells growing on glucose we did not observe (also after depleting Nab3) a marked

change in the distribution of Pol II; all the associated RNAs had their 5' end around the mapped TSS[25,26] as confirmed by TIFseq[24] (Supplemental Fig. 8). Furthermore, after removal of glucose, the levels of transcripts derived from the 5'UTR did not alter over time (also when Nab3 had been depleted; Fig. 6e), whereas those reflective of *ENO1* transcription increased more than 2-fold (Fig. 6f). Overall, our data suggest that Pol II still finds the TSS but that under repressive conditions this happens slowly.

Formation of productive transcription initiation complexes at the *ENO1* TSS appear impeded on glucose. This is corroborated by mapping of pre-initiation complexes (PICs) of general transcription factors and Pol II[27], which – compared to *ENO2* – are not very abundant for *ENO1* around the respective TATA boxes and also locate around the TSS of *CUT166* in the URS (Supplementary Fig. 8c). Their low abundance is reflected in the low levels of transcription on glucose which after depletion of Nab3 only enhance slightly (Fig. 6e-f), in line with reduced NNS activity on the 5'UTR RNAs and the 5' end of the *ENO1* transcript. Protein factors involved with transcription initiation shape the DNA[28]. When repressive factors like Sgc1, that bind the URS just upstream of the TATA-box, would counteract this, formation of a productive transcription initiation complex would be delayed and thereby the association of Pol II with the TSS. When repression of *ENO1* is lifted, although transcription of *CUT166* still occurs, formation of an active transcription initiation complex at the TSS of *ENO1* is promoted. This could be realized by the release of repressive factors such as Sgc1. The level of induced transcription increases so rapidly that NNS-guided degradation – although increasing as well according to enhanced Nab3-crosslinking – does not have an overall impact on steady state levels. Possibly, NNS-mediated termination of transcripts will

happen on Pol II complexes that are slowed down or do not elongate properly, so that ongoing *ENO1* transcription will not be affected.

Supplementary Methods

Yeast strains and media

Saccharomyces cerevisiae strain BY4741 (MATa; *his3Δ1; leu2Δ0; met15Δ0; ura3Δ0*) was used as the main parental strain[29]. The HTP (HIS6-TEV-2xProtA) carboxyl-tagged strains (Calmodulin binding peptide-TEV-2xProtA) were generated by PCR as described[30,31]. Strains used in this study are listed in Supplementary Table 1. For the anchor away experiments we fused Nab3 to the FRB domain and integrated a HTP-tag at the 3' end of the Rpo21 gene in the HHY168 strain[1]. Rapamycin was added to the media at a final concentration of 1 µg/ml. For the glucose deprivation experiments, strains were grown in synthetic medium lacking tryptophan (Formedium) in the presence (SD-TRP) or absence of glucose (S-TRP). For the PAR-CLIP experiments, cells were grown in synthetic glucose-containing medium lacking tryptophan and uracil (SD-URA-TRP). Strains carrying changes in cross-linked Nab3-motifs were generated by site-directed mutagenesis in two steps using the Delitto-Perfetto system[32]; first a small deletion was generated covering the *YBR085C-A* Nab3 motifs by insertion of the cassette from plasmid pGSHU, which was then replaced by a gBlock (Integrated DNA Technologies) containing the mutations. Resultant strains were checked by sequencing and for normal growth on glycerol.

Western blot analyses

Western blot analysis was performed using the polyclonal rabbit anti-TAP antibody from Thermo Fisher (CAB1001), which recognizes the spacer between the TEV cleavage site and the six histidines. Blots were incubated with the antibody (1:5000 dilution) in blocking buffer (5% nonfat milk powder, 0.1% Tween-20 and phosphate buffer saline (PBS)) for one hour at room temperature (diluted 1:5000 in blocking buffer. Following two five minute washes with PBS-0.1% Tween, the blots were then incubated with goat anti-rabbit antibodies (Thermo Fisher (31466) 1:5000 in blocking buffer) for one hour at room temperature. Proteins were visualized using the Pearce enhanced chemiluminescence solutions as described by the manufacturer's procedures.

Quantitative RT-PCR

Cells were grown in SD-TRP to an OD_{600} of 0.4, harvested by filtration and then shifted to S-TRP. Cells were harvested before the shift (0) and 20, 40 minutes after the shift. RNA was extracted using the Guanidium thiocyanide method[33] or the masterpure yeast RNA purification kit (Epicentre) and quantitative RT-PCR was carried out using the Agilent Brilliant III SYBR master mix, using oligonucleotides listed in Supplementary Table 3. In an end-volume of 10 µl, 12 µg total RNA was treated with 2 units Turbo DNAseI (Ambion, ThermoFisher) in the presence of 4 units RNAsin (Promega) at 37ºC for 1 hour, followed by 15 min. at 65ºC to inactivate the enzyme. After addition of 2.5 µl 2.5 µM reverse PCR primers the nucleic acids are denatured at 85ºC for 3 min and then cooled on ice for 5 min. Of the annealed RNA/oligo mixture, 5 µl is added to a tube

containing 5 µl 2*RT-mix (100 u Superscript III reverse transcriptase (Invitrogen), 2 µl 5xFirstStrandBuffer, 0.5 µl 0.1 M DTT, 1.5 µl 5 mM dNTP mix, 1 u RNAsin, H_2O to 5 µl) and another 5 µl to a 2*NoRT-mix (as 2*RT mix but with H_2O replacing the enzyme) and incubated for 90 min at 55ºC. The enzyme is inactivated for 20 min at 65ºC and RNA/cDNA hybrids are resolved by digestion with 5 units RNAse H for 30 min at 37ºC. The mixture is diluted by adding 200 µl H_2O and 3 µl is added to 7 µl QPCR mix (3 µl water with 1µM of each primer, 4 µl 2*Brilliant III SYBR master) and amplified on a LightCycler 96 or 480 (Roche): 1 cycle 95ºC; 40 cycles (plate reading at end of each cycle) 95ºC 5s; 60ºC 10s; 72ºC 15s. A melt curve was generated by ramping to 95ºC (0.11; 5/°C) with continuous reading. QPCR reactions on each plate were done in triplicate.

Northern blot analysis

Total RNA was resolved on a 1.25% Agarose Bis-Tris (pH 7) gel and transferred to nitrocellulose. Northern blotting was performed using UltraHyb hybridization buffer according to the manufacturer's procedures (Ambion). The snR13 oligo sequence used for hybridization is provided in Supplementary Table 3.

Supplementary Tables

Strain	Genotype	Reference
BY4741	MATa; *his3Δ1; leu2Δ0; met15Δ0; ura3Δ0*	[29]
HHY168	MATα; *tor1-1; fpr1::NAT RPL13A-2×FKBP12::TRP*	[1]
HHY110	MATα; *tor1-1; fpr1::NAT PMA1-2×FKBP12::TRP*	[1]
YSG882	As BY4741 but with *nab3::HTP::K.l.URA3*	This study
YSG1013	As BY4741 but with *rpo21::HTP::K.l.URA3*	This study
YSG1010	As HHY110 but with *rpo21::HTP::K.l.URA3*	This study
YSG1042	As HHY168 but with *nab3::FRB::KAN and rpo21::HTP::K.l.URA3*	This study
YSG1051	As BY4741 but with allele *YBR085C*-B1547*	This study

Supplementary Table 1. *Saccharomyces cerevisiae* strains used in this study

5' adapters	Sequence (5'-3')
L5Aa	invddT-ACACrGrArCrGrCrUrCrUrUrCrCrGrArUrCrUrNrNrNrUrArArGrCrN
L5Ab	invddT-ACACrGrArCrGrCrUrCrUrUrCrCrGrArUrCrUrNrNrNrArUrUrArGrCrN
L5Ac	invddT-ACACrGrArCrGrCrUrCrUrUrCrCrGrArUrCrUrNrNrNrGrCrGrCrArGrCrN
L5Ad	invddT-ACACrGrArCrGrCrUrCrUrUrCrCrGrArUrCrUrNrNrNrCrGrCrUrUrArGrCrN
L5Ba	invddT-ACACrGrArCrGrCrUrCrUrUrCrCrGrArUrCrUrNrNrNrArGrArGrCrN
L5Bb	invddT-ACACrGrArCrGrCrUrCrUrUrCrCrGrArUrCrUrNrNrNrGrUrGrArGrCrN
L5Bc	invddT-ACACrGrArCrGrCrUrCrUrUrCrCrGrArUrCrUrNrNrNrCrArCrUrArGrCrN
L5Bd	invddT-ACACrGrArCrGrCrUrCrUrUrCrCrGrArUrCrUrNrNrNrUrCrUrCrUrArGrCrN
L5Ca	invddT-ACACrGrArCrGrCrUrCrUrUrCrCrGrArUrCrUrNrNrNrCrUrArGrCrN
L5Cb	invddT-ACACrGrArCrGrCrUrCrUrUrCrCrGrArUrCrUrNrNrNrUrGrGrArGrCrN
L5Cc	invddT-ACACrGrArCrGrCrUrCrUrUrCrCrGrArUrCrUrNrNrNrArCrUrCrArGrCrN
L5Cd	invddT-ACACrGrArCrGrCrUrCrUrUrCrCrGrArUrCrUrNrNrNrGrArCrUrUrArGrCrN
L5Da	invddT-ACACrGrArCrGrCrUrCrUrUrCrCrGrArUrCrUrNrNrNrCrGrUrGrArUrN
L5Db	invddT-ACACrGrArCrGrCrUrCrUrUrCrCrGrArUrCrUrNrNrNrGrCrArCrUrArN
L5Dc	invddT-ACACrGrArCrGrCrUrCrUrUrCrCrGrArUrCrUrNrNrNrUrArGrUrGrCrN
L5Dd	invddT-ACACrGrArCrGrCrUrCrUrUrCrCrGrArUrCrUrNrNrNrArUrCrArCrGrN
L5Ea	invddT-ACACrGrArCrGrCrUrCrUrUrCrCrGrArUrCrUrNrNrNrCrArCrUrGrUrN
L5Eb	invddT-ACACrGrArCrGrCrUrCrUrUrCrCrGrArUrCrUrNrNrNrGrUrGrArCrArN
L5Ec	invddT-ACACrGrArCrGrCrUrCrUrUrCrCrGrArUrCrUrNrNrNrUrGrUrCrArCrN
L5Ed	invddT-ACACrGrArCrGrCrUrCrUrUrCrCrGrArUrCrUrNrNrNrArCrArGrUrGrN

3' adapter	Sequence (5'-3')
App-PE	App-NAGATCGGAAGAGCACACG

RT primer	Sequence (5'-3')
PE-RT	CAGACGTGTGCTCTTCCGATCT

PCR primers	Sequence (5'-3')
**Forward primer:**	
P5	AATGATACGGCGACCACCGAGATCTACACTCTTTCCCTACACGACGCTCTTCCGATCT
**Reverse indexing primers:**	
BC1	CAAGCAGAAGACGGCATACGAGATCGTGATGTGACTGGAGTTCAGACGTGTGCTCTTCCGATCT
BC2	CAAGCAGAAGACGGCATACGAGATACATCGGTGACTGGAGTTCAGACGTGTGCTCTTCCGATCT
BC3	CAAGCAGAAGACGGCATACGAGATGCCTAAGTGACTGGAGTTCAGACGTGTGCTCTTCCGATCT
BC4	CAAGCAGAAGACGGCATACGAGATTGGTCAGTGACTGGAGTTCAGACGTGTGCTCTTCCGATCT
BC5	CAAGCAGAAGACGGCATACGAGATCACTGTGTGACTGGAGTTCAGACGTGTGCTCTTCCGATCT
BC6	CAAGCAGAAGACGGCATACGAGATATTGGCGTGACTGGAGTTCAGACGTGTGCTCTTCCGATCT
BC7	CAAGCAGAAGACGGCATACGAGATCAGATCGTGACTGGAGTTCAGACGTGTGCTCTTCCGATCT
BC8	CAAGCAGAAGACGGCATACGAGATTAGCTTGTGACTGGAGTTCAGACGTGTGCTCTTCCGATCT
BC9	CAAGCAGAAGACGGCATACGAGATGATCAGGTGACTGGAGTTCAGACGTGTGCTCTTCCGATCT
BC10	CAAGCAGAAGACGGCATACGAGATATCACGGTGACTGGAGTTCAGACGTGTGCTCTTCCGATCT

Supplementary Table 2 - Oligonucleotides for χCRAC library preparation. Lowercase 'r' indicates RNA nucleotides, 'rN' indicate random RNA nucleotides and blue sequences are barcodes.

qPCR primer	Sequence (5'-3')
ACT1-exon2-F	TGTTTTGGATTCCGGTGATGG
ACT1-exon2-R	ACCGGCCAAATCGATTCTCA
PIC2-qFwd	CCGCTGAATTCCTCGCTGAT
PIC2-qRev	TTGCAAAAGGGCGGCATAGT
NRD1-qFwd	TCCAGCCAAACAGCAAGCAG
NRD1-qRev	TGTATGGGCTTGGGGAGGTG
MAL33-qFwd	CGGACCCTCGACTTTCTTTGG
MAL33-qRev	GACTCCGTAGCCAAAGCATCAAA
IMD3-qFwd	TCCAATGGACACCGTGACAGA
IMD3-qRev	GTCAGCTTGGTCCTCTGGGGTA
ENO1-5UTR-Fwd	CTGTGGCCTTTTCTGGCACA
ENO1-5UTR-Rev	AGGGATTACAAGAGAGATGTTACAAGAAAGAA
ENO1-Fwd	TGCCGCTGCTGAAAAGAATG
ENO1-Rev	CGTGGGAACCACCGTTCAA
YBR085C-A-Fwd	CGACAGTCAGTGGGTACCAGGA
YBR085C-A-Rev	CGATGACTTCGCCGTCCTTT
TY1-2Fwd	ACGCTACACACGTCATCGACATC
TY1-2Rev	TGCTGTTTCTCGATCCCTGTTG
TY1-3Fwd	CGCCTCTGAGCACTCCATCA
TY1-3Rev	GGTGAGGTTAACATTGGTGGTGGT
TY1-4Fwd	CACCTGGGCCACAATCACAG
TY1-4Rev	AGAGTCCGCTGAGGATGAATCAGT
TY2-1Fwd	CTCGCACATCTCCAAACACGA
TY2-1Rev	TGTGAGCTTTTGCTGCTCTTGG
TY2-3Fwd	AAACGTCACCTGCGTATTATCAACC
TY2-3Rev	TCGATTGGATCAGGTGAGGAAGT
TY2-4Fwd	CTGAGTACTTCCTCACCTGATCCAATC
TY2-4Rev	TTCTGATGTTAAAGTGTGTGGTGGTAAGA

Northern primer	Sequence (5'-3')
snr13_Northern	GCCAAACAGCAACTCGAGCCAAATGCACTCATATTCATC

Supplementary Table 3 - Oligonucleotides used for qRT-PCR and Northern blot analysis

Glucose*	No Glucose (4 minutes)	No Glucose (18 minutes)
CUP1-1	AHA1	ACA1
FLX1 (snR68)	ARG82	BAP3
GRE3	ATP1	GUT2
HEM4 (snR5)	DIP5	MAL31
HIS1	ECL1	MAL33
ICT1	ERO1	NRD1
IMD2	GNP1	PIC2
IMD3	GRE3	RHO5
NRD1	GUT2	UIP4
SEN2 (snR79)	HIS4	YLR171W
TRS31 (snR13)[‡]	NRD1	YLR410W-B
URA8	RPN4	YOR059C
YCL007C (snR43)	TRS31	
	URA8	
	WTM1	
	YAR010C	
	YTM1	

*) Genes in italics might be identified due to transcripts originating from a preceding snoRNA gene (in brackets) that is not properly terminated in absence of Nab3.
[‡]) See also Supplementary Fig. 5b (Northern blot probed for snR13) and Supplementary Fig. 6c.

Supplementary Table 4 - List of genes controlled by Nab3 attenuation

Supplementary Figure Legends

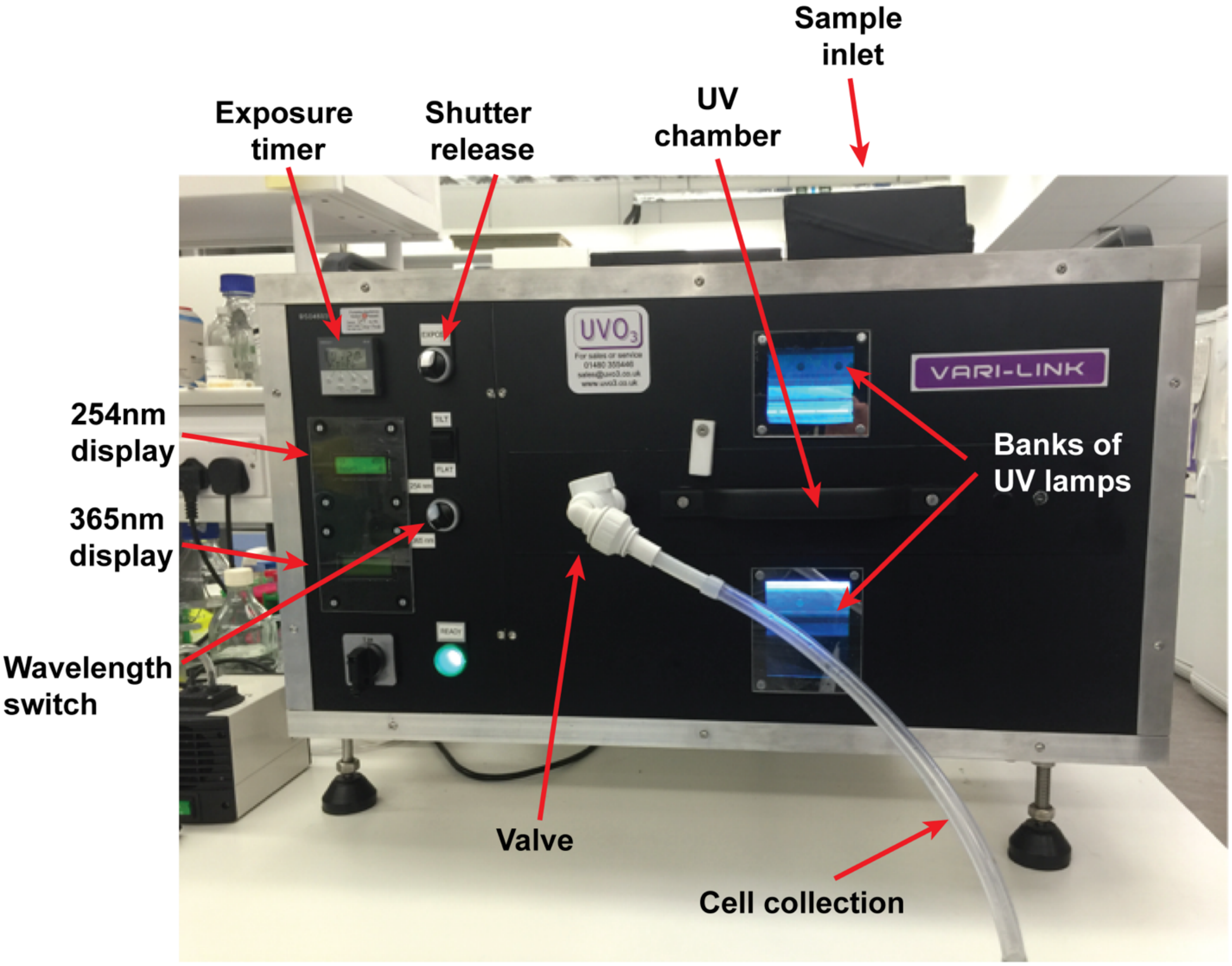

Supplementary Figure 1. The Vari-X-linker

Picture of the latest Vari-X-linker prototype. Cells are poured into the machine on the top of the unit where the sample enters the UV-irradiation chamber. Sample is UV irradiated from both sides and irradiation length can be controlled via a shutter system. Indicated are some of the features of the machine. The machine can be loaded with 254nm and 365 nm lamps, which can easily be swapped. The cells can be extracted by attaching a pump to the tube connected to the valve. The cells are loaded into the UV chamber on the top of the unit. Separate UV sensors were built in for 254nm and 365mm lamps, which shows at what percentage of the maximum output the lamps are. The machine also has a tilting mechanism to allow the bag in the UV chamber to empty completely.

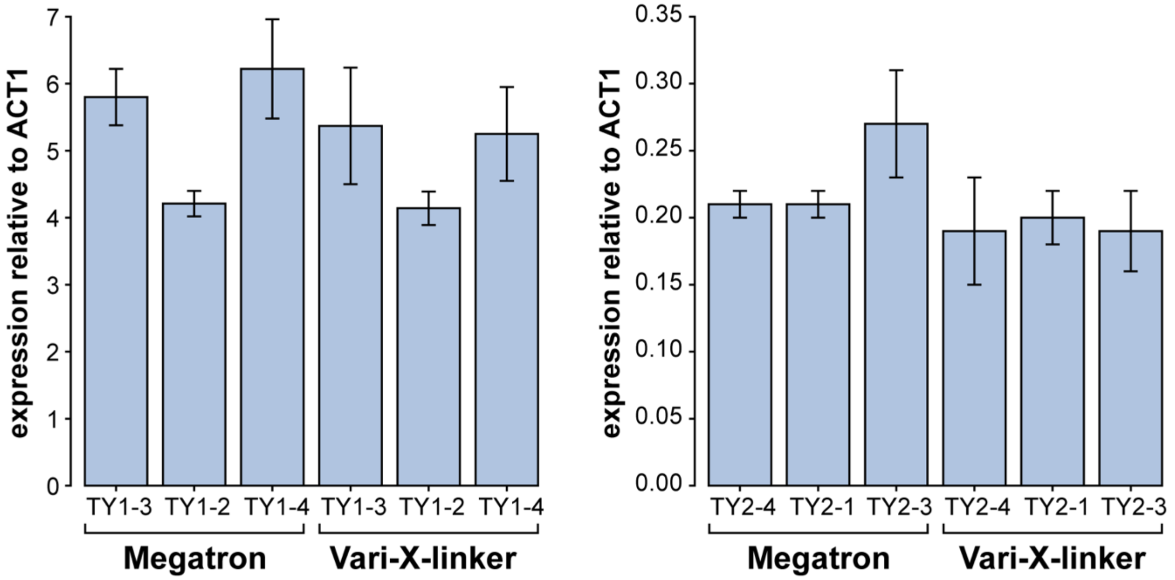

Supplementary Figure 2. Ty1 and Ty2 transcript levels do not significantly change during UV irradiation.

Cells were cross-linked in the Megatron (100 seconds) and the Vari-X-linker (12 seconds) and qRT-PCR was performed with three different primer sets on total RNA to measure levels three variants of Ty1 and Ty2 retrotransposons. Transcript levels were normalized to those of the *ACT1* gene (y-axis). Error bars indicate s.d. from four experimental replicates.

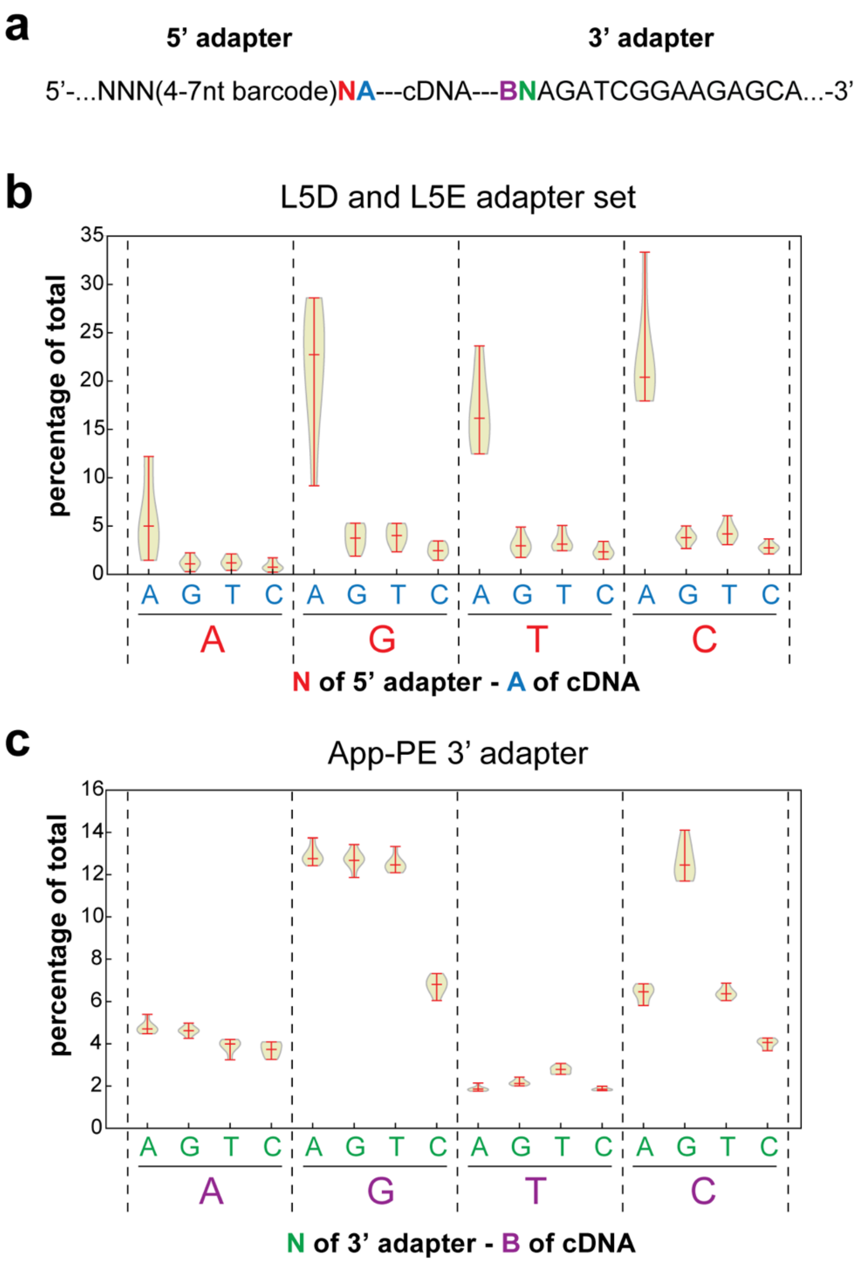

Supplementary Figure 3. T4 RNA ligase has preference for specific nucleotide donor-acceptor nucleotide combinations.

(**a**) Adapter strategy. Both 5' and 3' adapters contain a random nucleotide at the termini that ligate to the cDNA (red colored and green colored "N"). The blue "A" in the sequence indicates the first nucleotide of the cDNA that ligates to the 5' adapter. The purple "B" indicates the last nucleotide that ligates to the 3' adapter. All adapter sequences are provided in Supplementary Table 2.

(**b**-**c**) T4 RNA ligase has a preference for specific nucleotide combinations. The violin plots show the distribution of donor-acceptor nucleotides found in a Pol II χCRAC dataset using the L5D and L5E series of 5' adapters. The data show that T4 RNA ligase has a clear preference for specific donor-acceptor nucleotide combination, with an A being the preferred nucleotide at the 5' end of cDNAs. The variability in some of the data points indicates that the surrounding nucleotides can influence the ligation efficiency. However, there was not a specific 5' adapter barcode sequence that showed significantly better or worse ligation efficiencies. The violin plot in (**c**) shows that the truncated T4 RNA ligase (NEB) has a strong preference for a G or a C at the 5' end of the 3' adapter sequence, although GC and CC combinations are less enriched.

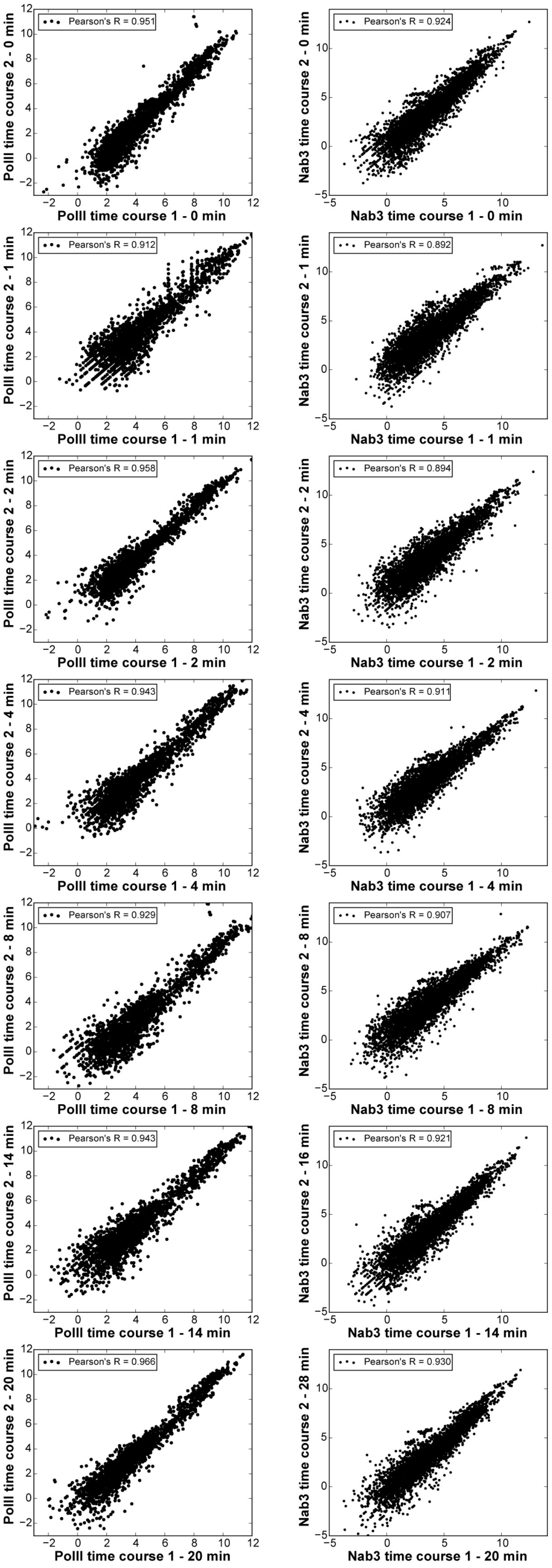
Pearson's R = 0.951
PolII time course 2 - 0 min
PolII time course 1 - 0 min
Pearson's R = 0.924
Nab3 time course 2 - 0 min
Nab3 time course 1 - 0 min
Pearson's R = 0.912
PolII time course 2 - 1 min
PolII time course 1 - 1 min
Pearson's R = 0.892
Nab3 time course 2 - 1 min
Nab3 time course 1 - 1 min
Pearson's R = 0.958
PolII time course 2 - 2 min
PolII time course 1 - 2 min
Pearson's R = 0.894
Nab3 time course 2 - 2 min
Nab3 time course 1 - 2 min
Pearson's R = 0.943
PolII time course 2 - 4 min
PolII time course 1 - 4 min
Pearson's R = 0.911
Nab3 time course 2 - 4 min
Nab3 time course 1 - 4 min
Pearson's R = 0.929
PolII time course 2 - 8 min
PolII time course 1 - 8 min
Pearson's R = 0.907
Nab3 time course 2 - 8 min
Nab3 time course 1 - 8 min
Pearson's R = 0.943
PolII time course 2 - 14 min
PolII time course 1 - 14 min
Pearson's R = 0.921
Nab3 time course 2 - 16 min
Nab3 time course 1 - 14 min
Pearson's R = 0.966
PolII time course 2 - 20 min
PolII time course 1 - 20 min
Pearson's R = 0.930
Nab3 time course 2 - 28 min
Nab3 time course 1 - 20 min

Supplementary Figure 4. χCRAC generates highly reproducible data.

The scatter plots show the pairwise comparison of Pol II (left) and Nab3 (right) biological replicates for each time point (0-20 min). All datasets were log2 transformed before Pearson correlations were calculated. Pearson's R correlations between the time-points are shown in the top left corner.

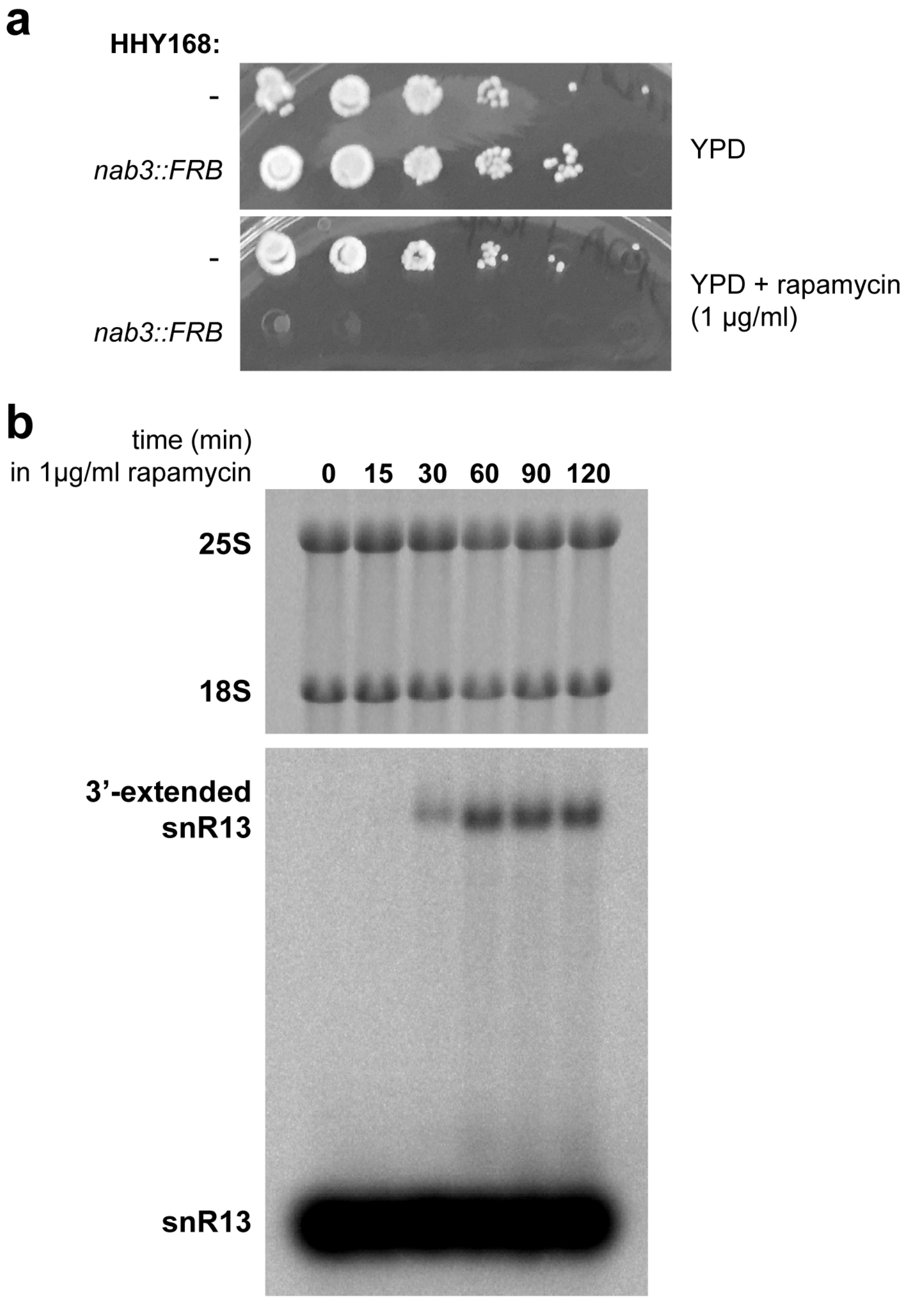

Supplementary Figure 5. The Nab3-FRB strain can be effectively used to deplete Nab3 from the nucleus.

(**a**) Cells expressing Nab3-FRB die on plates with rapamycin. Shown is a serial dilution assay where growth of the parental strain (HHY168[3]) is compared to the strain expressing Nab3-FRB. Cells were spotted on YPD and YPD supplemented with rapamycin (1μg/μl). The results show that rapamycin treatment of the *nab3::frb* strain is lethal.

(**b**) Northern blot analysis of 3' extended snR13 species during a rapamycin time-course. The HHY168 *nab3::FRB* strain was grown in YPD to exponential phase. Cells were harvested shortly at exponential phase (t=0). Subsequently, rapamycin was added to a final concentration of 1µg/ml and cells were harvested 15, 30, 60, 90 and 120 minutes after adding the drug. Total RNA was extracted and resolved on a 1.25% agarose gel. Ribosomal RNAs (25S and 18S) were detected by SyBr safe staining. After transferring the RNA to a nitrocellulose membrane, the blot was probed with an anti-sense snR13 oligonucleotide (Supplementary Table 2). After about 60 minutes of rapamycin treatment the amount of 3' extended snR13 species reached its maximum level. Therefore, for subsequent depletion experiments a 60-minute rapamycin incubation was used.

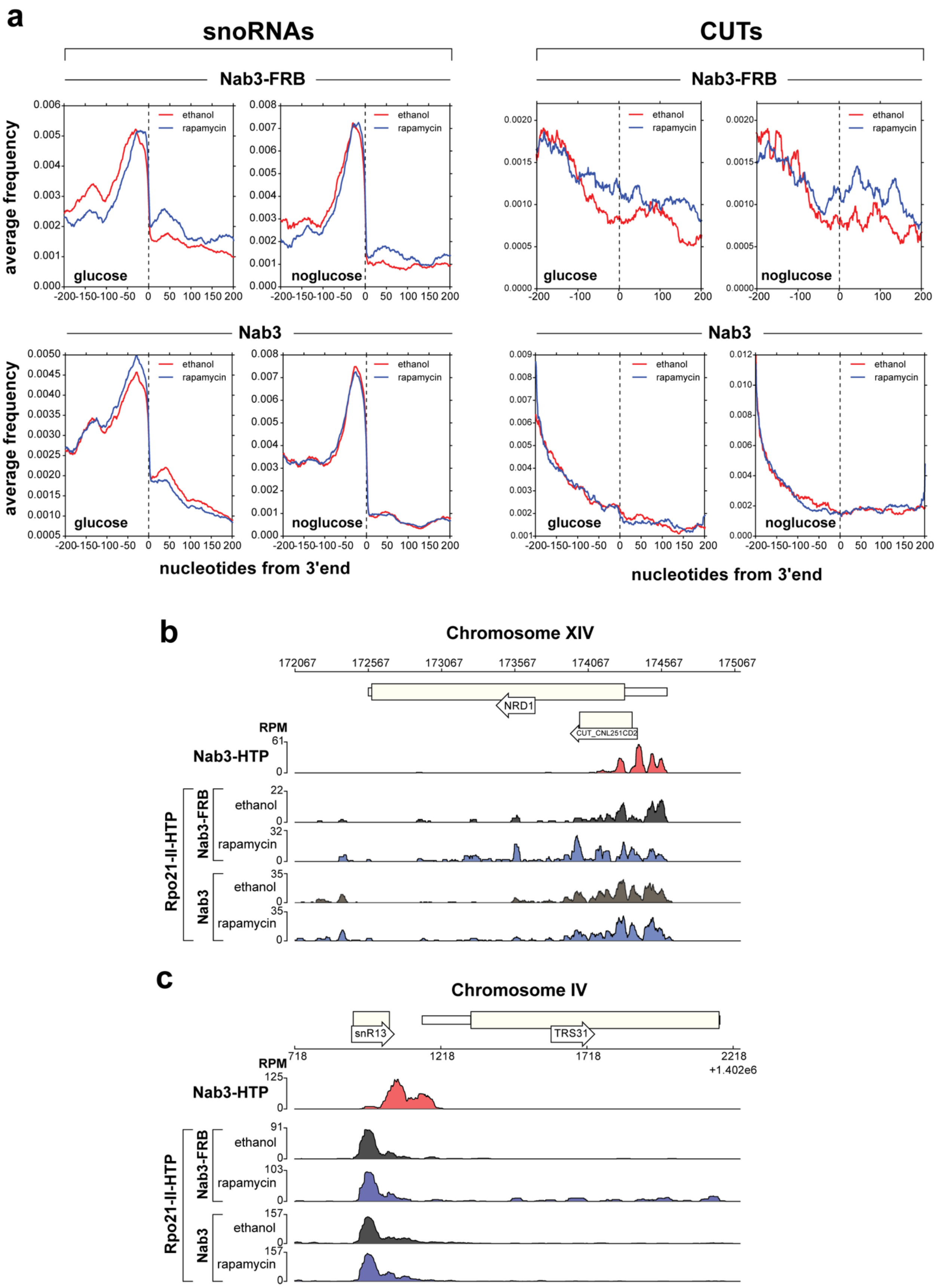
a
snoRNAs
CUTs
Nab3-FRB
Nab3
average frequency
ethanol
rapamycin
glucose
noglucose
nucleotides from 3'end
b
Chromosome XIV
172067
172567
173067
173567
174067
174567
175067
NRD1
CUT_CNL251CD2
RPM
Nab3-HTP
Rpo21-II-HTP
Nab3-FRB
Nab3
ethanol
rapamycin
c
Chromosome IV
snR13
TRS31
718
1218
1718
2218
+1.402e6

Supplementary Figure 6. Nuclear depletion of Nab3 results in the accumulation of 3' extended CUTs and snoRNAs.

(**a**) Distribution of reads that mapped to CUTs and snoRNAs around the 3' end of the features (x-axis). Cells expressing the FRB-tagged Nab3 and the parental strain (Nab3) were grown in glucose to exponential phase and rapidly shifted to medium lacking glucose. Cells were harvested before the shift (glucose panels) or 14 (Nab3) to 18 minutes (Nab3-FRB) after the shift (no glucose panels). Reads mapped to each features were divided over 400 bins (1nt per bin) and for each bin the fraction of total reads that mapped to each bin was calculated. These numbers were then averaged (y-axis) to generate the plots. The reads for the rapamycin treated cells are represented as a blue line, whereas the reads for the ethanol (control) experiment are represented as a red line. The results show an increase in read density downstream of the annotated 3' ends of CUTs and snoRNAs in rapamycin treated *nab3::frb* cells (but not the parental strain), indicative of defects in transcription termination.

(**b**-**c**) Nab3-dependent termination of known targets are detectable in Pol II χCRAC experiments. Shown are genome browser images of the Nrd1 and snR13 genes. On the y-axis the number of reads per million mapped reads (RPM) is plotted. The top track shows the Nab3 cross-linking data, the second and third tracks show the Pol II cross-linking data in the ethanol and rapamycin treated *nab3::frb rpo21::HTP* strain, respectively. The fourth and fifth track shows the results from the ethanol and rapamycin treated *rpo21::HTP* anchor-away strain.

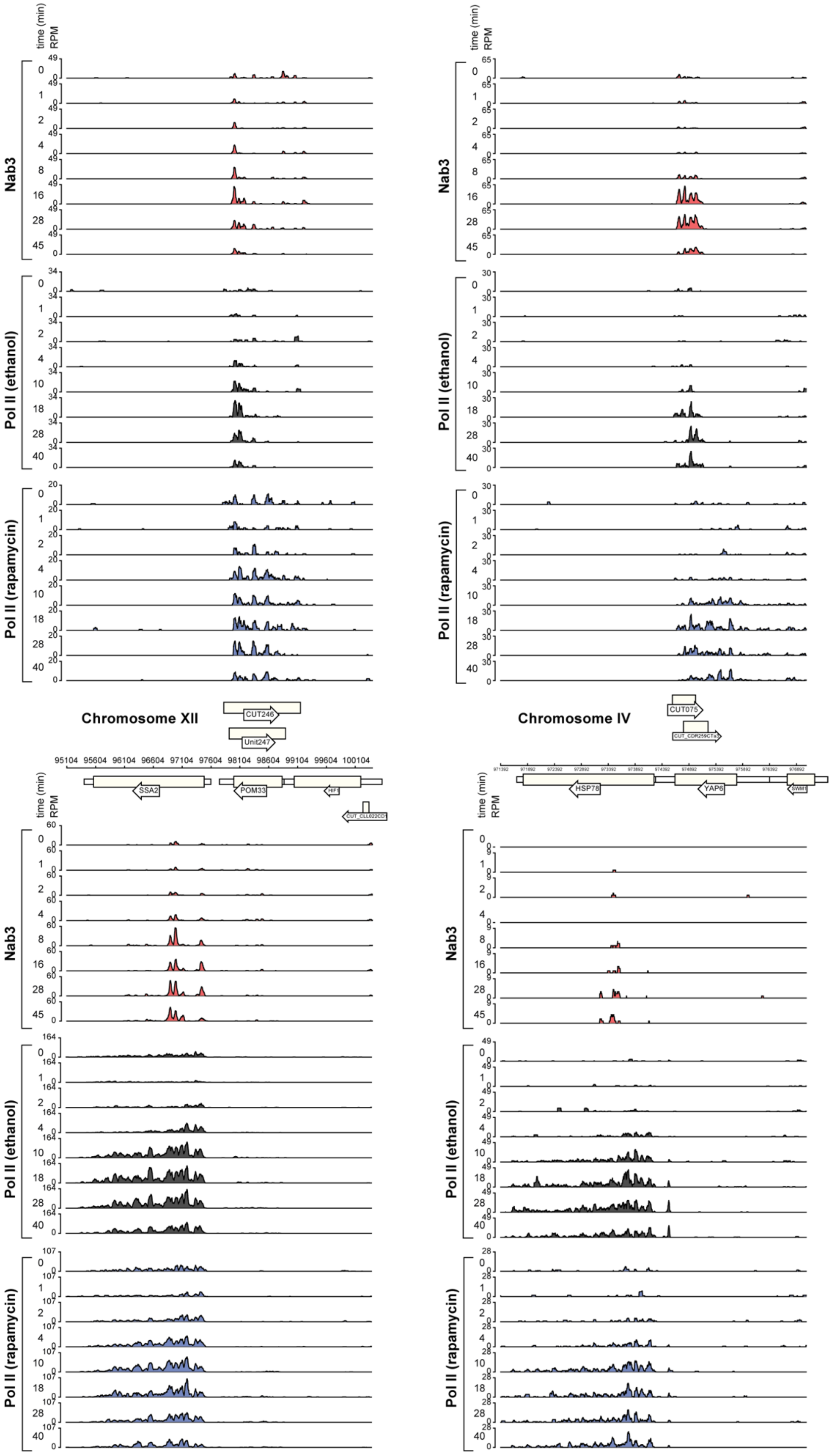
time (min)
RPM
Nab3
Pol II (ethanol)
Pol II (rapamycin)
Chromosome XII
CUT246
Unit247
95104 95604 96104 96604 97104 97604 98104 98604 99104 99604 100104
SSA2
POM33
HIF1
Chromosome IV
CUT075
HSP78
YAP6

Supplementary Figure 7. Nab3 terminates anti-sense divergent cuts

Genome browser images for the *SSA2* and *HSP78* region showing Pol II χCRAC data from the solvent (ethanol; black), rapamycin treated cells (blue) and data from a Nab3 χCRAC experiment (red) for both strands. On the y-axis of each track the reads per million (RPM) mapped reads is plotted. The time points at which cells were harvested (in minutes) is indicated left of each track.

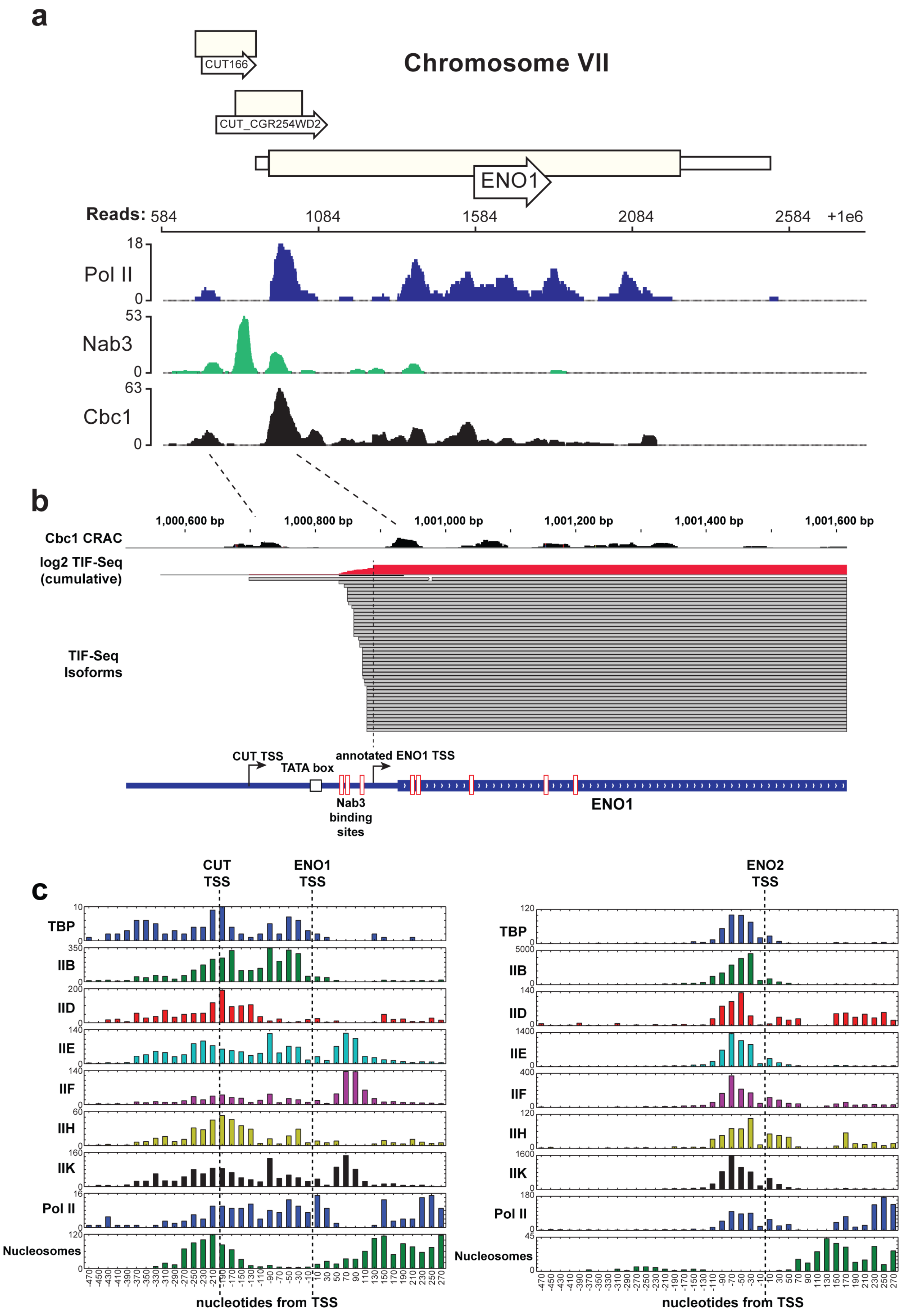
a
Chromosome VII
CUT166
CUT_CGR254WD2
ENO1
Reads: 584
1084
1584
2084
2584
+1e6
Pol II
Nab3
Cbc1
b
1,000,600 bp
1,000,800 bp
1,001,000 bp
1,001,200 bp
1,001,400 bp
1,001,600 bp
Cbc1 CRAC
log2 TIF-Seq (cumulative)
TIF-Seq Isoforms
CUT TSS
TATA box
annotated ENO1 TSS
Nab3 binding sites
ENO1
c
CUT TSS
ENO1 TSS
ENO2 TSS
TBP
IIB
IID
IIE
IIF
IIH
IIK
Pol II
Nucleosomes
nucleotides from TSS
nucleotides from TSS

Supplementary Figure 8. Cryptic transcription upstream of *ENO1*

(**a**) Genome browser snapshot of Pol II (blue), Nab3 (green) and cap-binding complex protein Cbc1 (black) CRAC data. The Cbc1 CRAC data was obtained from[23].

(**b**) Transcription can initiate upstream of the *ENO1* promoter. Genome browser snapshot of Cbc1 CRAC and transcription isoform sequencing data (TIFseq[24]. The first track shows the Cbc1 CRAC data. The second track shows the log2 of the cumulative density of all isoforms (red). The third track shows some of the transcript isoforms that mapped to *ENO1*. The position of the annotated *ENO1* TSS, Nab3 binding sites, *ENO1* TATA box and the TSS of the upstream CUT is shown in the fourth track.

(**c**) Chip-exo sequencing data of Pol II transcription factors[27] for *ENO1* and the orthologous *ENO2*. The y-axis shows the read density and the names of individual transcription factors (TFs). The x-axis shows the nucleotides away from the *ENO1* or *ENO2* transcription start site (TSS). Individual TSSs for *CUT166* and *ENO1* or *ENO2* are indicated with dashed lines. Note the at least 10-fold scale differences between *ENO2* and *ENO1* TF-data, reflecting the difference of transcription on glucose.

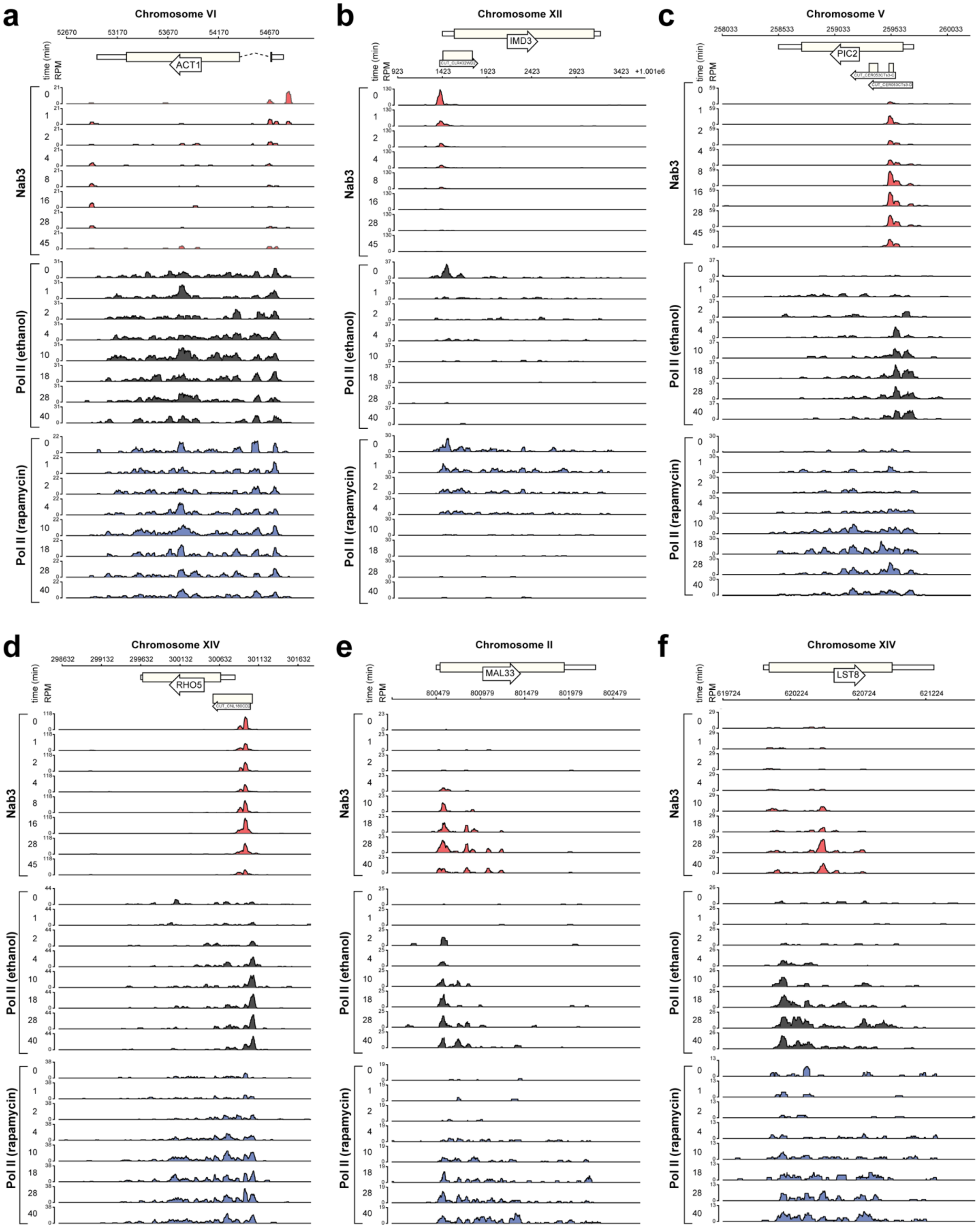
a
Chromosome VI
ACT1
b
Chromosome XII
IMD3
c
Chromosome V
PIC2
d
Chromosome XIV
RHO5
e
Chromosome II
MAL33
f
Chromosome XIV
LST8
time (min)
RPM
Nab3
Pol II (ethanol)
Pol II (rapamycin)

Supplementary Figure 9. Nab3 regulates the induction of stress-responsive protein-coding genes during glucose deprivation.

(**a**-**f**) Genome browser image showing the Pol II χCRAC from the solvent (ethanol; black), rapamycin treated cells (blue) and data from a Nab3 χCRAC experiment (red) for selected genes. On the y-axis of each track the reads per million (RPM) mapped reads are shown. The time points at which cells were harvested (in minutes) is indicated left of each track.

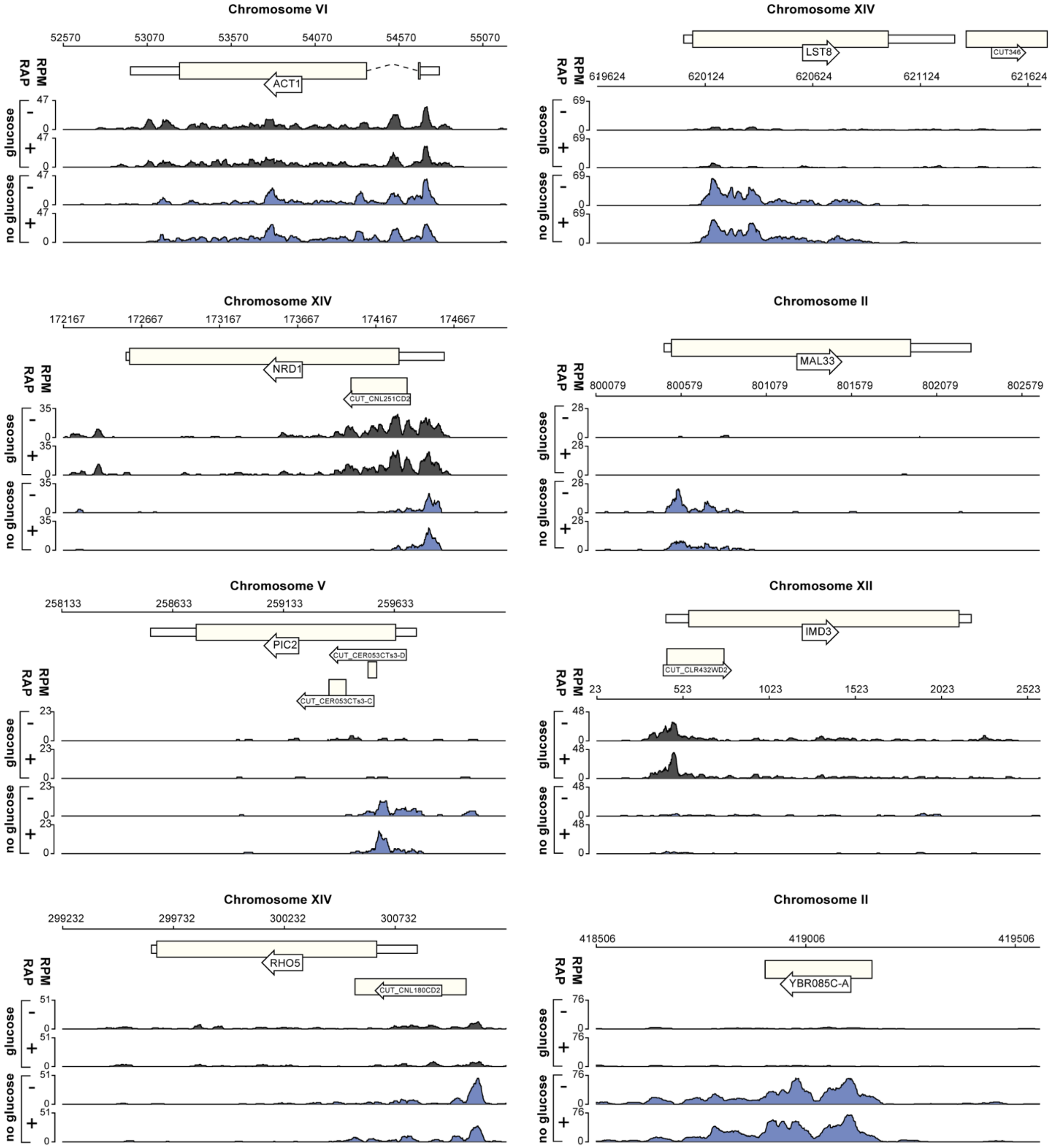
Chromosome VI
52570 53070 53570 54070 54570 55070
ACT1
RAP RPM
glucose
no glucose
Chromosome XIV
LST8
CUT346
619624 620124 620624 621124 621624
Chromosome XIV
172167 172667 173167 173667 174167 174667
NRD1
CUT_CNL251CD2
Chromosome II
MAL33
800079 800579 801079 801579 802079 802579
Chromosome V
258133 258633 259133 259633
PIC2
CUT_CER053CTs3-D
CUT_CER053CTs3-C
Chromosome XII
IMD3
CUT_CLR432WD2
23 523 1023 1523 2023 2523
Chromosome XIV
299232 299732 300232 300732
RHO5
CUT_CNL180CD2
Chromosome II
418506 419006 419506
YBR085C-A

Supplementary Figure 10. The changes in Pol II transcription profiles are not induced by the drug rapamycin itself.

Shown are genome browser images of CRAC data generated using the anchor-away strain expressing an HTP-tagged Rpo21. Cells were grown in glucose to exponential phase and incubated with ethanol (-) or rapamycin (RAP; +) for one hour. A fraction of the cells was harvested (glucose samples) and the rest was shifted to medium lacking glucose for 14 minutes. These data demonstrate that the drug rapamycin does not influence Pol II transcription of these genes.

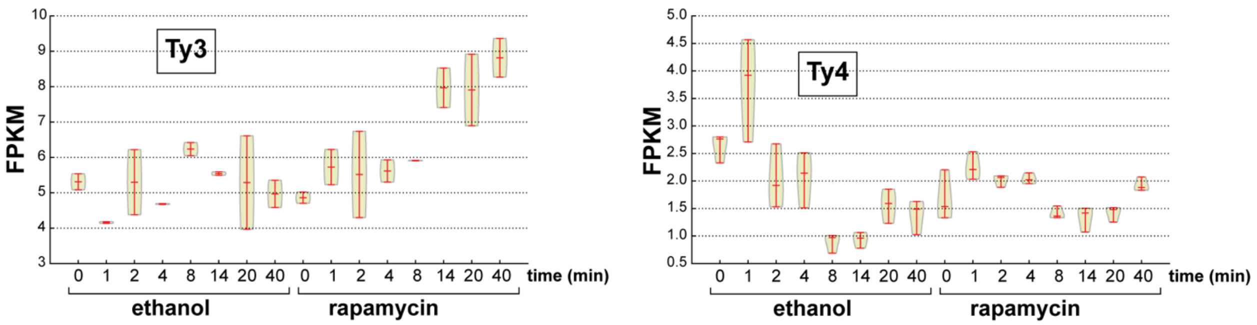

Supplementary Figure 11. Nab3 regulates the expression of Ty3 retrotransposons during glucose deprivation.

Violin plots showing the Ty3 and Ty4 pol II FPKM distribution from the *nab::frb* Rpo21-HTP χCRAC data generated in the presence of solvent (ethanol) or rapamycin. Shown are the averaged data from two independent experiments. Time (min) indicates the number of minutes in medium lacking glucose.

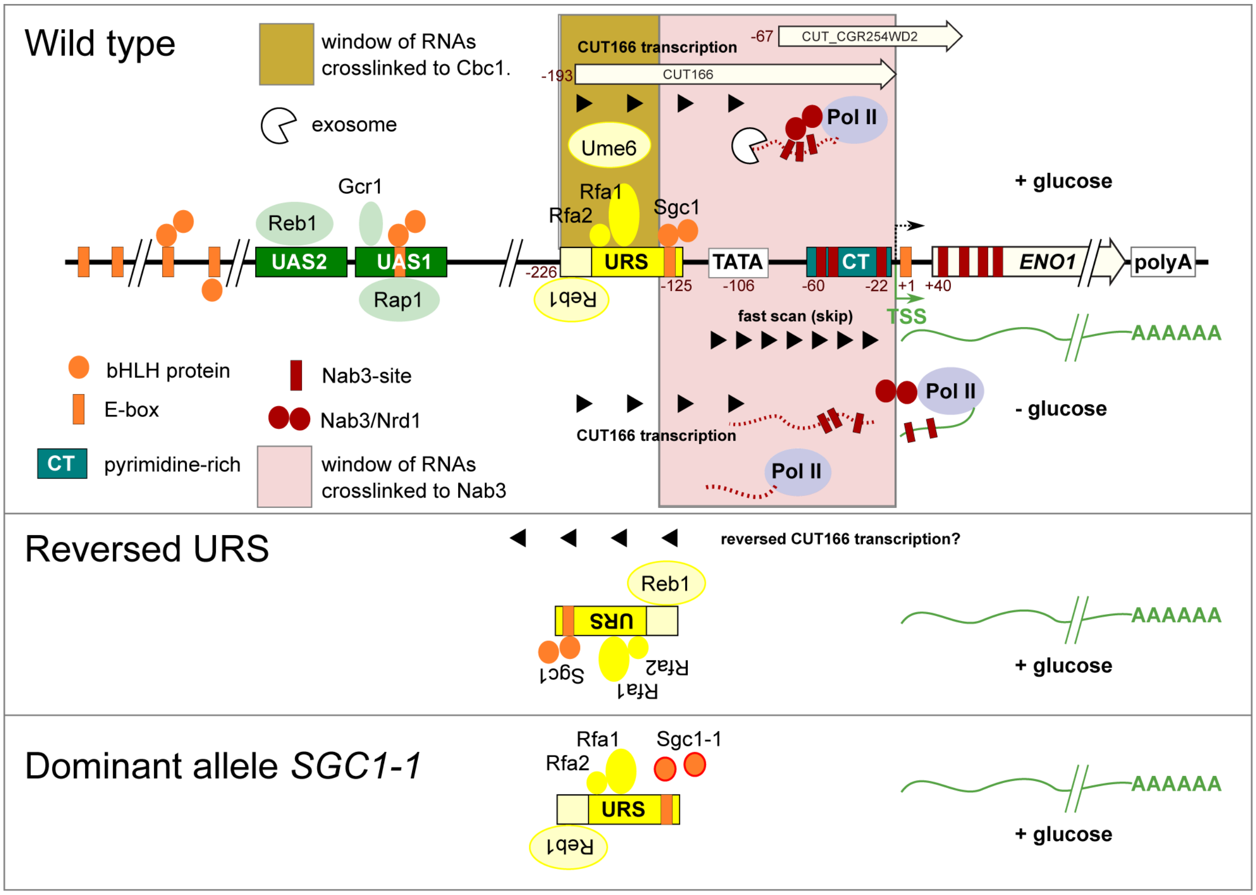

Supplementary Figure 12. Regulation of *ENO1* transcription is controlled by a repressive element.

Schematic overview of the *ENO1* promoter and control of transcription in the presence or absence of glucose. See Supplementary note 2 for details.

Figure 1a

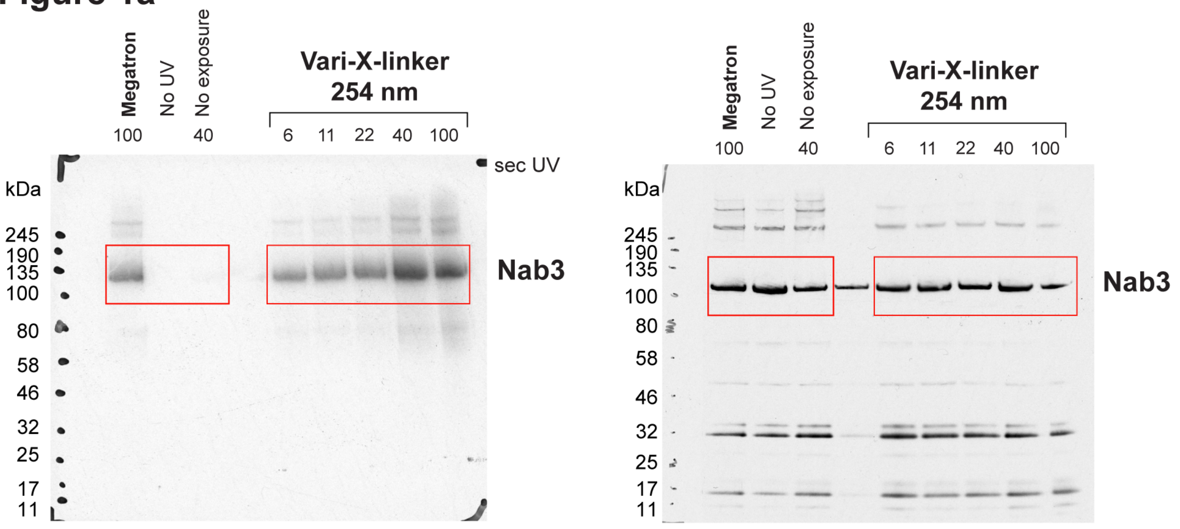

Figure 1b

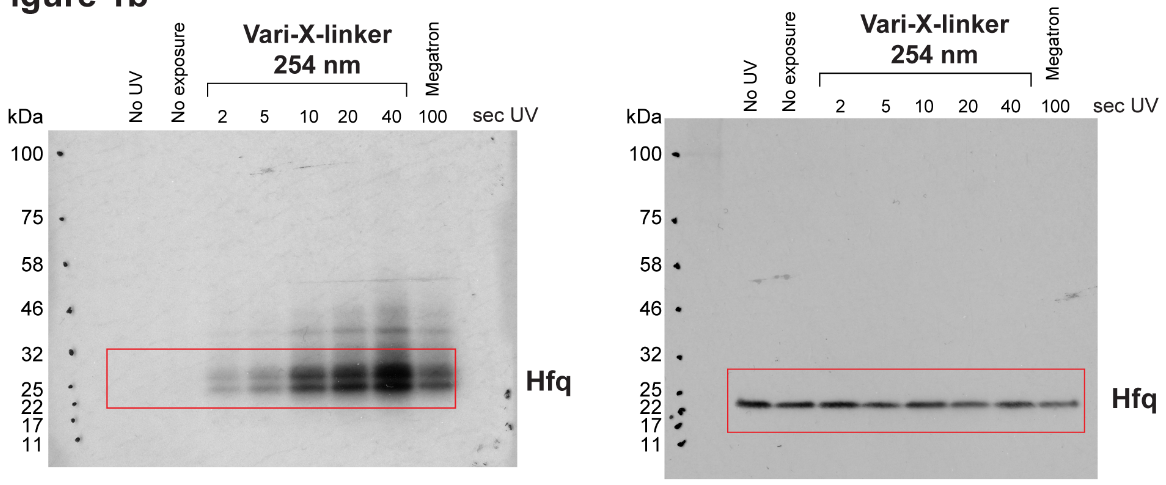

Figure 1c

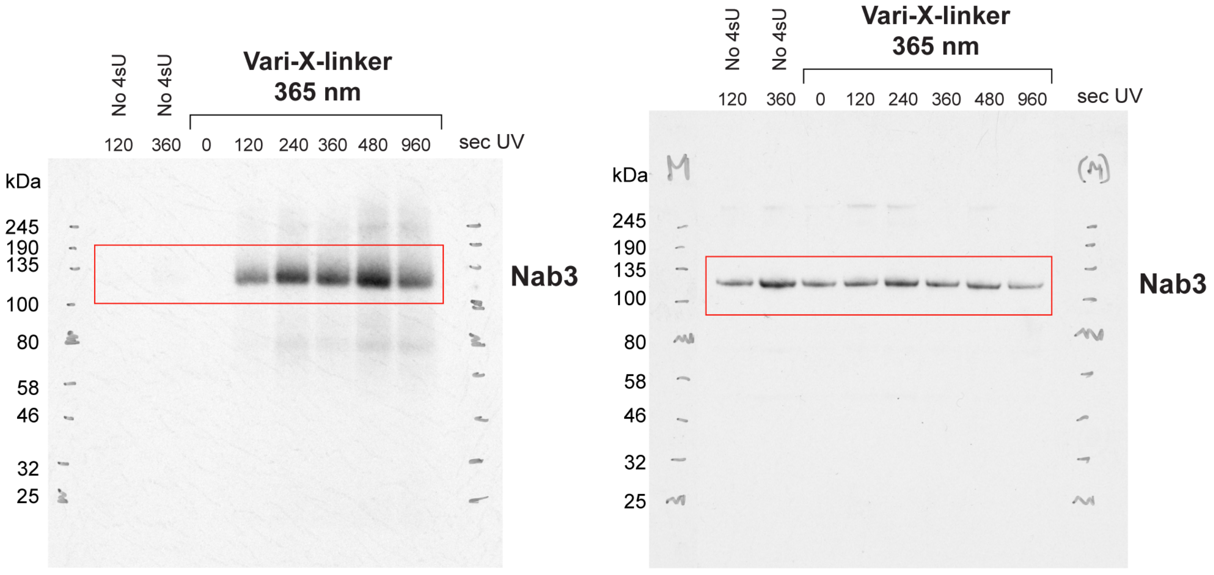

Supplementary Figure 14. Uncropped images.

Uncropped images of films or phosphoimager scans show in Figure 3b. The red boxes indicate the cropped regions.

Figure 3b

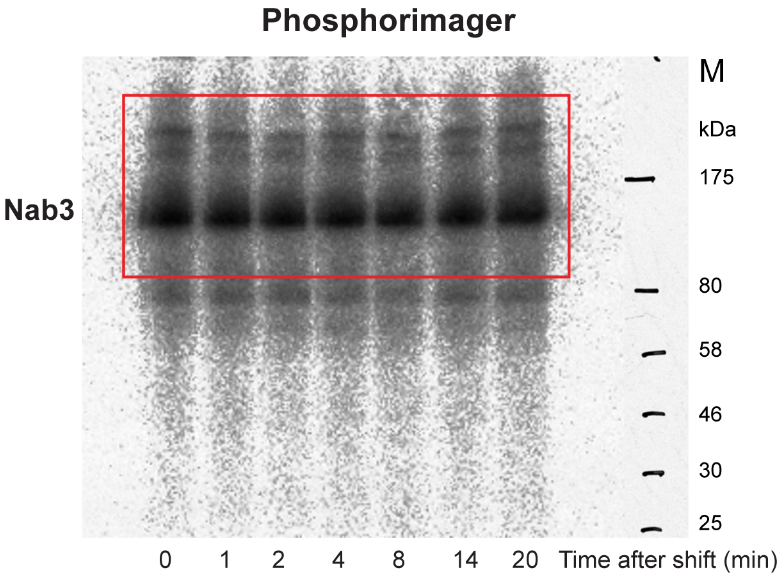

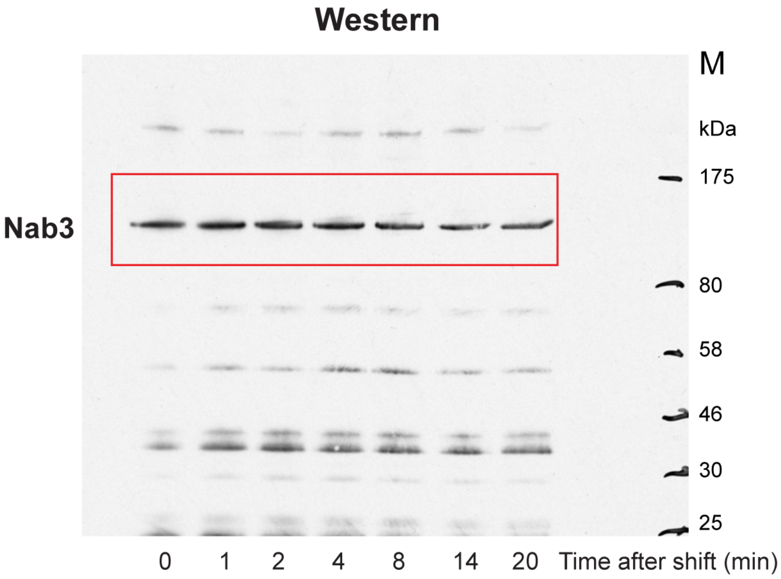

Supplementary Figure 14. Uncropped images.

Uncropped images of films or phosphoimager scans show in Figure 3b. The red boxes indicate the cropped regions.

Supplementary References

1. Haruki, H., Nishikawa, J. & Laemmli, U. K. The anchor-away technique: rapid, conditional establishment of yeast mutant phenotypes. *Mol. Cell* **31,** 925–932 (2008).
2. Steinmetz, E. J., Conrad, N. K., Brow, D. A. & Corden, J. L. RNA-binding protein Nrd1 directs poly(A)-independent 3'-end formation of RNA polymerase II transcripts. *Nature* **413,** 327–331 (2001).
3. Arigo, J. T., Carroll, K. L., Ames, J. M. & Corden, J. L. Regulation of yeast NRD1 expression by premature transcription termination. *Mol. Cell* **21,** 641–651 (2006).
4. Brindle, P. K., Holland, J. P., Willett, C. E., Innis, M. A. & Holland, M. J. Multiple factors bind the upstream activation sites of the yeast enolase genes ENO1 and ENO2: ABFI protein, like repressor activator protein RAP1, binds cis-acting sequences which modulate repression or activation of transcription. *Mol. Cell. Biol.* **10,** 4872–4885 (1990).
5. Machida, M., Jigami, Y. & Tanaka, H. Purification and characterization of a nuclear factor which binds specifically to the upstream activation sequence of Saccharomyces cerevisiae enolase 1 gene. *FEBS J.* **184,** 305–311 (1989).
6. Machida, M., Uemura, H., Jigami, Y. & Tanaka, H. The protein factor which binds to the upstream activating sequence of Saccharomyces cerevisiae ENO1 gene. *Nucleic Acids Res.* **16,** 1407–1422 (1988).
7. Carmen, A. A., Brindle, P. K., Park, C. S. & Holland, M. J. Transcriptional regulation by an upstream repression sequence from the yeast enolase gene ENO1. *Yeast* **11,** 1031–1043 (1995).
8. Carmen, A. A. & Holland, M. J. The upstream repression sequence from the yeast enolase gene ENO1 is a complex regulatory element that binds multiple trans-acting factors including REB1. *J. Biol. Chem.* **269,** 9790–9797 (1994).
9. Reimand, J., Vaquerizas, J. M., Todd, A. E., Vilo, J. & Luscombe, N. M. Comprehensive reanalysis of transcription factor knockout expression data in Saccharomyces cerevisiae reveals many new targets. *Nucleic Acids Res.* **38,** 4768–4777 (2010).
10. Harbison, C. T. *et al.* Transcriptional regulatory code of a eukaryotic genome. *Nature* **431,** 99–104 (2004).
11. Luche, R. M., Smart, W. C. & Cooper, T. G. Purification of the heteromeric protein binding to the URS1 transcriptional repression site in Saccharomyces cerevisiae. *Proc. Natl. Acad. Sci. U.S.A.* **89,** 7412–7416 (1992).
12. Luchelli, L., Thomas, M. G. & Boccaccio, G. L. Synaptic control of mRNA translation by reversible assembly of XRN1 bodies. *J. Cell. Sci.* **128,** 1542–1554 (2015).
13. Park, H.-D., Luche, R. M. & Cooper, T. G. The yeast UME6 gene product is required for transcriptional repression mediated by the CAR1 URS1 repressor binding site. *Nucleic Acids Res.* **20,** 1909–1915 (1992).
14. Fazzio, T. G. *et al.* Widespread collaboration of Isw2 and Sin3-Rpd3 chromatin remodeling complexes in transcriptional repression. *Mol. Cell. Biol.* **21,** 6450–6460 (2001).

15. Yadon, A. N., Singh, B. N., Hampsey, M. & Tsukiyama, T. DNA looping facilitates targeting of a chromatin remodeling enzyme. *Mol. Cell* **50,** 93–103 (2013).
16. Yadon, A. N. *et al.* Chromatin remodeling around nucleosome-free regions leads to repression of noncoding RNA transcription. *Mol. Cell. Biol.* **30,** 5110–5122 (2010).
17. Sato, T. *et al.* The E-box DNA binding protein Sgc1p suppresses the gcr2 mutation, which is involved in transcriptional activation of glycolytic genes in Saccharomyces cerevisiae. *FEBS Lett.* **463,** 307–311 (1999).
18. Chen, M. & Lopes, J. M. Multiple basic helix-loop-helix proteins regulate expression of the ENO1 gene of Saccharomyces cerevisiae. *Eukaryotic Cell* **6,** 786–796 (2007).
19. Gordân, R. *et al.* Genomic regions flanking E-box binding sites influence DNA binding specificity of bHLH transcription factors through DNA shape. *Cell Rep* **3,** 1093–1104 (2013).
20. Nishi, K. *et al.* The GCR1 requirement for yeast glycolytic gene expression is suppressed by dominant mutations in the SGC1 gene, which encodes a novel basic-helix-loop-helix protein. *Mol. Cell. Biol.* **15,** 2646–2653 (1995).
21. Neil, H. *et al.* Widespread bidirectional promoters are the major source of cryptic transcripts in yeast. *Nature* **457,** 1038–1042 (2009).
22. Xu, Z. *et al.* Bidirectional promoters generate pervasive transcription in yeast. *Nature* **457,** 1033–1037 (2009).
23. Tuck, A. C. & Tollervey, D. A transcriptome-wide atlas of RNP composition reveals diverse classes of mRNAs and lncRNAs. *Cell* **154,** 996–1009 (2013).
24. Pelechano, V., Wei, W. & Steinmetz, L. M. Extensive transcriptional heterogeneity revealed by isoform profiling. *Nature* **497,** 127–131 (2013).
25. Jigami, Y. *et al.* Analysis of expression of yeast enolase 1 gene containing a longer pyrimidine-rich region located between the TATA box and transcription start site. *J. Biochem.* **99,** 1111–1125 (1986).
26. Nagalakshmi, U. *et al.* The transcriptional landscape of the yeast genome defined by RNA sequencing. *Science* **320,** 1344–1349 (2008).
27. Rhee, H. S. & Pugh, B. F. Genome-wide structure and organization of eukaryotic pre-initiation complexes. *Nature* **483,** 295–301 (2012).
28. Sainsbury, S., Bernecky, C. & Cramer, P. Structural basis of transcription initiation by RNA polymerase II. *Nat. Rev. Mol. Cell Biol.* **16,** 129–143 (2015).
29. Brachmann, C. B. *et al.* Designer deletion strains derived from Saccharomyces cerevisiae S288C: a useful set of strains and plasmids for PCR-mediated gene disruption and other applications. *Yeast* **14,** 115–132 (1998).
30. Longtine, M. S. *et al.* Additional modules for versatile and economical PCR-based gene deletion and modification in Saccharomyces cerevisiae. *Yeast* **14,** 953–961 (1998).
31. Granneman, S., Kudla, G., Petfalski, E. & Tollervey, D. Identification of protein binding sites on U3 snoRNA and pre-rRNA by UV cross-linking and high-throughput analysis of cDNAs. *Proc. Natl. Acad. Sci. U.S.A.* **106,** 9613–9618 (2009).
32. Storici, F. & Resnick, M. A. The delitto perfetto approach to in vivo site-directed mutagenesis and chromosome rearrangements with synthetic oligonucleotides in

yeast. *Meth. Enzymol.* **409,** 329–345 (2006).
33. Tollervey, D. & Mattaj, I. W. Fungal small nuclear ribonucleoproteins share properties with plant and vertebrate U-snRNPs. *EMBO J* **6,** 469–476 (1987).
