# Supplementary material for: Kinetic CRAC uncovers a role for Nab3 in determining gene expression profiles during stress
Source: Nat Commun. 2017 Apr 11;8:12. doi: 10.1038/s41467-017-00025-5 (PMC5432031; doi:10.1038/s41467-017-00025-5)
Supplement: Supplementary file 2 — Reviewer reports and authors' response from the peer review of this Article at Nature Communications [file 41467_2017_25_MOESM2_ESM.pdf]

PEER REVIEW FILE

Reviewers' Comments:

Reviewer #1 (Remarks to the Author)

NCOMMS-16-22634-T

UV crosslinking has emerged as an informative method for the investigation of RNA-protein interactions. Since UV crosslinking is relatively inefficient, it is frequently necessary to employ extended crosslinking times to reach the required dosages. As a consequence, fast kinetics cannot be followed and secondary, stress-induced effects may perturb the biological analyses. In this manuscript, van Nues et al. report the construction of a new UV crosslinking device, named 'Vari-X-linker', which allows the application of more intensive UV light to biological samples, reaching the necessary dosage much (5-10 fold) faster. Although it remains to be assessed whether this reduces DNA damage (see below), it certainly leaves less time for the manifestation of secondary effects. Overall, the reported data support the authors' conclusion that the 'Vari-X-linker' offers a significant technical advance.

Assessment the yeast RBP Nab3 in response to glucose starvation is used to demonstrate the benefits of the 'Vari-X-linker' applying minute-scale kinetics. While interesting response patterns are being described and interpreted, the work lacks experimental depth. The main achievement of this work is hence unrelated to the title of the manuscript, but rather the invention of a very useful new UV crosslinking device. Since Nature Communications focuses on biology rather than engineering, I fail to recommend this manuscript for publication.

Detailed comments:
1) The authors appear to interpret alterations observed after a change in experimental conditions as purposeful "regulation". Unless functional experiments document that observed changes are required to achieve a particular biological outcome, they should be seen as "induced changes" and not be termed "regulation".
2) How specific is the "dynamic binding" of Nab3 in comparison to other RNA-binding proteins? I would expect that many RBPs would display altered RNA binding in response to glucose starvation, some of these as a bystander effect rather than purposeful regulation
3) The authors seem to suggest that the 'Vari-X-linker' causes less DNA damage. I doubt that this is the case. I consider it more likely that the shorter time courses used with the 'Vari-X-linker' leave less time for DNA damage to manifest in a response that profoundly affects the transcriptome. Direct quantitative assessment of pyrimidine dimers or DNA breaks would be

required to draw conclusions about differences in the extent of DNA damage
4) In their laudable attempt to assess the functional role of Nab3, the authors use the rapamycin-regulated 'anchor-away system'. As far as I can see, controls that help distinguish between a primary drug effects of rapamycin and the desired effect of Nab3 degradation are lacking. The experiment lacks a "rescue control" with a rapamycin-insensitive form of Nab3. Moreover, the determination of "Pol II escape indices" appear to be a rather indirect way to assess the function of Nab3 on the protein-coding transcripts and retrotransposons.
5) I like the suggestions at the end of the discussion regarding possible future questions that could be addressed using the 'Vari-X-linker'.

Minor point:
1) The title of the manuscript is misleading. The manuscript does not address "the yeast protein-RNA interactome", but rather reports an analysis of altered RNA-binding of a given RBP in response to glucose starvation.

Reviewer #2 (Remarks to the Author)

van Nues et al. report on a new method to characterize the transcriptome to which the yeast RNA-binding protein Nab3 is bound. They report higher temporal resolution (on the order of a few minutes) using a custom UV irradiation device. Changes in Nab3 were categorized and were largely independent of transcript levels. The presence of Nab3 at stress related genes and retrotransposons seemed to 'dampen' their expression.

The authors show convincingly that their xCRAC method detects rapid RNA-protein cross linking events and reveals recruitment of Nab3 to RNA following glucose restriction, a stimulus previously shown to initiate a rearrangement of this protein and its dimerization partner, Nrd1, in conjunction with a change in the transcriptome profile (Darby et al., 2012). The paper would benefit from a more mechanistic interpretation of many of the findings, including the four (k-means) temporal classes of genes/transcripts bound by Nab3 (Fig. 4). In general, a deeper interpretation of the large body of data would illuminate for the reader what the authors think the significance of the findings are; since they are in many ways a technical improvement over a previous version of the experiment. For example, is the pattern seen in Fig. 4D of the loss of pol II from chromatin following glucose starvation, causally related to the termination activity of the burst of Nab3 recruited to the promoter proximal region? That is, is premature termination causing the changes in polymerase occupancy that is observed? Is the loss of Nab3 by the anchor-away manipulation leading to polymerase reading into the body of genes because early termination now doesn't occur? The change in glucose-dependent Nab3 binding observed in Fig. 5C appears modest. However, the strong example of ENO1's shift in Nab3 binding to downstream sites in the body of the gene following glucose removal (Fig. 5D) is interesting. It would seem to represent a more complex regulatory mechanism, perhaps a start site shift, as

alluded to in the text. How this would work is unclear. Some simple start site mapping and northern blotting experiments could flesh this out, but in any case, does the mechanism employ Nab3's function as a termination factor in conjunction with start site changes? If so, the mechanism might be similar to the control of IMD2 (Dichtl, Mol Cell, 2008). The authors remain somewhat agnostic in their interpretation, but their idea is testable. Additional reference throughout the paper back to the four classes of crosslinking (Fig. 4A) is also desirable. From which category were subsequent examples and analyses drawn? Does it matter? Similarly, some discussion as to why Ty2's distinct profile that makes it stand out from the other Tys would be welcome.

Summary: This is a technically very nice paper that expands on prior work and which would benefit from more context and data analysis.

Minor comments-
-suggest deleting 'already' on line 28.
-suggest changing 'intrinsically' on line 65 to 'limitation'
-suggest changing 'time' on line 82 to 'temporal'
-suggest changing 'efficient' to 'efficiently' on line 101.
-please specify the antibody used to identify Nab3 on Western blots. Is it through the protein A part of the tag?
-"Vary" on line 105 should be "Vari".
-line 120: a citation to Fig. S2b is offered but presumably this should be Fig. 2B.
-line 188 cites Fig. 2B regarding sn/snoRNAs but they do not appear enriched, perhaps a typo.
-line 329 Nab3 should be referred to as a transcription termination factor, not a terminator.

Reviewer #3 (Remarks to the Author)

In this study, van Nues et al. attempt to improve time resolution of CLIP techniques. Current techniques require long UV crosslinking times, however, this causes both damage and poor time resolution. UV damage can induce DNA damage responses in the cell that may interfere with the phenomenon of interest and many RNA binding proteins may alter their binding profile quickly upon induction of a different stimulus. Both of these issues can be mitigated with higher efficiency crosslinking. van Nues et al. have designed a shutter system with a UV crosslinker that allow them to leave the UV bulb on mitigating time loss by warming up the lamp allowing them to get similar crosslinking efficiency to current methods at a much faster rate. They then use this technique they refer to as χCRAC to characterize changes in RNA-binding of Nab3, a transcription termination factor, and RNA Polymerase II quickly after induction of stress conditions finding new roles for Nab3 in regulating transposon activity and rapidly changing RNA binding partners.

The paper is high-quality overall and provides a powerful adaptation that can overcome major limitations in current technologies.

Major comments:

1. Figures 2.
Based on the increase in transcripts crosslinked to Nab3 in the Megatron vs. the Vari-X-linker (2B, 2E) involved in transposition and DNA repair, they claim that the Megatron induces more DNA damage that influences the results of CLIP experiments.
There should be a direct measure of DNA damage under both of the crosslinking conditions. It is important to quantitate the improvements over the Megatron as the limited amount of damage from UV is a large part of the improvement of this system over existing technology.
Furthermore, the increase in crosslinked retrotransposon transcripts in the Nab3 dataset shown in figure 2 could represent an increase in specificity of Nab3 for these transcripts rather than a fast response to greater UV damage. This could be readily addressed with qPCR for retrotransposon transcripts after crosslinking in both conditions.

2. Figure 3.
The authors cut out a region above the main band with Nab3 to reduce technical noise. How does this compare to the main band, and does this main band represent RNA that is relevant to Nab3 response to different stress conditions?

3. Figure 6.
For the qPCR experiments in 6C, there should be some indication that Nab3 is not regulating Act1 or that PolII has a similar binding profile to Act1 transcripts indicating similar levels of Act1 to control for potential varying levels of Act1. This data should already exist based on their experiments.
4. There should be a negative control of a with differential expression under the stress condition but no regulation by Nab3 to show that repression of these genes in the rapamycin (and thus absence of Nab3) condition is Nab3 specific in 6C. Or does Nab3 globally control all stress response genes?

5. Finally, was the rapamycin treatment performed in the stress condition, or was it prior to stress? If it was during the stress condition, then the effectiveness of the treatment should be validated under both conditions.

6. The figure legend should discuss the rapamycin treatment in 6C.

Minor comments:
There are also some minor grammatical and organizational considerations.

The title needs to be the same in all documents (one of the supplemental documents is titled Quantitatively measuring protein-RNA interaction dynamics in vivo at minute time-point resolution).

In line 40 of the supplemental data section, “tot” should be changed to “to”.

The length of each glucose starvation incubation should be made more clear in several of the figures.

Reviewers' comments:

Reviewer #1 (Remarks to the Author):

NCOMMS-16-22634-T

UV crosslinking has emerged as an informative method for the investigation of RNA-protein interactions. Since UV crosslinking is relatively inefficient, it is frequently necessary to employ extended crosslinking times to reach the required dosages. As a consequence, fast kinetics cannot be followed and secondary, stress-induced effects may perturb the biological analyses. In this manuscript, van Nues et al. report the construction of a new UV crosslinking device, named 'Vari-X-linker', which allows the application of more intensive UV light to biological samples, reaching the necessary dosage much (5-10 fold) faster. Although it remains to be assessed whether this reduces DNA damage (see below), it certainly leaves less time for the manifestation of secondary effects. Overall, the reported data support the authors' conclusion that the 'Vari-X-linker' offers a significant technical advance.

Assessment the yeast RBP Nab3 in response to glucose starvation is used to demonstrate the benefits of the 'Vari-X-linker' applying minute-scale kinetics. While interesting response patterns are being described and interpreted, the work lacks experimental depth. The main achievement of this work is hence unrelated to the title of the manuscript, but rather the invention of a very useful new UV crosslinking device.

Since Nature Communications focuses on biology rather than engineering, I fail to recommend this manuscript for publication.

We respectfully disagree. Nature communications focuses on all kinds of research areas, including physics, chemistry, earth sciences and mathematics. Secondly, the kinetic CRAC method is not solely built around the Vari-X-linker. We also made a large number of changes to the original CRAC protocol to make these short time-course measurements possible and developed mathematical modeling methods to analyze the data. This is discussed in detail on pages 7 and 8 of the revised manuscript and in the Methods section. Thus, χCRAC is the combined result of engineering, biology and statistics. Without this we would not have been able to perform kinetic CRAC with high accuracy.

Detailed comments:

1. The authors appear to interpret alterations observed after a change in experimental conditions as purposeful "regulation". Unless functional experiments document that observed changes are required to achieve a particular biological outcome, they should be seen as "induced changes" and not be termed "regulation".

   We agree, we have now been more careful with our interpretation of the data and have used the words "induced changes" rather than "regulation" in the text when referring to Nab3 function.

2. How specific is the “dynamic binding” of Nab3 in comparison to other RNA-binding proteins? I would expect that many RBPs would display altered RNA binding in response to glucose starvation, some of these as a bystander effect rather than purposeful regulation

   We have also generated some preliminary data on Nrd1 (another component of the NNS complex) and the cytoplasmic exonuclease Xrn1. These data nicely show that, as expected, Nrd1 and Nab3 show similar changes in binding profiles. Xrn1, however, generally shows a different profile that is more similar to our RNASeq data, which we generated from total RNA isolated from samples from several time-points. An example is shown in Fig. R1.1 at the bottom of this rebuttal. As expected, both Nrd1 and Nab3 show very similar and reproducible changes in their binding profile to the *ILV5* transcript. However, the Xrn1 binding profile is quite different.
   We agree that we cannot exclude that a change in RNA binding profiles could be the result of a bystander effect. However, the main message of our story is that we can detect rapid changes in RNA binding profiles during stress, which we believe we show very convincingly. To demonstrate whether or not the change in binding profile is the result of a bystander effect is not trivial and would go beyond the scope of this manuscript. We are currently testing many more RNA-binding proteins but it will take several years for these studies to be completed. In the meantime, we would prefer to publish the method so that other groups can start using it.

3. The authors seem to suggest that the ‘Vari-X-linker’ causes less DNA damage. I doubt that this is the case. I consider it more likely that the shorter time courses used with the ‘Vari-X-linker’ leave less time for DNA damage to manifest in a response that profoundly affects the transcriptome. Direct quantitative assessment of pyrimidine dimers or DNA breaks would be required to draw conclusions about differences in the extent of DNA damage

   We apologize for the confusion; it was not our intention to give the reader the impression that there is less DNA damage in the Vari-X-linker. On page 6 we state that a short UV irradiation time “*minimizes the onset of DNA damage response*”, to which we refer the transcriptional responses associated with DNA damage. We completely agree that during such short UV irradiation times the cells simply do not have sufficient time to mount a DNA damage stress response, reducing the noise. To make this more explicitly clear in the text we now write on page 6 of the revised manuscript:
   “While these data suggest a novel role for the NNS complex in regulating DNA damage response and suppressing retrotransposon transcription (also see below), it also illustrates that long UV irradiation times significantly increase the likelihood of detecting alterations in transcription that are the result of the activation of the DNA damage response.”

4. In their laudable attempt to assess the functional role of Nab3, the authors use the rapamycin-regulated ’anchor-away system’. As far as I can see, controls that help distinguish between a primary drug effects of rapamycin and the desired effect of Nab3 degradation are lacking. The experiment lacks a “rescue control” with a rapamycin-insensitive form of Nab3.

This is an excellent point. To address this concern we have performed Pol II χCRAC experiments using the rapamycin insensitive parental strain in which Rpo21 was HTP-tagged. Cells were grown in glucose to exponential phase and incubated with rapamycin or the solvent (ethanol) for one hour. We crosslinked rapamycin treated cells grown in glucose and cells that were shifted to medium lacking glucose for 14 minutes. These results have now been added to Supplementary Figures 6 and 10. Treating the parental strain with rapamycin did not show accumulation of 3' extended CUTs or 3' extended snoRNAs (such as snR13; Supplementary Figure 6c). Moreover, we demonstrate that the changes in Pol II distribution observed for the genes discussed in the main text (*PIC2*, *MAL33*, *NRD1*, *LST8*, *RHO5*, *IMD3*) does not change when treating the parental anchor-away strain with rapamycin for one hour (Supplementary Figure 10).

Collectively, these data demonstrate that the observed effects on Pol II distribution are a direct effect of nuclear depletion of Nab3.

5. Moreover, the determination of "Pol II escape indices" appear to be a rather indirect way to assess the function of Nab3 on the protein-coding transcripts and retrotransposons.

   We would argue that it is actually a very direct and reliable approach to identify genes that are potentially regulated by Nab3. Nab3 functions by terminating Pol II transcription and when looking at Pol II distribution of genes regulated by Nab3 very strong Pol II peaks can often be observed around the Nab3 binding sites, which is indicative of Pol II pausing induced by Nab3 binding to its RNA target. Therefore, the expectation would be that upon Nab3 depletion, these pause sites would disappear and that more Pol II would accumulate in regions downstream of Nab3 binding sites. This is exactly what we are seeing and it is essentially what we are measuring when looking at escape indices. The escape index method has previously been successfully applied by Patrick Cramer's group to identify novel genes that are attenuated by Nrd1, a component of the NNS complex (Schulz et al *Cell* 155**,** 1075–1087). This also offered us a way to directly compare our data with their work. The fact that we are picking up more or less the same genes in our glucose dataset gives us the confidence that most of the genes that we identified in our analysis are *bona fide* Nab3 targets. We also manually inspected each target in the genome browser to make sure that the algorithm made the correct call. Finally, some of these potential targets were validated by qRT-PCR and the data agree very well with our EI analysis.

6. I like the suggestions at the end of the discussion regarding possible future questions that could be addressed using the 'Vari-X-linker'.

   We fully agree! We hope to be in the position to address these questions in the very near future.

Minor point:

1. The title of the manuscript is misleading. The manuscript does not address "the yeast protein-RNA interactome", but rather reports an analysis of altered RNA-binding of a given RBP in response to glucose starvation.

   We agree. Considering that the focus of the manuscript is now a bit more on biology and the function of Nab3, we changed the title into: *"Kinetic CRAC uncovers a role for Nab3 in determining gene expression profiles during stress."*. We hope this reviewer agrees that this is a more suitable title.

Reviewer #2 (Remarks to the Author):

van Nues et al. report on a new method to characterize the transcriptome to which the yeast RNA-binding protein Nab3 is bound. They report higher temporal resolution (on the order of a few minutes) using a custom UV irradiation device. Changes in Nab3 were categorized and were largely independent of transcript levels. The presence of Nab3 at stress related genes and retrotransposons seemed to 'dampen' their expression.

1. The authors show convincingly that their xCRAC method detects rapid RNA-protein cross linking events and reveals recruitment of Nab3 to RNA following glucose restriction, a stimulus previously shown to initiate a rearrangement of this protein and its dimerization partner, Nrd1, in conjunction with a change in the transcriptome profile (Darby et al., 2012). The paper would benefit from a more mechanistic interpretation of many of the findings, including the four (k-means) temporal classes of genes/transcripts bound by Nab3 (Fig. 4).

   We have now included a more comprehensive analysis of the four Nab3 clusters and have provided mechanistic interpretations for many of our findings. In the new Figure 5b we show which RNA classes are bound by Nab3 in each cluster. Interestingly, the data indicate that those transcripts that show a very strong reduction in Nab3 binding predominantly include non-coding RNAs, whereas transcripts that show a strong increase in Nab3 binding primarily contain protein-coding genes as well as some tRNAs (see new Figure 5b). These appear to be predominantly mature tRNAs (having 3' CCA) and therefore we speculate that this might be Nab3 binding to tRNAs that are reimported into the nucleus upon glucose starvation. We also compared the Nab3 clusters with four Pol II K-means clusters generated from all the transcripts (new Fig. 5d) and find that at later stages of the adaptation process Nab3 generally follows Pol II transcription. We now also discuss a few examples of Nab3-dependent termination of anti-sense transcription during stress (see new Figure 5e and Supplementary Figure 7).

2. In general, a deeper interpretation of the large body of data would illuminate for the reader what the authors think the significance of the findings are; since they are in many ways a technical improvement over a previous version of the experiment.

We now provide a more detailed interpretation of our results as well as discuss the biological significance of our data. Besides the additional experiments and analyses mentioned in point 1, we have done more detailed analyses on our Pol II χCRAC data and compared it to RNASeq data generated form the time-course (see Fig. 4c-e). Our analyses suggest that a rate-limiting step in glucose starvation adaptation could be RNA decay. We have also done more research to figure out how *ENO1* transcription is regulated (see qPCR data in Figure 6, the Supplementary data and the new Supplementary Figures 8 and 12). We also show new data on the Ty1 and Ty2 retrotransposons in Figure 9 and discuss in more depth how we believe Ty1 and Ty2 retrotransposon expression is controlled by Nab3. We have added a new Figure 10 that shows a number of gene expression models and we explain how, based on our data, Nab3 could contribute to regulating gene expression during stress.

For example, is the pattern seen in Fig. 4D of the loss of pol II from chromatin following glucose starvation, causally related to the termination activity of the burst of Nab3 recruited to the promoter proximal region? That is, is premature termination causing the changes in polymerase occupancy that is observed? Is the loss of Nab3 by the anchor-away manipulation leading to polymerase reading into the body of genes because early termination now doesn't occur?

In these two cases unfortunately not. We have looked at our Nab3 depletion data and although we did find oligo-A tailed reads in the data, we could not find convincing evidence that Nab3 binding during the first four minutes of glucose starvation induces changes in the expression kinetics of *ILV5* and *RPP0*. We now mention this in the Discussion session and provide possible explanations for these observations.
Although this is a negative result, we do feel it is important to showcase these two examples in the paper as they nicely illustrate how our method can detect rapid changes in protein-RNA interactions.

3. The change in glucose-dependent Nab3 binding observed in Fig. 5C appears modest. However, the strong example of ENO1's shift in Nab3 binding to downstream sites in the body of the gene following glucose removal (Fig. 5D) is interesting. It would seem to represent a more complex regulatory mechanism, perhaps a start site shift, as alluded to in the text. How this would work is unclear. Some simple start site mapping and northern blotting experiments could flesh this out, but in any case, does the mechanism employ Nab3's function as a termination factor in conjunction with start site changes? If so, the mechanism might be similar to the control of IMD2 (Dichtl, Mol Cell, 2008). The authors remain somewhat agnostic in their interpretation, but their idea is testable.

   We agree. We have now looked into transcription regulation of *ENO1* in quite a lot more detail (see novel Supplementary Figures 8 and 12) and have done a number of qPCR experiments to back up some of our conclusions. We discovered that regulation of *ENO1* is very complex and involves several transcription factors. Our qPCR results show that the upstream CUT is very low in abundance. When looking at the transcription factor (TF) ChIP-exo data

we find that, relative to the orthologue *ENO2,* very low levels of general TFs bind near the *ENO1* TSS (Supplementary Figure 8). Thus our current working hypothesis is that transcription of the upstream CUT could interfere with initiation at the *ENO1* TSS, possibly by inducing changes in histone modifications that are incompatible with PIC assembly. What is interesting is that the CUT initiates from a site referred to as the Upstream Repressor element (URS), which is bound by the transcription factors Reb1 and Tye7. The latter binds just upstream of the *ENO1* TATA-box and an allele of Tye7 (SGC1-1) has been described which does not show *ENO1* suppression on glucose, suggesting that this protein contributes strongly to repress transcription from this locus. Reversal of the URS, which would redirect CUT transcription and replace Tye7, also lifts transcription-suppression of *ENO1* on glucose. We now mention our findings in the Results section and the Discussion and provide a more detailed discussion in the Supplementary Data. It is a very interesting and also very complicated regulatory system.

4. Additional reference throughout the paper back to the four classes of crosslinking (Fig. 4A) is also desirable. From which category were subsequent examples and analyses drawn? Does it matter?

   As mentioned in the response to the first point raised by the reviewer, we have added a new panel to Figure 5 (5d) where we compare Nab3 profiles with the Pol II profiles of all the transcripts. Some of the genes that we discuss in the text are highlighted in this figure so that the reader can see to which category of genes they belong to. We hope that this makes the story as well as our reasoning for picking these genes easier to follow. Not all genes that we picked up from our escape index analyses (for example, *MAL33*) could be classified into groups as they did not fall into the four k-means clusters.

5. Similarly, some discussion as to why Ty2's distinct profile that makes it stand out from the other Tys would be welcome.

   A very good point. We have done more extensive analysis on the retrotransposon data and have found a possible explanation for why Ty1 and Ty2 transcription kinetics are so different upon Nab3 depletion. The results of these analyses have now been added to Figure 9 and are discussed in detail on pages 17-19 in the main text. What is really interesting is that Ty1 and Ty2 retrotransposons have a very different Nab3 cross-linking profile. In Ty1 retrotransposons Nab3 seems to cross-link throughout the transcripts, whereas in Ty2 Nab3 cross-links mainly in a region with a very high concentration of Nab3 binding sites.
   Although Pol II is roughly evenly distributed on these retrotransposons when cells are grown in glucose, in glucose deprived cells there is very clear pausing of Pol II around the major Nab3 cross-linking sites. In the case of Ty2 this is the clearest (Figure 9h, compare panels IV and VI). Virtually all the Pol II piles up near the main Nab3 cross-linking region, strongly suggesting Pol II pausing as a result of Nab3 binding. Thus, we conclude that the difference in Ty1 and Ty2 transcription kinetics in Nab3-depleted cells may be because Ty1 and Ty2 transcripts show very different Nab3 cross-linking profiles. We speculate that Nab3 might more efficiently terminate Ty2 transcription

because of this region that has a very high concentration of Nab3 binding sites.

Summary: This is a technically very nice paper that expands on prior work and which would benefit from more context and data analysis.

We thank this reviewer for providing very useful comments and constructive criticism!

Minor comments-
-suggest deleting ‘already’ on line 28.

Corrected

-suggest changing ‘intrinsically’ on line 65 to ‘limitation’

Corrected

-suggest changing ‘time’ on line 82 to ‘temporal’

Corrected

-suggest changing ‘efficient’ to ‘efficiently’ on line 101.

Corrected

-please specify the antibody used to identify Nab3 on Western blots. Is it through the protein A part of the tag?

We have now added a detailed Western blot analyses section to the Methods describing the antibodies we used to detect the tagged proteins. The anti-TAP antibody we used recognized the spacer between the TEV cleavage site and the six histidines in the tag. This particular antibody was initially produced to detect TAP tags but we have included the same recognition site in our HIS6-TEV-PROTA (HTP tag; Nab3 and Rpo21) and HIS6-TEV-3xFLAG tags.

-“Vary” on line 105 should be “Vari”.

Corrected

-line 120: a citation to Fig. S2b is offered but presumably this should be Fig. 2B.

Corrected

-line 188 cites Fig. 2B regarding sn/snoRNAs but they do not appear enriched, perhaps a typo.

This section has changed and the sentence has been removed

-line 329 Nab3 should be referred to as a transcription termination factor, not a terminator.

Fixed throughout the paper.

Reviewer #3 (Remarks to the Author):

In this study, van Nues et al. attempt to improve time resolution of CLIP techniques. Current techniques require long UV crosslinking times, however, this causes both damage and poor time resolution. UV damage can induce DNA damage responses in the cell that may interfere with the phenomenon of interest and many RNA binding proteins may alter their binding profile quickly upon induction of a different stimulus. Both of these issues can be mitigated with higher efficiency crosslinking. van Nues et al. have designed a shutter system with a UV crosslinker that allow them to leave the UV bulb on mitigating time loss by warming up the lamp allowing them to get similar crosslinking efficiency to current methods at a much faster rate. They then use this technique they refer to as χCRAC to characterize changes in RNA-binding of Nab3, a transcription termination factor, and RNA Polymerase II quickly after induction of stress conditions finding new roles for Nab3 in regulating transposon activity and rapidly changing RNA binding partners.

The paper is high-quality overall and provides a powerful adaptation that can overcome major limitations in current technologies.

We thank the reviewer for his/her constructive criticism!

Major comments:

1. Figures 2.
   Based on the increase in transcripts crosslinked to Nab3 in the Megatron vs. the Vari-X-linker (2B, 2E) involved in transposition and DNA repair, they claim that the Megatron induces more DNA damage that influences the results of CLIP experiments.
   There should be a direct measure of DNA damage under both of the crosslinking conditions.

   It was not our intention to give the reader the impression that there is more DNA damage in the Megatron compared to the Vari-X-linker. We apologize for the confusion. We assume that the DNA damage is roughly the same, however, within the 5-10 second time-frame, we believe that the cells do not have sufficient time to mount a DNA damage response. Therefore, shorter cross-linking times can greatly reduce the likelihood of picking up changes in transcription that are linked to the DNA damage response. To make this more explicitly clear in the text we now write on page 6 of the revised manuscript: *"While these data suggest a novel role for the NNS complex in regulating DNA damage response and suppressing retrotransposon transcription (also see below), it also illustrates that long UV irradiation times significantly increase the likelihood of detecting alterations in transcription that are the result of the activation of the DNA damage response."*

2. It is important to quantitate the improvements over the Megatron as the limited amount of damage from UV is a large part of the improvement of this system over existing technology. Furthermore, the increase in crosslinked retrotransposon transcripts in the Nab3 dataset shown in figure 2 could

represent an increase in specificity of Nab3 for these transcripts rather than a fast response to greater UV damage. This could be readily addressed with qPCR for retrotransposon transcripts after crosslinking in both conditions.

As requested by this reviewer, we have performed qRT-PCR on Ty1 and Ty2 retrotransposons on RNA isolated from cells cross-linked in the Megatron and Vari-X-linker. The data, shown in the new Supplementary Figure 2, clearly show that during the UV irradiation, steady state levels of these transcripts does not change significantly. We therefore agree with this reviewer that the observed changes in Nab3 binding was likely the result of an increase in specificity of Nab3 for these transcripts. Even though the longer UV irradiation times do not appear to alter Ty1 transcript levels, it still means that the differences in Nab3 binding that we are measuring are the result of differences in UV irradiation time. Interestingly, we also see significantly more Nab3 binding when cells are deprived of glucose. This suggests that Nab3-dependent termination of retrotransposons might increase under a variety of stress conditions. We now mention these findings on page 6 of the revised manuscript. Therefore, with the Megatron you are much more likely to pick up changes in cross-linking profiles that are a direct result of prolonged UV irradiation exposure.

3. Figure 3.
   The authors cut out a region above the main band with Nab3 to reduce technical noise. How does this compare to the main band, and does this main band represent RNA that is relevant to Nab3 response to different stress conditions?

   From our experience with CRAC experiments we have learned that the main band mainly contains short RNA fragments and these often are either too short to map to the reference sequence or will map to multiple locations in the genome. To avoid this, we now extract RNA from regions that should have longer fragments (our average insert size is about 80-100 bp). To get a higher resolution of the protein binding sites, we specifically look for regions that have a high frequency of deletions. When we treat the cross-linked RNP complex with protease K to remove the protein generally the amino acids that are cross-linked to the RNA remain attached. During the reverse transcription step, when the RT bumps into the site of cross-linking it very frequently introduces deletions in the cDNA. Therefore, to get a higher resolution of the protein-binding sites, we then focus our analysis on finding regions that have a high frequency of deletions.

4. Figure 6.
   For the qPCR experiments in 6C, there should be some indication that Nab3 is not regulating Act1 or that Pol II has a similar binding profile to Act1 transcripts indicating similar levels of Act1 to control for potential varying levels of Act1. This data should already exist based on their experiments.

   We have now included a genome browser snapshot of the *ACT1* gene showing Pol II profiles in control (ethanol-treated) or Nab3-depleted (rapamycin-treated) cells in Supplementary Figure 9a. These results show

that the *ACT1* Pol II profile is very similar in the wild-type vs Nab3 depleted strain. We also show that rapamycin alone does not affect *ACT1* Pol II profiles in the new Supplementary Figure 10.

5. There should be a negative control of a with differential expression under the stress condition but no regulation by Nab3 to show that repression of these genes in the rapamycin (and thus absence of Nab3) condition is Nab3 specific in 6C. Or does Nab3 globally control all stress response genes?

   We completely agree that this would be a good control. The problem we had is that our data showed that Nab3 binds to thousands of mRNAs both in glucose and medium lacking glucose. Frustratingly, we actually could not find a convincing example of a gene that showed a significant increase in PolI II transcription but *no* significant Nab3 binding. There are always some Nab3 binding sites in transcripts and if the cell produces high enough levels of the transcript, at some point you will detect some Nab3 binding. This does not necessarily mean this is functional or will have a significant impact on expression levels.
   To demonstrate that the observed changes in Pol II profile was a direct consequence of Nab3 depletion and not induced by the drug rapamycin itself, we did the following:

   (I) we went back to our escape index analyses and picked a number of genes that are strongly upregulated during glucose starvation, but which did not show a major change in Pol II occupancy or changes in Pol II profile upon Nab3 depletion. These included *GLK1*, *YBR085C-A* and *LST8*. We subsequently did qRT-PCR analyses on cells grown in glucose and cells shifted to medium lacking glucose for 20 and 40 minutes. These results are now shown in the revised Figure 7. We show that rapamycin induced nuclear depletion of Nab3 does not have a significant effect on the expression levels of *GLK1* and *YBR085C-A* during the 40-minute time-course, demonstrating that rapamycin does not generally induce an increase in gene expression. For *LST8* we do see a modest (but significant) increase in *LST8* expression upon Nab3 depletion and for this gene we also observed slightly higher levels of Pol II downstream of the Nab3 binding sites (Supplementary Figure 9f).

   (II) We also performed an experiment with the parental anchor away strain expressing a HTP-tagged Rpo21 Pol II component. This strain is insensitive to rapamycin and therefore should not show any changes in the Pol II transcription profile as it does not have Nab3 fused to the FRB domain. The results show that a one-hour treatment of rapamycin indeed does not have any significant effect on the distribution of Pol II on the genes discussed in the main text both in glucose and medium lacking glucose (see new Supplementary Figure 10). Moreover, we also do not detect significant accumulation of 3' extended CUTs or snoRNAs (see revised Supplementary Figure 6a-c).

   In conclusion, we are confident that the observed changes in gene expression are a direct result of Nab3 depletion from the nucleus and not because of transcriptional changes induced by the drug itself.

6. Finally, was the rapamycin treatment performed in the stress condition, or was it prior to stress? If it was during the stress condition, then the effectiveness of the treatment should be validated under both conditions.

   The rapamycin treatment was performed one hour before inducing the stress. However, we also added rapamycin to the medium lacking glucose to make sure that Nab3 would remain in the cytoplasm during the entire time-course. We have made this clearer in the main text on page 8 and on page 26 in the Methods section.

7. The figure legend should discuss the rapamycin treatment in 6C.

   Fixed. We now discuss the experimental procedures (including the length of rapamycin treatment) in the figure legend (now Figure 7c) in more detail.

Minor comments:
There are also some minor grammatical and organizational considerations.

8. The title needs to be the same in all documents (one of the supplemental documents is titled Quantitatively measuring protein-RNA interaction dynamics in vivo at minute time-point resolution).

   Fixed. Note that we have now changed the title to address a comment raised by reviewer 1

9. In line 40 of the supplemental data section, “tot” should be changed to “to”.

   Thank you for pointing out this typo. We have now made the change.

10. The length of each glucose starvation incubation should be made more clear in several of the figures.

    We apologize for not making this sufficiently clear. We now explain in each figure where Nab3 depletion data is shown how long the glucose starvation times were.

Figure R1.1 Alterations in Nab3, Nrd1 and Xrn1 binding to the *ILV5* transcript during glucose deprivation.

(**a**) Nrd1 and Nab3 kinetic CRAC data for the *ILV5* gene. Shown are two biological replicates for each glucose to no glucose (glu to no glu) and one glucose to glucose (glu to glu) control dataset.

(**b**) Same as in (a) but now for Pol II

(**c**) Comparison of Xrn1 kinetic CRAC data with RNASeq data for the *ILV5* transcript.

(**d**) Nab3 and Nrd1 data divided by the Pol II signal. This shows that both Nrd1 and Nab3 binding increase relative to pol II in the first few minutes of glucose starvation. The x-axis shows the time (minutes) after the shift to medium lacking glucose. The y-axis shows log2 transformed FPKM values.

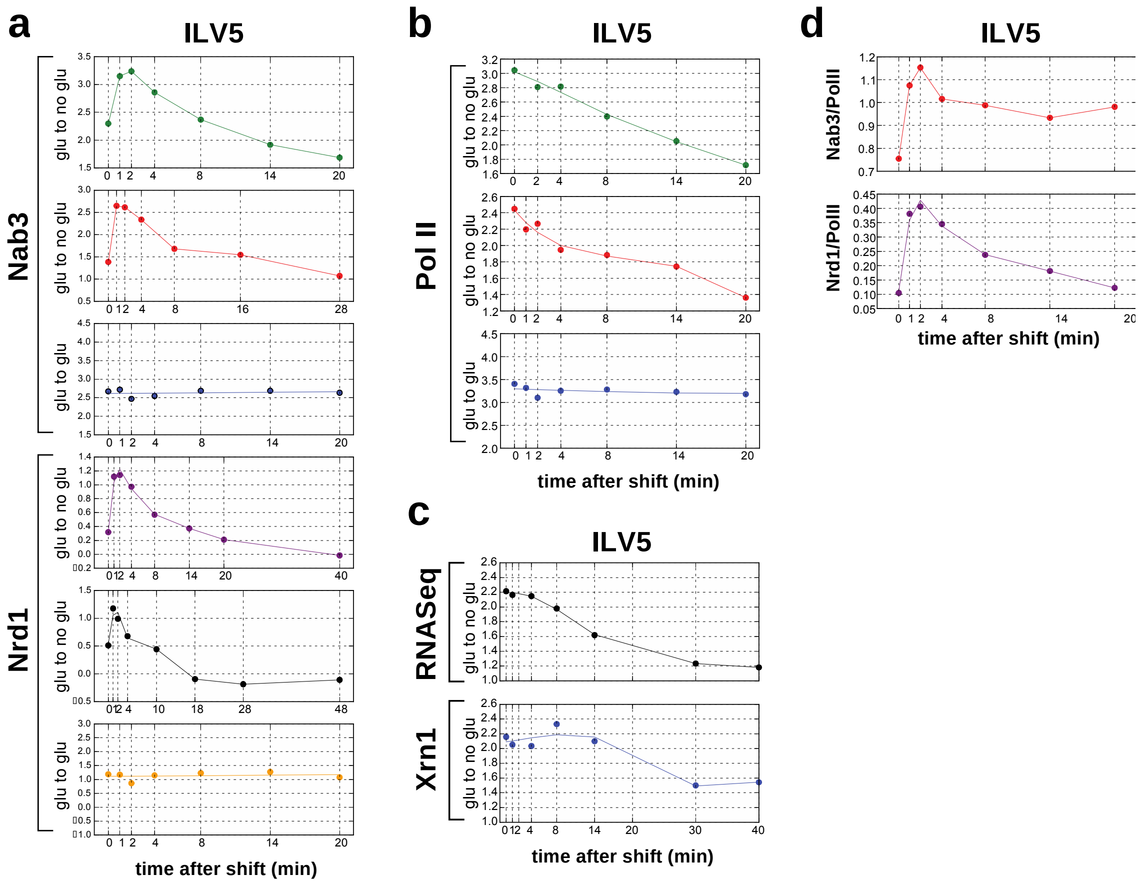

<u>Reviewers' Comments:</u>

Reviewer #1 (Remarks to the Author):

I am happy with the way in which the authors have addressed the points that I had raised.

Reviewer #3 (Remarks to the Author):

The revisions to the original manuscript address all of my major concerns. The new insights into the identity of the Nab3 regulated RNAs and possible mechanisms of regulation add a valuable extra insight. I recommend this manuscript for publication.

1. The new sentence on line 128-132 clarifies that the authors are not claiming there is less DNA damage, but rather an increased DNA damage response with longer crosslinking periods. Perhaps imaging experiments for DNA damage response proteins could help demonstrate this directly, although I do not think this is necessary for publication.
2. I agree with van Nuyes et al that the altered binding profile is due to the UV irradiation times even without large transcriptional changes.
3. OK.
4. The Pol II profiles are indeed very similar.
5. I think these examples address my concerns well. The similar expression of these genes with and without stress in conjunction with similar expression with and without rapamycin in the parent strain suggest Nab3 is specific in the other conditions.
6. All other changes seem thorough.
